# p53 and HuR combinatorially control the biphasic dynamics of microRNA-125b in response to genotoxic stress

**DOI:** 10.1038/s42003-023-04507-9

**Published:** 2023-01-27

**Authors:** Binita Goswami, Deepika Ahuja, David Pastré, Partho Sarothi Ray

**Affiliations:** 1 Department of Biological Sciences, Indian Institute of Science Education and Research, Kolkata, Mohanpur, Nadia, 741246 West Bengal India; 2 SABNP, Univ Evry, INSERM U1204, Université Paris-Saclay, 91025 Evry, France

**Keywords:** miRNAs, DNA damage response

## Abstract

Post-transcriptional regulation of p53, by the microRNA miR-125b and the RNA-binding protein HuR, controls p53 expression under genotoxic stress. p53 mRNA translation is repressed by miR-125b, tightly regulating its basal level of expression. The repression is relieved upon DNA damage by a decrease in miR-125b level, contributing to pulsatile expression of p53. The pulse of p53, as also of HuR, in response to UV irradiation coincides with a time-dependent biphasic change in miR-125b level. We show that the cause for the decrease in miR-125b level immediately post DNA-damage is enhanced exosomal export mediated by HuR. The subsequent increase in miR-125b level is due to p53-mediated transcriptional upregulation and enhanced processing, demonstrating miR-125b as a transcriptional and processing target of p53. p53 activates the transcription of primary miR-125b RNA from a cryptic promoter in response to UV irradiation. Together, these regulatory processes constitute reciprocal feedback loops that determine the biphasic change in miR-125b level, ultimately contributing to the fine-tuned temporal regulation of p53 expression in response to genotoxic stress.

## Abstract (web summary)

miRNA-125b, a translational repressor of p53, undergoes initial HuR-mediated exosomal export in response to DNA damage, leading to p53 upregulation, which in turn induces transcription and processing of miR-125b causing inhibition of p53 expression.

## Introduction

Cells respond to stress by specific alterations in gene expression programs required for the maintenance of homeostasis under adverse conditions^1^. Increasingly, post-transcriptional regulation is found to play an important role in cellular stress responses^2^. Mediators of post-transcriptional control such as non-coding RNAs (ncRNAs) and RNA-binding proteins (RBPs), regulating the translation/turnover of mRNAs, act as important control points of the stress response in eukaryotic cells^3–6^. Especially, the role of microRNAs (miRNAs) as regulators of stress responses has increasingly become apparent^7–9^.

miRNAs are a class of short non-coding RNAs of ~22–24 nucleotide length, that regulate the stability and/or translation of their mRNA targets^10^. Over 60% of mammalian mRNAs are reported to be targets of miRNAs and more than 2300 different miRNAs are estimated in humans^11,12^. miRNA biogenesis and function involves a coordinated series of cellular processing events involving RNA-processing enzymes and RBPs that crosstalk with various mediators of cellular stress responses^13^. Stress-induced alterations in transcription, processing and degradation of miRNAs and interplay between miRNA and RBPs are known to regulate the expression of their mRNA targets^14^. Therefore, alterations in miRNA biogenesis and processing, alterations in mRNA target levels to cross miRNA-defined expression thresholds, target mimicry by competitive-endogenous RNAs (ceRNAs), competition and cooperation between miRNAs and RBPs for target binding, and localization of miRNA and miRNP complexes have all been shown to play important roles in regulating target gene expression in a variety of stress responses^15^. While most studies focus on the tissue or disease-specific up or downregulation of miRNAs, regulatory mechanisms controlling the spatio-temporal dynamics of miRNAs in response to various stressors have remained relatively unexplored.

One of the major hubs of the stress response networks in mammalian cells is the tumor suppressor protein p53^16^. In response to different types of genotoxic stress, such as ionizing and UV radiation, DNA-damaging chemicals and oxidizing free radicals, p53 gets activated to control a series of downstream regulatory processes which are also significantly interconnected with miRNAs^17^. p53 transcriptionally upregulates multiple miRNAs and also enhances the processing of some miRNAs by associating with DDX5, a cofactor of Drosha^18^. In turn, p53 is regulated by the miRNA miR-125b which keeps its basal level of expression tightly repressed^19^. The repression is relieved upon DNA damage by a decrease in miR-125b level through a hitherto unknown mechanism^15^. Therefore, in response to DNA damage, the reversal of miR-125b-mediated translation repression of the p53 mRNA complements the stabilization and activation of p53 protein, resulting in a rapid increase in p53 level which causes transcriptional upregulation of genes involved in cell cycle arrest, DNA damage and apoptosis. Interestingly, the release of miR-125b-mediated translation repression of p53 is facilitated by the RNA-binding protein HuR, which antagonizes miR-125b binding to p53 mRNA in response to UV irradiation^20^. Together, the actions of miR-125b and HuR contribute to a pulsatile expression pattern of p53 in response to DNA damage, thereby allowing a rapid, temporally controlled rise in p53 level and a subsequent decrease, providing the cells a window of opportunity to re-enter the cell cycle if the DNA damage is repaired^21^.

Remarkably, we have observed that the pulsatile change in the p53 level, as also of HuR, in response to UV irradiation coincides with a biphasic change in miR-125b level, opposite in phase to the changes in p53 and HuR levels. We show that the hitherto unknown cause for the decrease in miR-125b level immediately post-DNA damage is exosomal export mediated by HuR, which translocates to the cytoplasm in response to UV irradiation. We further show that the subsequent increase in miR-125b level is due to p53-mediated transcriptional upregulation, demonstrating miR-125b as a transcriptional target of p53. Moreover, p53 also enhances the processing of the primary miR-125b transcript, further increasing the level of mature miR-125b. The exosomal export, transcriptional upregulation and enhanced processing of miR-125b are temporally connected to constitute feedback loops that cause the biphasic change in the miR-125b level. Together, these regulatory processes ultimately contribute to the robust temporal regulation of p53 expression in response to genotoxic stress.

## Results

### Extracellular export, and not transcription downregulation, causes the initial decrease of cellular miR-125b level upon UV irradiation

**Fig. 1:**
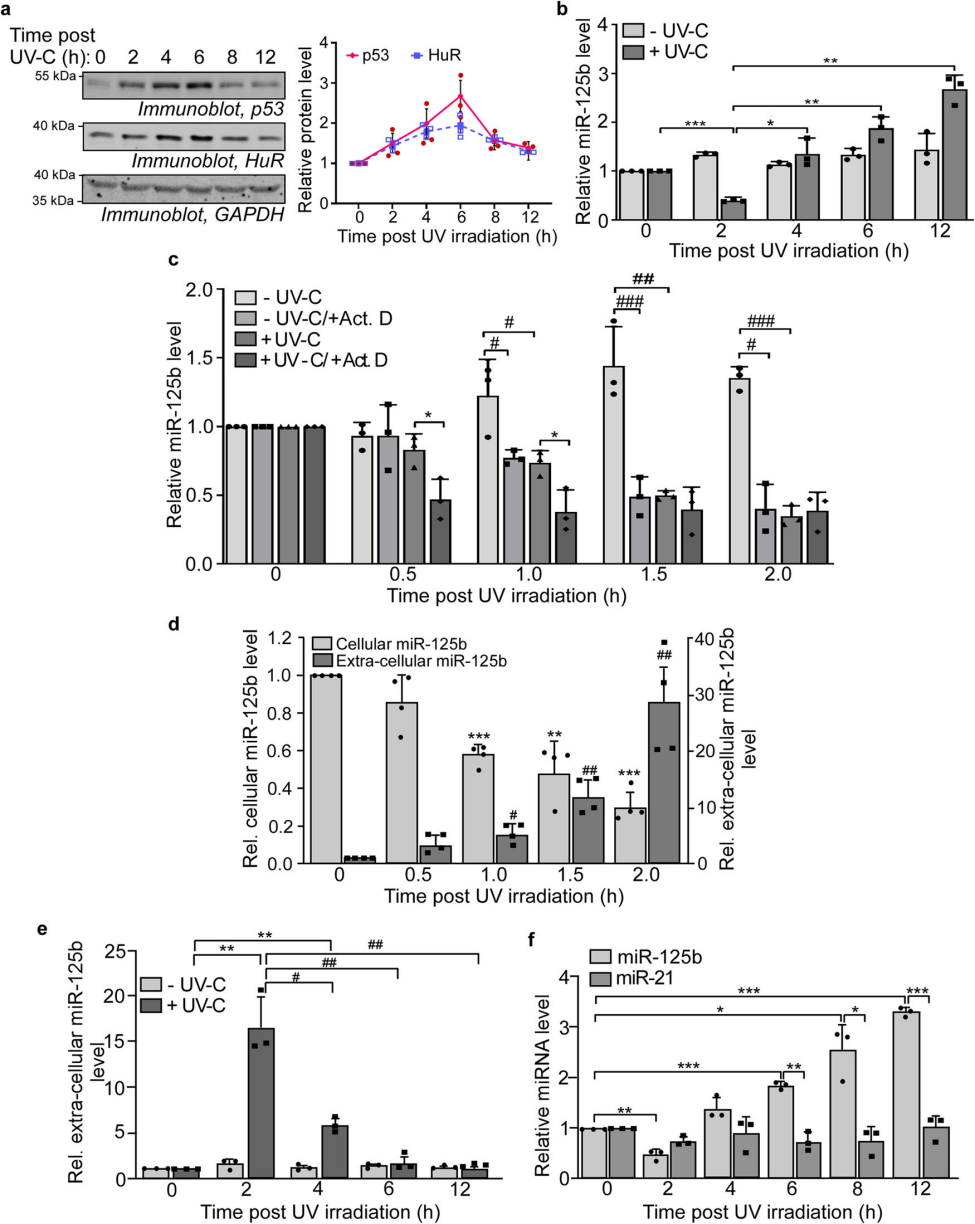
Exosomal export, and not transcriptional downregulation, causes the initial decrease of cellular miR-125b upon UVC irradiation. **a** Representative immunoblots of cytoplasmic lysates from MCF7 cells exposed to 10 J/m^2^ UVC irradiation and collected at different time points post-UVC exposure, with p53, HuR, and GAPDH antibodies (left). Graphical representation of the change of p53 and HuR protein levels, normalized to GAPDH protein levels, over time post-UVC irradiation (right). The data represents mean ± s.d. of normalized band intensities of immunoblots from three independent experiments. **b** qRT-PCR of total RNA isolated from UVC-exposed and unexposed MCF7 cells collected at indicated time points post-UVC exposure using miR-125b specific primers. miR-125b RNA levels were normalized to U6B snRNA levels. Data represent fold change of normalized miR-125b RNA level in UVC-treated and UVC-untreated cells taking 0 h miR-125b RNA level as 1. Mean ± s.d. from three independent experiments are represented. **c** MCF7 cells either untreated or treated with actinomycin D for 2 h were not exposed or exposed to UVC irradiation. qRT-PCR of RNA isolated from cells collected at the indicated time points post-irradiation was done using miR-125b and U6B primers. miR-125b levels were normalized to U6B RNA levels and represented as a fold increase or decrease from 0 h controls for each treatment. Mean ± s.d. from three independent experiments are represented. **d** Estimation of miR-125b from MCF7 cells (cellular miR-125b) and from cell supernatant (extracellular miR-125b) from 0 to 2 h post-UVC irradiation by qRT–PCR. miR-125b levels are represented as fold change from 0 h cellular or extra-cellular miR-125b level. Mean ± s.d. from four independent experiments are represented. **e** Estimation of extracellular miR-125b from cell supernatant from 0 to 12 h post-UVC irradiation by qRT-PCR. Data represent fold change of miR-125b level from respective 0 h controls. Mean ± s.d. from three independent experiments are represented. **f** Estimation of miR-125b and miR-21 from MCF7 cells from 0-12 h post-UVC irradiation by qRT–PCR. miR-125b and miR-21 levels were normalized to U6B RNA levels and represented as fold change from 0 h cellular miR-125b and miR-21 level. Mean ± s.d. from three independent experiments are represented. * # signifies *P* value ≤ 0.05, ** ## signifies *P* value ≤ 0.01 and *** ### signifies *P* value ≤ 0.005 (paired two-tailed *t*-test) in all panels.

Irradiation of MCF7 breast carcinoma cells with 10 J/m^2^ of UV-C radiation causes pulsatile changes of p53 and HuR levels, with the maximum increase at 4–6 h and decreasing to basal level by 12 h post-irradiation (Fig. 1a). We investigated the change of cellular expression of miR-125b during this time period and found a biphasic pattern of miR-125b expression post UV irradiation, with around 80% decrease in miR-125b level at 2 h and then a gradual increase up to ~10-fold in the next 10 h (Fig. 1b). There was no change in miR-125b level during this time period in cells not exposed to UV. The strong decrease in miR-125b level during the initial 2 h post UV exposure may be either due to transcriptional downregulation, degradation, or export of miR-125b. In order to investigate the mechanism of the decrease in miR-125b level, we treated UV-exposed and unexposed cells with the transcription inhibitor Actinomycin D. We then determined cellular miR-125b levels at 30 min intervals till 2 h post UV irradiation. Actinomycin D treatment of cells not exposed to UV caused a decrease in miR-125b levels which approximately corresponded to the decrease in UV-exposed cells, suggesting the possibility that UV caused transcriptional inhibition of miR-125b expression (Fig. 1c). However, Actinomycin D treatment caused a further decrease in miR-125b levels in UV-irradiated cells at 30 min and 1 h post UV treatment, suggesting that transcription of miR-125b continued post UV exposure, which was inhibited by Actinomycin D (Fig. 1c). Therefore, to check whether transcriptional downregulation was at all responsible for the decrease in miR-125b level post UV exposure, we inserted the 525 bp sequence upstream of the miR-125b-1 gene located in 11q24.1, containing the putative miR-125b promoter region (Supplementary Fig. 1a), upstream of a firefly luciferase reporter gene in a promoterless vector. Cells transfected with this construct did not show any decrease in luciferase activity post-UV exposure, suggesting that there was no reduction of miR-125b promoter activity post-UV exposure (Supplementary Fig. 1b). To further validate this observation, we cloned the precursor miR-125b sequence under an RNA pol III promoter (H1) in the vector pSUPER and the miR-125b gene under a heterologous RNA pol II promoter (CMV) in the vector pCDNA3.1. We expressed miR-125b from these plasmids in MCF7 cells in the absence and presence of UV irradiation. Mature miR-125b level increased in the cells which were not subjected to UV irradiation. However, it decreased significantly in the UV-exposed cells, irrespective of the promoter driving miR-125b expression (Supplementary Fig. 1c). Together these observations demonstrated that the decrease of miR-125b level in UV-treated cells was independent of promoter activity, and hence was not due to transcription downregulation.

We then investigated whether the decrease in miR-125b level was due to extracellular export. Extracellular vesicle (EV)-mediated export has been reported previously to export miRNAs from the cell in response to stress^22^. We, therefore, checked the levels of miR-125b both inside cells and in the cell supernatant during the immediate 2 h period post UV irradiation. Cellular miR-125b level showed a steady decrease over 2 h post UV exposure which coincided with a ~30-fold increase in extracellular miR-125b during the same time period (Fig. 1d). This strongly suggested the export of miR-125b as the mechanism responsible for downregulation of its cellular concentration in response to UV. We determined the duration of this export process by checking the extracellular miR-125b level over 12 h post UV exposure. Extracellular miR-125b showed the maximum level 2 h post UV exposure and then declined, suggesting that the export process was temporally controlled and coincided with the reduction in the cellular miR-125b level (Fig. 1e). As multiple miRNAs are reported to undergo extracellular export, we investigated whether UV irradiation caused similar changes in the cellular level of miR-21, another miRNA which is reported to undergo extracellular export^22^. The cellular miR-21 level did not show a significant change over the 12 h period post UV exposure, unlike miR-125b (Fig. 1f). This suggested that UV irradiation specifically induced the export of cellular miR-125b from 0 to 2 h post UV exposure.

### UV irradiation induces the exosomal export of miR-125b

**Fig. 2:**
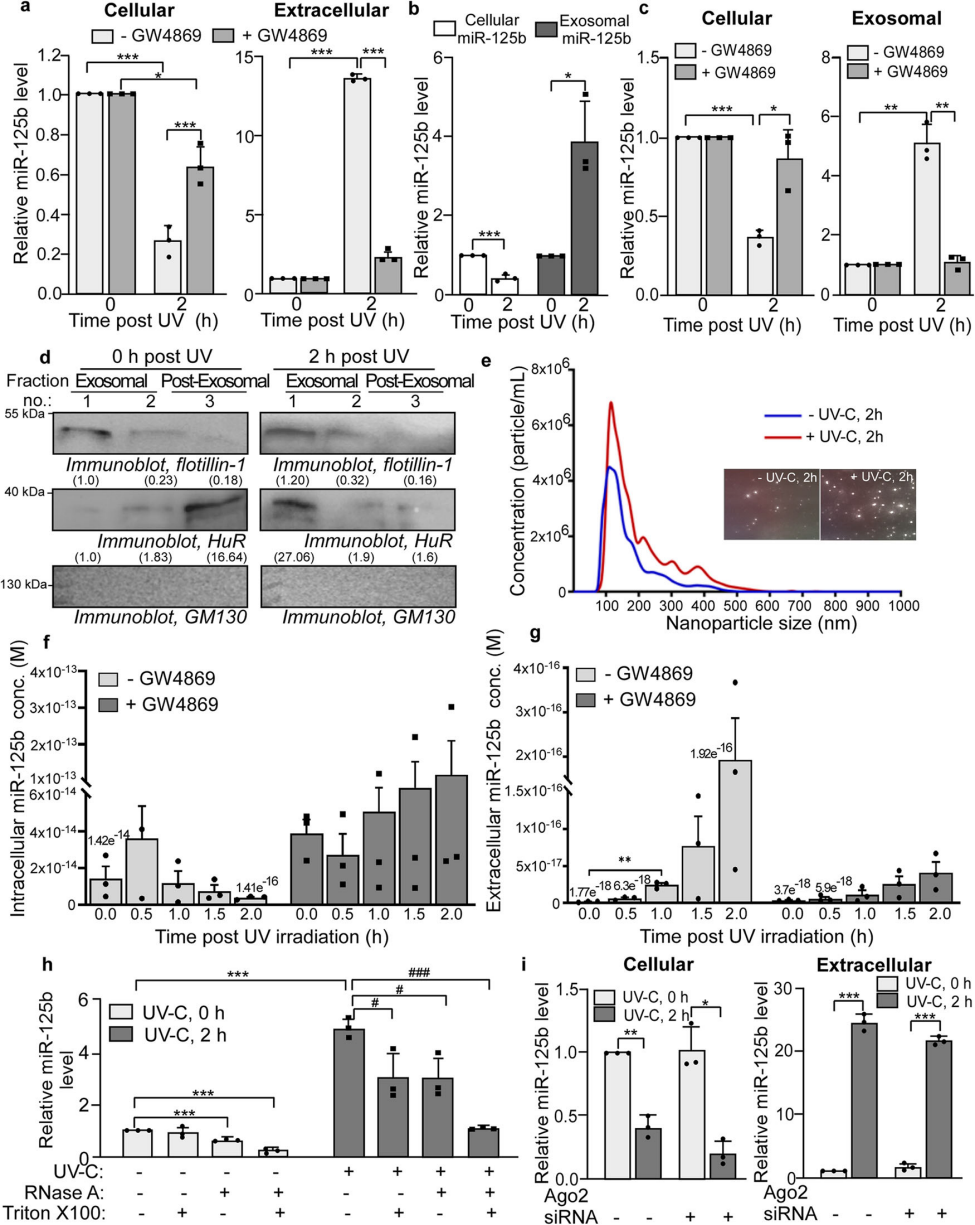
UV irradiation induces the exosomal export of miR-125b. **a** Estimation of miR-125b from MCF7 cells (left), untreated or treated with exosome inhibitor GW4869, and from cell supernatant (right), 0–2 h post-UVC irradiation, by qRT–PCR. Cellular miR-125b levels were normalized to U6B RNA levels and represented as fold change from 0 h UVC treatment set. Mean ± s.d. from three independent experiments are represented. **b** Estimation of cellular and exosomal miR-125b from MCF7 cells exposed to UVC irradiation and collected at indicated time points post-irradiation, by qRT–PCR. Cellular and exosomal miR-125b levels were normalized to U6B RNA levels and represented as fold change from respective RNAs at 0 h time point. Mean ± s.d. from three independent experiments are represented. **c** Estimation of cellular and exosomal miR-125b from MCF7 cells, untreated and treated with GW4869 and exposed to UVC irradiation, by qRT–PCR. Cellular and exosomal miR-125b levels were normalized to U6B RNA levels and represented as fold change from respective RNAs at 0 h time point. Mean ± s.d. from three independent experiments are represented. **d** Immunoblots of pooled exosomal fractions collected from the supernatant of MCF7 cells exposed to UVC and probed with flotillin-1, HuR, and GM130 antibodies. Fold changes in band intensities of flotillin-1 and HuR, normalized to band intensities from fraction no. 1, are mentioned below the respective bands. **e** Nanoparticle tracking analysis of extracellular vesicles from cells either exposed to or unexposed to UVC and collected 2 h post-UVC exposure. The data represents mean concentrations of nanoparticles plotted against particle size, obtained from five videos each from two independent experiments. The inset contains representative scene grabs from videos of each sample. **f** Absolute quantification of miR-125b concentration in MCF7 cells exposed to UVC, and either treated or untreated with GW4869, collected at indicated time points post-UVC exposure. miR-125b absolute concentrations were determined by qRT-PCR and interpolation of *C*_T_ values to a standard curve obtained for miR-125b. Mean ± s.d. from three independent experiments are represented. **g** Absolute quantification of miR-125b concentration in the supernatant of MCF7 cells exposed to UVC, and either treated or untreated with GW4869, collected at indicated time points post-UVC exposure. miR-125b absolute concentrations were determined by qRT-PCR and interpolation of *C*_T_ values to a standard curve obtained for miR-125b. Mean ± s.d. from three independent experiments are represented. **h** Estimation of miR-125b from exosomes collected from supernatant of UVC irradiated or non-irradiated MCF7 cells and either treated or not treated with RNase A in the presence or absence of Triton X-100. miR-125b levels were normalized to U6B RNA levels and represented as fold change compared to that from UVC non-irradiated cells, untreated with RNase A and Triton X-100. Mean ± s.d. from three independent experiments are represented. **i** Estimation of cellular and extra-cellular miR-125b from MCF7 cells transfected with Ago2 siRNA or control siRNA for 72 h, and then exposed to UVC irradiation and collected at indicated time points post-irradiation, by qRT–PCR. Cellular and extra-cellular miR-125b levels were normalized to U6B RNA levels and represented as fold change from control siRNA-transfected cells at 0 h time point. Mean ± s.d. from three independent experiments are represented. * # signifies *P* value ≤ 0.05, ** ## signifies *P* value ≤ 0.01 and *** ### signifies *P* value ≤ 0.005 (paired two-tailed *t*-test) in all panels.

The major EV species reported to be involved in the extracellular export of miRNAs are exosomes^23^. To investigate the possibility of exosome-mediated export of miR-125b post-UV-irradiation, cells were treated with the neutral sphingomyelinase II (nSMase) inhibitor GW4869 which prevents exosome biogenesis and secretion. GW4869 treatment prevented extracellular export of miR-125b significantly at 2 h post UV exposure and caused a concomitant increase in the cellular miR-125b level compared to DMSO-treated control (Fig. 2a). Exosomal fractions were isolated from the MCF-7 cell supernatant using size-exclusion chromatography, and identified by western blotting for the exosome marker flotillin-1 (Supplementary Fig. 2). A significantly higher amount of miR-125b was found in the pooled exosome fractions from cell supernatants collected 2 h after UV exposure, compared to 0 h controls (Fig. 2b). miR-125b level in the exosomal fractions was significantly reduced on treatment of cells with GW4869, with a concomitant increase in cellular miR-125b level, demonstrating that miR-125b is exported via exosomes (Fig. 2c). Flotillin-1 level in exosomal fractions obtained from cell supernatants post UV exposure was ~20% higher compared to that from UV-untreated cells, suggesting that UV irradiation may cause greater release of exosomes (Fig. 2d). Nanoparticle tracking analysis showed significant enhancement of the release of exosomes (~40–200 nm vesicles, with a peak diameter of 150 nm) from UV-irradiated cells compared to cells not exposed to UV radiation (Fig. 2e). In order to perform absolute quantification of miR-125b levels in cells and in exosomes, we generated a standard curve of miR-125b concentrations against average *C*_T_ values by quantitative PCR (Supplementary Fig. 3). We used the miR-125b standard curve to quantify the change in absolute concentration of miR-125b in cells and in extracellular medium in response to UV irradiation. The absolute concentration of miR-125b reduced from 1.42 × 10^−14^ to 1.41 × 10^−16^ M in cells 2 h post UV irradiation, whereas it increased from 1.77 × 10^−18^ to 1.92 × 10^−16^ M in the extracellular medium during the same time period (Fig. 2f and g, left bars). The ~100-fold reduction of miR-125b in cells and a corresponding increase in the extracellular medium demonstrated that extracellular export of miR-125b constituted the major mechanism for the reduction of miR-125b level in cells. Treatment with the exosome inhibitor GW4869 enhanced the absolute cellular level of miR-125b and prevented the increase in the extracellular level of miR-125b post UV irradiation (Fig. 2f and g, right bars).

Interestingly, the RBP HuR, which is a known target of miR-125b, has been reported to be involved in the exosomal export of miR-122^22^. HuR was found to be present mostly in the post-exosomal fraction in UV-untreated cells, but shifted to the exosomal fraction in UV-treated cells (Fig. 2d and Supplementary Fig. 2). Treatment of cells with the exosome inhibitor GW4869 caused the removal of flotillin-1 from the exosomal fractions, together with a strong reduction in HuR, suggesting that HuR is also released via exosomes in response to UV irradiation (Supplementary Fig. 4). Finally, we determined whether miR-125b was a bona fide cargo of exosomes and was not being secreted along with exosomes in response to UV-induced cell damage. Exosomal fractions isolated from supernatants of cells at 0 and 2 h post UV irradiation were treated with RNase A in the absence or presence of non-ionic detergent Triton X100. RNase A and Triton X100 individually caused some decrease in the exosomal miR-125b level, but RNase A in presence of Triton X100 caused a drastic decrease in the miR-125b level, suggesting that miR-125b was encapsulated in exosomes and detergent-mediated disruption of the exosomes exposed it to RNase A-mediated degradation (Fig. 2h). As miRNAs have also been reported to be exported in exosome independent and Ago2-associated manner^24^, we investigated whether the siRNA-mediated depletion of Ago2 affected the export of miR-125b in response to UV irradiation. Knockdown of Ago2 (Supplementary Fig. 5) did not affect the reduction of cellular miR-125b level 2 h post UV irradiation and also did not significantly affect the increase in extracellular miR-125b level during the same time period (Fig. 2i). This suggested that the extracellular export of miR-125b in response to UV irradiation is not majorly Ago2-dependent. In order to determine whether the exosomal export of miR-125b was specific, we also investigated whether miR-21 was exosomally exported in response to UV. The cellular level of miR-21 in UV-irradiated cells did not show any significant change in response to GW4869 treatment (Supplementary Fig. 6a). There was an increase in extracellular miR-21 level upon UV irradiation, but GW4869 treatment did not inhibit this increase (Supplementary Fig. 6b). Instead there was an increase in extracellular miR-21 levels upon GW4869 treatment, suggesting that miR-21 might be exported by a different mechanism which is enhanced upon inhibition of the exosomal pathway. Together, these data demonstrated that miR-125b is specifically exported via exosomes, and exosome-mediated export of miR-125b post 2 h of UV irradiation caused the reduction in miR-125b level as an initial response to UV exposure.

### HuR mediates the exosomal export of miR-125b in response to UV-C radiation

**Fig. 3:**
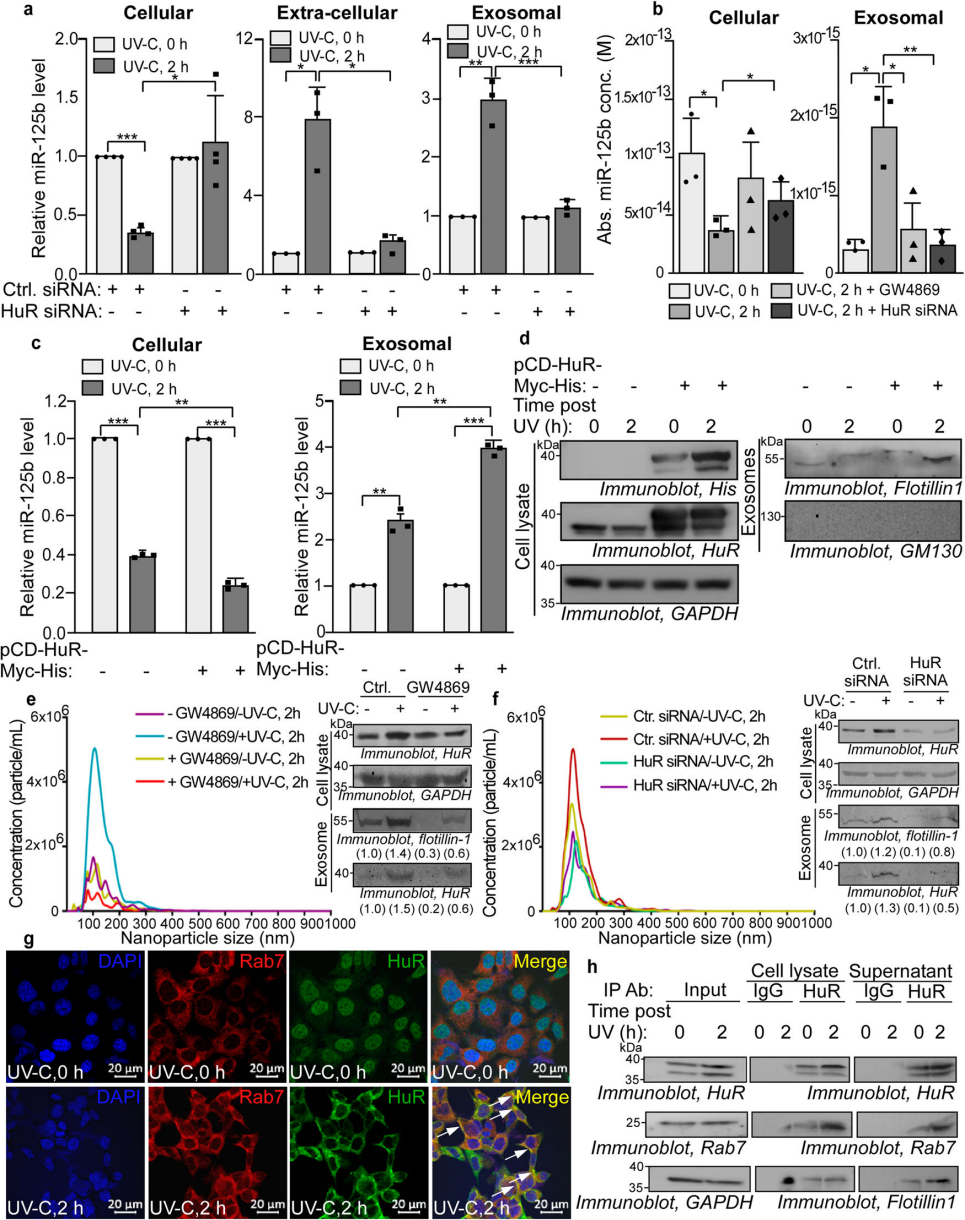
HuR mediates the exosomal export of miR-125b in response to UVC radiation. **a** Estimation of cellular (left), extra-cellular (middle) and exosomal (right) miR-125b by qRT–PCR from MCF7 cells transfected with control or HuR siRNA for 24 h and UVC irradiated. miR-125b levels were normalized to U6B RNA levels and represented as fold change from 0 h UVC irradiated set. Mean ± s.d. from four independent experiments for cellular miR-125b and three independent experiments for extra-cellular and exosomal miR-125b respectively are represented. **b** Absolute quantification of cellular and exosomal miR-125b concentration from MCF7 cells exposed to UVC, and either untreated or treated with GW4869 or transfected with HuR siRNA, collected at indicated time points post-UVC exposure. miR-125b absolute concentrations were determined by qRT-PCR and interpolation of *C*_T_ values to a standard curve obtained for miR-125b. Mean ± s.d. from three independent experiments are represented. **c** Estimation of cellular and exosomal miR-125b by qRT–PCR from MCF7 cells transfected with empty vector or myc-His-tagged HuR expression vector and UVC irradiated. miR-125b levels were normalized to U6B RNA levels and represented as fold change from 0 h UVC irradiated set. Mean ± s.d. from three independent experiments are represented. **d** Immunoblots of whole cell lysates and exosomal fractions collected from conditioned media of MCF7 cells transfected with HuR overexpression construct post 0 and 2 h of UVC irradiation probed with anti-His tag, HuR, GAPDH, Flotillin-1, and GM130 antibodies. **e** Nanoparticle tracking analysis of extracellular vesicles from cells either treated with or untreated with GW4869 and exposed to or unexposed to UVC, collected 2 h post-UVC exposure. The data represents mean concentrations of nanoparticles plotted against particle size, obtained from five videos each from two independent experiments. Immunoblots of cytoplasmic lysates and exosomal fractions collected from supernatant of MCF7 cells either treated with or untreated with GW4869 and exposed to or unexposed to UVC collected 2 h post-UVC exposure, probed with HuR, GAPDH, and Flotillin-1 antibodies (right). Fold changes in band intensities of flotillin-1 and HuR, normalized to band intensities from UV untreated control, are mentioned below the respective bands. **f** Nanoparticle tracking analysis of extracellular vesicles from cells transfected with either control siRNA or HuR siRNA and exposed to or unexposed to UVC collected 2 h post-UVC exposure. The data represents mean concentrations of nanoparticles plotted against particle size, obtained from five videos each from two independent experiments. Immunoblots of cytoplasmic lysates and exosomal fractions collected from supernatant of MCF7 cells transfected with either control siRNA or HuR siRNA and exposed to or unexposed to UVC collected 2 h post-UVC exposure, probed with HuR, GAPDH and Flotillin-1 antibodies (right). Fold changes in band intensities of flotillin-1 and HuR, normalized to band intensities from UV untreated, control siRNA-transfected cells, are mentioned below the respective bands. **g** Immunofluorescence of MCF7 cells exposed to UVC irradiation and collected at 0 and 2 h post-UVC exposure using Rab7 and HuR antibodies and anti-rabbit AlexaFluor 568-conjugated secondary antibody and anti-mouse FITC-conjugated secondary antibody respectively, at ×60 magnification. Nuclei were stained using DAPI staining. Co-localization of endogenous Rab7 (red) and HuR (green) was observed by merging Rab7 and HuR immunofluorescence images. **h** Immunoblots of input lysates and immunoprecipitates of HuR and normal IgG from lysates and supernatant of cells exposed to UVC irradiation and collected 2 h post-UVC exposure. Immunoprecipitates were probed with HuR, Rab7, Flotillin-1, and GAPDH antibodies. * Signifies *P* value ≤ 0.05, ** signifies *P* value ≤ 0.01 and *** signifies *P* value ≤ 0.005 (paired two-tailed *t*-test) in all panels.

As HuR was found to be enriched, together with miR-125b, in the exosomal fractions isolated from supernatants of cells exposed to UV we investigated whether HuR played a role in the exosomal export of miR-125b. siRNA-mediated knockdown of HuR caused an increase in intracellular miR-125b level in cells collected 2 h post UV exposure, with a concomitant decrease in extracellular miR-125b level in the cell supernatant (Fig. 3a, left and middle panels). HuR depletion also caused a significant decrease in miR-125b level in the exosomal fractions isolated from UV-irradiated cells (Fig. 3a, right panel). HuR was efficiently knocked down by siRNA transfection in both UV-treated and untreated cells (Supplementary Fig. 7). Absolute quantification of miR-125b also showed a significant increase in cellular miR-125b in HuR siRNA-transfected cells exposed to UV, similar to that in GW4869 treatment (Fig. 3b, left panel). Concomitantly, HuR knockdown significantly reduced the absolute miR-125b level in exosomes from UV-exposed cells, similar to the effect of GW4869 (Fig. 3b, right panel). HuR siRNA transfection did not significantly change the cellular level of miR-21 in UV-irradiated cells and did not reduce the exosomal miR-21 level in cells exposed to UV, suggesting that HuR did not facilitate the export of miR-21 (Supplementary Fig. 8). Conversely, over-expression of HuR from a eukaryotic expression vector showed a significant decrease in cellular miR-125b and increase in miR-125b level in exosomes from UV-treated cells in comparison to cells not over-expressing HuR (Fig. 3c). HuR overexpression also enhanced the level of flotillin-1 in the exosomal fractions in UV-treated cells, suggesting increased generation of exosomes (Fig. 3d, right panel). In order to investigate whether HuR affected the generation of exosomes from UV-irradiated cells, we did nanoparticle tracking analysis of exosomes from cells treated with GW4869 or transfected with HuR siRNA. GW4869 treatment abrogated the enhanced generation of exosomes from UV-exposed cells together with the reduction of flotillin-1 and HuR levels in the exosomes (Fig. 3e). Similarly, HuR knockdown inhibited the enhanced generation of exosomes from UV-irradiated cells and showed a concomitant decrease in exosomal flotillin-1 and HuR levels (Fig. 3f). Together, these observations showed that the RNA-binding protein HuR facilitated the exosomal export of miR-125b and enhanced the generation of exosomes from UV-irradiated cells.

HuR has been reported to cause the exosomal export of another miRNA, miR-122, by direct interaction with the miRNA^22^. We have previously observed that HuR does not interact with a miR-125b probe in an RNA EMSA^9^. Immunoprecipitation of HuR from cells and cell supernatants exposed to or unexposed to UV did not show the interaction between miR-125b and HuR (Supplementary Fig. 9). This suggested that the facilitation of miR-125b exosomal export by HuR is not by direct interaction with the miRNA, unlike the case of miR-122. Therefore we investigated whether HuR interacted with the exosomal trafficking machinery. Immunofluorescence studies showed the efficient nuclear-cytoplasmic translocation of HuR at 2 h post UV exposure, and co-localization with Rab7, a GTPase associated with exosome precursor endosomes and regulating exosome release (Fig. 3g). Rab7 was also co-immunoprecipitated with HuR, both from cell lysate and cell supernatant, with the association enhanced by UV exposure (Fig. 3h). HuR was also found to be associated with the exosomal component flotillin 1 both in cell lysate and extracellular medium, but the association was enhanced only in supernatant on UV exposure (Fig. 3h). Flotillin 1 is reported to be involved in regulating the composition of exosomes and not in the release of exosomes. Together, these observations indicated that HuR mediated the exosomal export of miR-125b by interacting with the exosomal trafficking machinery and enhancing exosome generation, thereby determining the decrease in cellular miR-125b level as an initial response to UV irradiation.

### p53-mediated transcriptional upregulation determines the delayed increase in miR-125b level

**Fig. 4:**
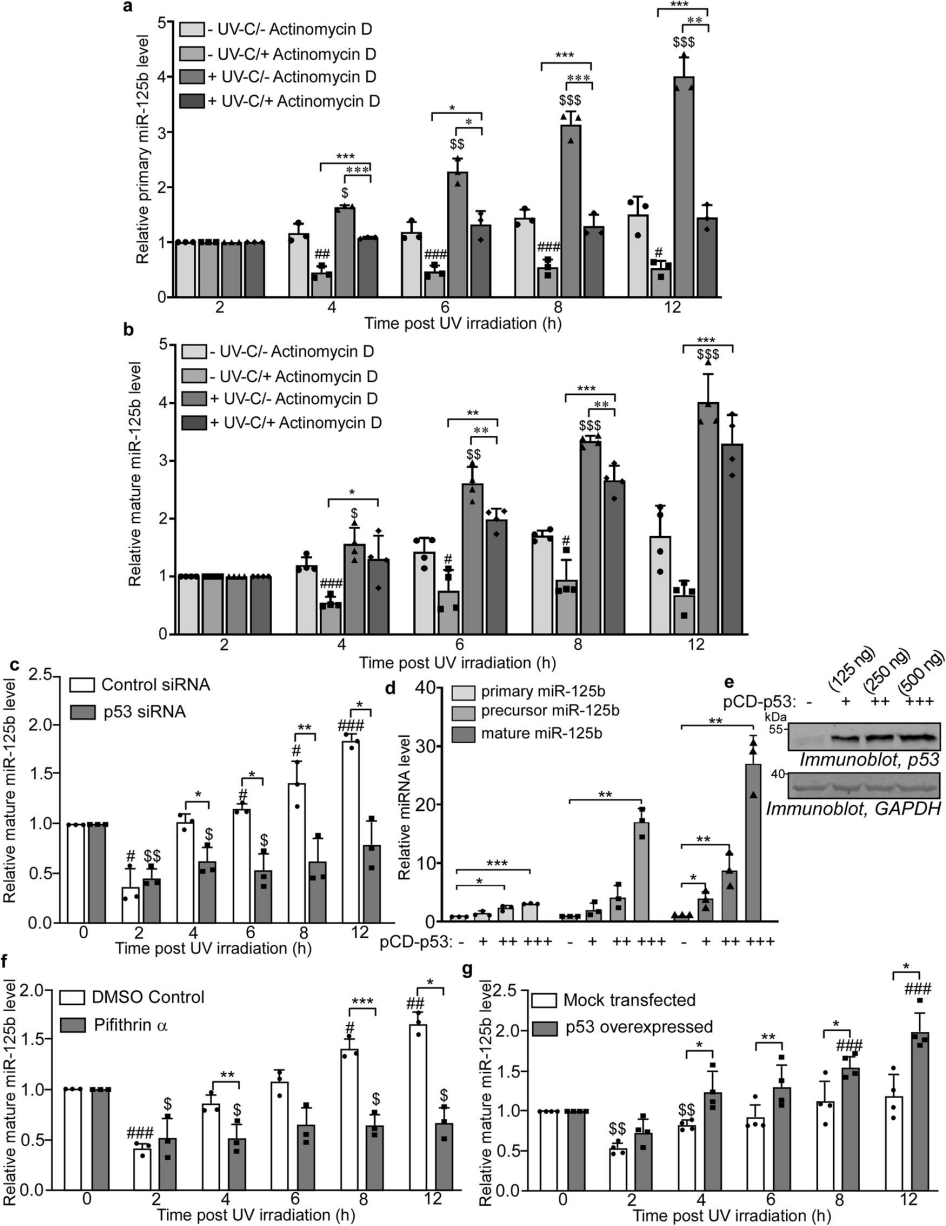
p53-mediated transcriptional upregulation determines the delayed increase in miR-125b level. **a** MCF7 cells subjected to four different treatments of UVC not exposed, UVC not exposed with actinomycin D, UVC exposed and UV exposed with actinomycin D respectively, were collected at the indicated time points, total RNA was isolated followed by qRT-PCR using primary miR-125b and U6B primers. Primary miR-125b levels were normalized to U6B RNA levels. Mean ± s.d. from three independent experiments are represented. **b** Estimation of mature miR-125b from MCF7 treated as in (**a**) above. Mean ± s.d. from four independent experiments are represented. **c** Estimation of mature miR-125b by qRT-PCR from MCF7 cells transfected with either control or p53 siRNA for 48 h, UVC irradiated and collected at indicated time points from 0 to 12 h post-UVC exposure. miR-125b levels were normalized to U6B RNA levels and represented as fold change from 0 h UVC irradiated set. Mean ± s.d. from three independent experiments are represented. **d** Estimation of primary, precursor, and mature miR-125b by qRT-PCR from MCF7 cells transfected with increasing concentrations (125, 250, and 500 ng) of p53 overexpression construct. Primary, precursor, and mature miR-125b levels were normalized to U6B RNA levels and represented a fold increase from the mock-transfected set. Mean ± s.d. from three independent experiments are represented. **e** Immunoblots of whole cell lysates of MCF7 cells transfected with three increasing concentrations (125, 250, and 500 ng) p53 overexpression construct, probed with p53 and GAPDH antibodies. **f** Estimation of mature miR-125b by qRT-PCR from MCF7 cells treated with pifithrin-α for 5 h, UVC irradiated and collected at indicated time points from 0 to 12 h post-UVC exposure. miR-125b levels were normalized to U6B RNA levels and represented as fold increase or decrease from 0 h UVC irradiated set. Mean ± s.d. from three independent experiments are represented. **g** MDA-MB-231 cells were transfected with either empty vector or p53 overexpression vector, UVC irradiated, and collected from 0 to 12 h post-UVC exposure. Total RNA was isolated followed by qRT-PCR using miR-125b and U6B-specific primers. miR-125b levels were normalized to U6B RNA levels and represented as a fold increase from 0 h UVC irradiated set. Mean ± s.d. from four independent experiments are represented. *, *, #, $ signifies *P* value ≤ 0.05, **, **, ##, $$ signifies *P* value ≤ 0.01 and ***, ***, ###, $$$ signifies *P* value ≤ 0.005 (paired two-tailed *t*-test) from respective controls.

The significant increase in miR-125b level from 2 to 12 h post UV irradiation may be due to transcriptional upregulation or enhanced processing or both. To investigate the mechanism of this increase, we treated both UV-exposed and unexposed cells with the transcription inhibitor Actinomycin D and determined primary and mature miR-125b levels. UV-exposed cells showed an approximately 4-fold increase in primary miR-125b level from 2 to 12 h post UV irradiation, which was nearly completely abrogated on Actinomycin D treatment (Fig. 4a), suggesting a transcriptional upregulation of miR-125b during this time period. Mature miR-125b level also showed a 4-fold increase from 2 to 12 h post UV irradiation and Actinomycin D treatment caused a decrease in the mature miR-125b level (Fig. 4b). However, the extent of decrease was substantially less compared to that of primary miR-125b for all the time points. This indicated that together with transcriptional upregulation, UV irradiation might also enhance the processing of primary to mature miR-125b, with both processes contributing to the delayed increase in miR-125b post-UV exposure.

As the increase in miR-125b level from 2 h post UV irradiation temporally coincides with the increase in p53 protein level (Fig. 1a), we investigated the role of p53 in the enhancement of cellular miR-125b level. UV irradiation of cells transfected with p53 siRNA showed a complete abrogation of the UV-induced increase in miR-125b, in comparison to UV-exposed cells transfected with a control siRNA (Fig. 4c). p53 siRNA showed an efficient knockdown of p53 protein at all time points (Supplementary Fig. 10). Conversely, overexpression of p53 caused a dose-dependent increase in primary, precursor, and mature miR-125b levels, even in absence of UV treatment, with a significantly greater increase in precursor and mature miR-125b levels (Fig. 4d and e). Together, these data demonstrated the effect of p53 in enhancing miR-125b expression post 2 h of UV irradiation, potentially by enhancing both the transcription and processing of miR-125b. Treatment of cells with pifithrin α, an inhibitor of p53 transcriptional activity, resulted in the abrogation of the UV-induced increase in miR-125b level (Fig. 4f). We also checked the expression dynamics of miR-125b in MDA-MB-231 cells, a breast carcinoma cell line with a transcriptionally inactive mutant (R280K) of p53. miR-125b showed a much-reduced enhancement in MDA-MB-231 cells from 4 to 12 h post UV irradiation compared to MCF7 cells. However exogenous expression of WT p53 caused a significant increase in miR-125b levels in MDA-MB-231 cells from 4 to 12 h post UV irradiation, confirming the transcriptional role of p53 in miR-125b expression post 2 h of UV irradiation (Fig. 4g).

### miR-125b is a transcriptional target of p53

**Fig. 5:**
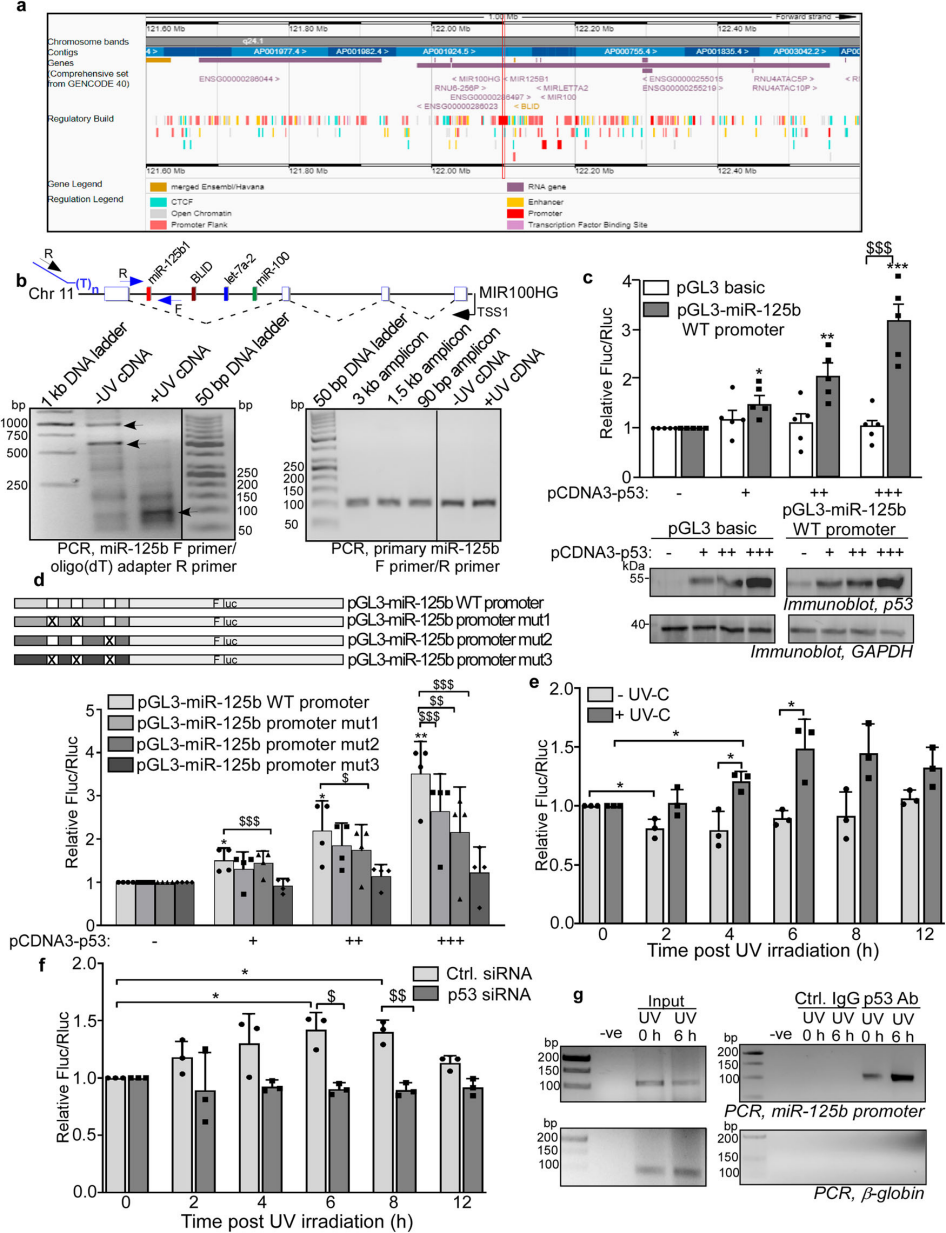
miR-125b is a transcriptional target of p53. **a** ENSEMBL graphic for the MIR100HG lincRNA gene in Hsa Chr 11, with the putative promoter upstream of the miR-125b-1 encoding sequence in the regulatory build indicated by a red box. **b** Schematic diagram of the MIR100HG gene. The exonic sequences for the lincRNA are indicated by blue boxes and the four intronic genes are indicated. The F and R primers used for PCR are indicated by black (oligo(dT) adapter reverse primer) and blue (miR-125b-1 forward and reverse primers) arrows. PCR was done from cDNAs generated from RNA collected from cells unexposed or exposed to UVC. Reverse transcription was done using an oligo(dT)-adapter primer. PCR was done with pri-miR-125b F and adapter-specific R primers (left panel) and pri-miR-125b F and R primers (right panel). **c** Luciferase assay of lysates from MCF7 cells transfected with pGL3 basic vector or pGL3-miR-125b-wild type (WT) promoter vector along with pCMV-Rluc in presence of three increasing concentrations (125, 250, and 500 ng) of p53 overexpression construct. Fluc values are normalized to Rluc values as transfection control. Mean ± s.d. from five independent experiments are represented. Bottom panel represents immunoblots of the lysates probed with p53 and GAPDH antibodies. (**d**) Luciferase assay of lysates from MCF7 cells transfected with pGL3-miR-125b-(WT) promoter vector and three different p53-binding site mutant constructs of miR-125b promoter along with pCMV-Rluc in presence of increasing concentrations of p53 overexpression construct. Fluc values are normalized to Rluc values as transfection control. Mean ± s.d. from four independent experiments are represented. **e** Luciferase assay of lysates from MCF7 cells transfected with pGL3-miR-125b-(WT) promoter vector along with pCMV-Rluc and exposed or not exposed to UVC irradiation and collected at indicated time points post UVC exposure. Fluc values are normalized to Rluc values as transfection control. Mean ± s.d. from three independent experiments are represented. **f** Luciferase assay of lysates from MCF7 cells transfected with pGL3-miR-125b-(WT) promoter vector along with pCMV-Rluc in presence of either control or p53 siRNA. Cells were UVC exposed and collected at indicated time points post-UVC irradiation. Fluc values are normalized to Rluc values as transfection control. Mean ± s.d. from three independent experiments are represented. **g** Chromatin immunoprecipitation of UVC untreated and treated cells using normal IgG and p53 antibody followed by PCR amplification using primers specific to miR-125b promoter and human Β-globin gene. * and $ signifies *P* value ≤ 0.05, ** and $$ signifies *P* value ≤ 0.01, *** and $$$ signifies *P* value ≤ 0.005 (paired two-tailed *t*-test) from respective controls.

p53 is known to activate the transcription of a number of miRNAs; however, its function as a transcription inducer of miR-125b is unknown. We, therefore, investigated whether miR-125b is a direct transcriptional target of p53. Primary miR-125b is transcribed as part of the *miR-100/let-7a-2/miR-125b-1* and *miR-99a/let-7c/miR-125b-2* tricistrons from Chr 11 and 21, respectively^25^. MIR100HG and MIR99AHG (LINC00478) represent the lincRNA host genes of the tricistrons^26^. The pri-miR-125b is encoded as part of the intronic sequence of these lincRNA genes and processed to generate the mature miR-125b. We looked at the predicted transcription factor binding sites in the promoters and enhancers of these lincRNA genes and did not find any p53 binding sites. Interestingly, we found that the immediate upstream region of the miR-125b-1-encoding sequence in MIR100HG is predicted as a promoter region in the regulatory build in ENSEMBL (Fig. 5a). The 525 bp upstream region of the miR-125b-1-encoding sequence (the region between miR-125b-1 and the upstream intronic gene BLID) located at 11q24.1 was analyzed for the presence of p53-binding sites using the TFbind program (Supplementary Data 1). Three putative p53-binding sites were identified, with the site closest to the miR-125b sequence showing the highest conservation in mammalian species (Supplementary Fig 11a). This suggested that a cryptic promoter might be located upstream of the miR-125b-1 region of the MIR100HG gene, driving the transcription of pri-miR-125b. The MIR100HG gene is known to generate multiple transcripts by alternative transcription and splicing involving its multiple exons and introns. In order to investigate this, we performed reverse transcription of RNA isolated from 6 h UV-treated and untreated cells, using an oligo(dT) primer with an overhanging adapter sequence (Fig. 5b). PCR with a forward primer corresponding to pri-miR-125b-1 and reverse primer corresponding to the adapter sequence resulted in the generation of multiple PCR products from the UV-untreated cDNA, with ~3 and ~1.5 kb bands predominating (Fig. 5b, left panel). This corresponded to the multiple transcripts generated by alternative splicing of the MIR100HG RNA. Remarkably, PCR using the same primer set with the cDNA from UV-treated cells showed the near disappearance of the higher-sized PCR products and the generation of a predominant product of ~90 bp, which corresponded in size with pri-miR-125b-1 (Fig. 5b, left panel). When we isolated the DNA from the ~3, ~1.5 kb, and ~90 bp PCR amplicons and performed PCR using forward and reverse primers corresponding to pri-miR-125b-1, all three amplicons generated the 88 bp PCR product corresponding to miR-125b, as was also observed from PCR with cDNA from UV-treated and untreated cells using the same primer set (Fig. 5b, right panel). Subsequent sequencing confirmed the ~3 kb and ~90 bp PCR products as originating from MIR100HG and pri-miR-125b-1, respectively. These observations showed the presence of a UV-induced cryptic promoter upstream of miR-125b-1 in the MIR100HG gene which caused the transcription of pri-miR-125b RNA upon UV exposure.

The DNA sequence corresponding to this region proposed to contain the putative cryptic miR-125b promoter was inserted upstream of a firefly luciferase reporter gene in the pGL3-basic vector. The pGL3-miR-125b WT promoter construct when transfected into MCF-7 cells showed a dose-dependent increase in firefly luciferase expression in presence of increasing concentrations of p53, which was not observed in case of the pGL3-basic vector (Fig. 5c). Systematic mutation of the three putative p53-binding sites in the putative miR-125b promoter resulted in decrease of luciferase expression in presence of p53 overexpression, with a near complete reduction of luciferase activity to basal level on mutation of all the three sites (Fig. 5d and Supplementary Fig. 11b). UV irradiation, which causes a pulsatile expression of p53, increased miR-125b WT promoter activity from 2 to 6 h post UV exposure with a subsequent decrease, corresponding closely with the previously observed expression pattern of p53 (Fig. 5e). The enhancement of miR-125b WT promoter activity in UV-irradiated cells was completely abrogated on siRNA-mediated knockdown of p53, demonstrating the role of p53 in inducing the miR-125b promoter activity (Fig. 5f). Finally, we performed chromatin immunoprecipitation (ChIP) assay to determine the direct interaction between p53 and the miR-125b promoter region DNA. ChIP assay using p53 antibody showed the specific association of the miR-125b promoter region with p53, which was further enhanced in UV-irradiated cells (Fig. 5g). A region of similar length from the promoter region of the human *b-globin* gene, which lacks p53 binding sites, did not show any amplification on ChIP. Together, these observations established miR-125b as a *bonafide* transcriptional target of p53 and validated the role of p53 in the transcriptional upregulation of miR-125b expression from a cryptic promoter as a consequence of UV-C irradiation.

### p53 enhances the processing of miR-125b

**Fig. 6:**
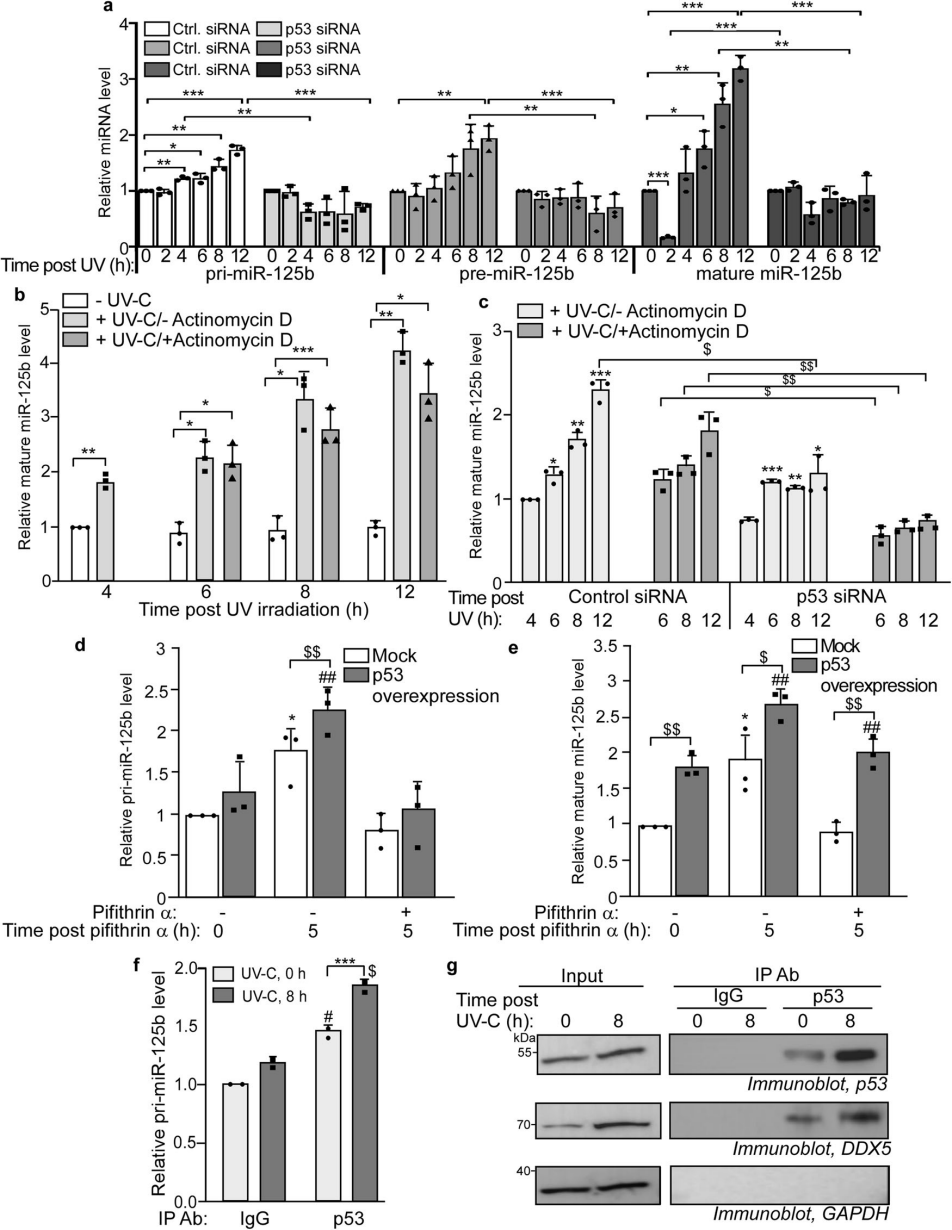
p53 enhances the processing of miR-125b. **a** Estimation of primary, precursor, and mature miR-125b by qRT-PCR from MCF7 cells transfected with control siRNA or p53 siRNA for 48 h, UVC-irradiated and collected at the indicated time points post-UVC irradiation. Primary, precursor, and mature miR-125b levels were normalized to U6B RNA levels and represented as a fold increase from 0 h control for every transfection set. Mean ± s.d. from three independent experiments are represented. **b** MCF7 cells which were either not UVC-irradiated or UVC-irradiated, and 4 h after UVC exposure cells were either treated or untreated with actinomycin D. Mature miR-125b was estimated by qRT-PCR from cells collected at the indicated time points post-UVC exposure and Actinomycin D treatment. Mature miR-125b levels were normalized to U6B RNA. miR-125b levels from non-UVC irradiated and UVC-irradiated cells were expressed as fold change from the miR-125b levels of respective controls at 4 h time point. Mean ± s.d. from three independent experiments are represented. **c** MCF7 cells, transfected with control or p53 siRNA 48 h prior to UVC exposure, were UVC irradiated and either treated or untreated with actinomycin D 4 h post-UVC irradiation. Mature miR-125b was estimated by qRT-PCR from treated cells collected at the indicated time points post-UVC exposure. Mature miR-125b levels were normalized to U6B RNA. Mean ± s.d. from three independent experiments are represented. **d** Estimation of primary miR-125b by qRT-PCR from MCF7 cells transfected with p53 overexpression construct and treated with pifithrin α, collected 0 and 5 h after pifithrin α treatment. Primary miR-125b levels were normalized to U6B RNA. Mean ± s.d. from three independent experiments are represented. **e** Mature miR-125b was estimated from cells treated similarly to **d** above. Mean ± s.d. from three independent experiments are represented. **f** RNA-immunoprecipitation (RIP) of lysates from MCF7 cells exposed to UVC irradiation and collected at 0 and 8 h post-UVC exposure using control IgG and p53 antibody, followed by estimation of pri-miR-125b by qRT-PCR from the RNA isolated from the immunoprecipitates. Pri-miR-125b levels were normalized to U6B RNA levels and represented as a fold increase or decrease from control IgG IP RNA from 0 h UVC irradiation set. Mean ± s.d. from two independent experiments are represented. **g** Immunoblots of input lysates and immunoprecipitates of p53 and normal IgG from cells irradiated with UV-C and collected 8 h post-UVC exposure. Immunoprecipitates were probed with p53, DDX5, and GAPDH antibodies. *, #, $ signifies *P* value ≤ 0.05, **, ##, $$ signifies *P* value ≤ 0.01, and ***, ###, $$$ signifies *P* value ≤ 0.005 (paired two-tailed *t*-test) from respective controls in all panels.

p53 is reported to enhance the processing of multiple miRNAs, especially in response to DNA damage^18^. Therefore we investigated whether p53 facilitated the processing of miR-125b. We first investigated the changes in primary (pri), precursor (pre), and mature miR-125b in UV-irradiated cells upon siRNA-mediated depletion of p53. Knockdown of p53 resulted in the reduction of pri-, pre- and mature miR-125b levels in UV-exposed cells in comparison to control siRNA-transfected cells exposed to UV (Fig. 6a). Overexpression of p53 had enhanced the expression of pri-miR-125b, pre-miR-125b, and mature miR-125b, but the enhancement was more pronounced in the case of pre-miR-125b and mature miR-125b (Fig. 4d), suggesting a role of p53 in the processing of miR-125b. In order to differentiate the effects of p53 on miR-125b transcription and processing, MCF-7 cells were UV exposed, and 4 h after UV exposure a set of cells were treated with Actinomycin D to stop transcription. Mature miR-125b levels were determined in all sets of cells at different time points post Actinomycin D treatment. Mature miR-125b levels increased in UV-irradiated cells, untreated with Actinomycin D (Fig. 6b). Actinomycin D treatment reduced the rate of increase of miR-125b in UV-irradiated cells, but miR-125b levels at the time point post-Actinomycin D treatment was still significantly higher than in cells unexposed to UV (Fig. 6b). This suggested that the increase in miR-125b levels, after transcription inhibition, may be due to enhanced processing of primary miR-125b (pri-miR-125b) induced by UV. In order to investigate the role of p53 in the processing of pri-miR-125b, cells in which p53 has been knocked down were UV irradiated and treated or untreated with Actinomycin D, 4 h after UV irradiation. Actinomycin D would cause inhibition of transcription of pri-miR-125b, and the subsequent increase of mature miR-125b can be attributed to pri-miR-125b processing. miR-125b levels increased both in the presence and absence of Actinomycin D treatment, as observed before, in control siRNA transfected cells (Fig. 6c). However, there was no significant increase in miR-125b in p53 knockdown cells without Actinomycin D treatment and complete abrogation upon Actinomycin D treatment, indicating that p53 depletion prevented the processing of existing primary miR-125b after transcription inhibition.

In order to further investigate whether the transcriptional activity of p53 was indirectly responsible for the enhanced processing of miR-125b, cells overexpressing p53 were treated with pifithrin-α, a specific inhibitor of p53 transcriptional activity for 5 h. p53 overexpression caused significant upregulation of primary miR-125b level, while treatment with pifithrin-α abrogated the increase in primary miR-125b (Fig. 6d). p53 overexpression also caused significant upregulation in mature miR-125b level as expected. However, mature miR-125b level continued to be significantly higher in p53-overexpressing cells compared to mock-transfected cells upon pifithrin-α treatment, suggesting that inhibition of the transcriptional activity of p53 did not prevent the enhanced processing of miR-125b by p53 (Fig. 6e). This indicated that the enhanced processing of miR-125b mediated by p53 is independent of its transcriptional activity. Earlier reports have shown that p53 enhances the processing of several miRNAs by directly interacting with the Drosha processing complex through its association with DEAD-box RNA helicase p68 (DDX5) in response to DNA damage^18^. Immunoprecipitation of p53 from cells 8 h post UV irradiation showed a significantly higher association of p53 with primary miR-125b, suggesting a direct association of p53 with a processing complex containing miR-125b primary RNA (Fig. 6f). p53 was also co-immunoprecipitated with DDX5 and the association with DDX5 was enhanced in UV-irradiated cells (Fig. 6g). Together these observations indicated that p53 enhanced the processing of pri-miR-125b to mature miR-125b in response to DNA damage by directly associating with pri-miRNA processing complex, further contributing to the increase in cellular miR-125b level.

### The biphasic dynamics of miR-125b regulate the pulsatile expression of p53 and HuR in response to UV-irradiation

**Fig. 7:**
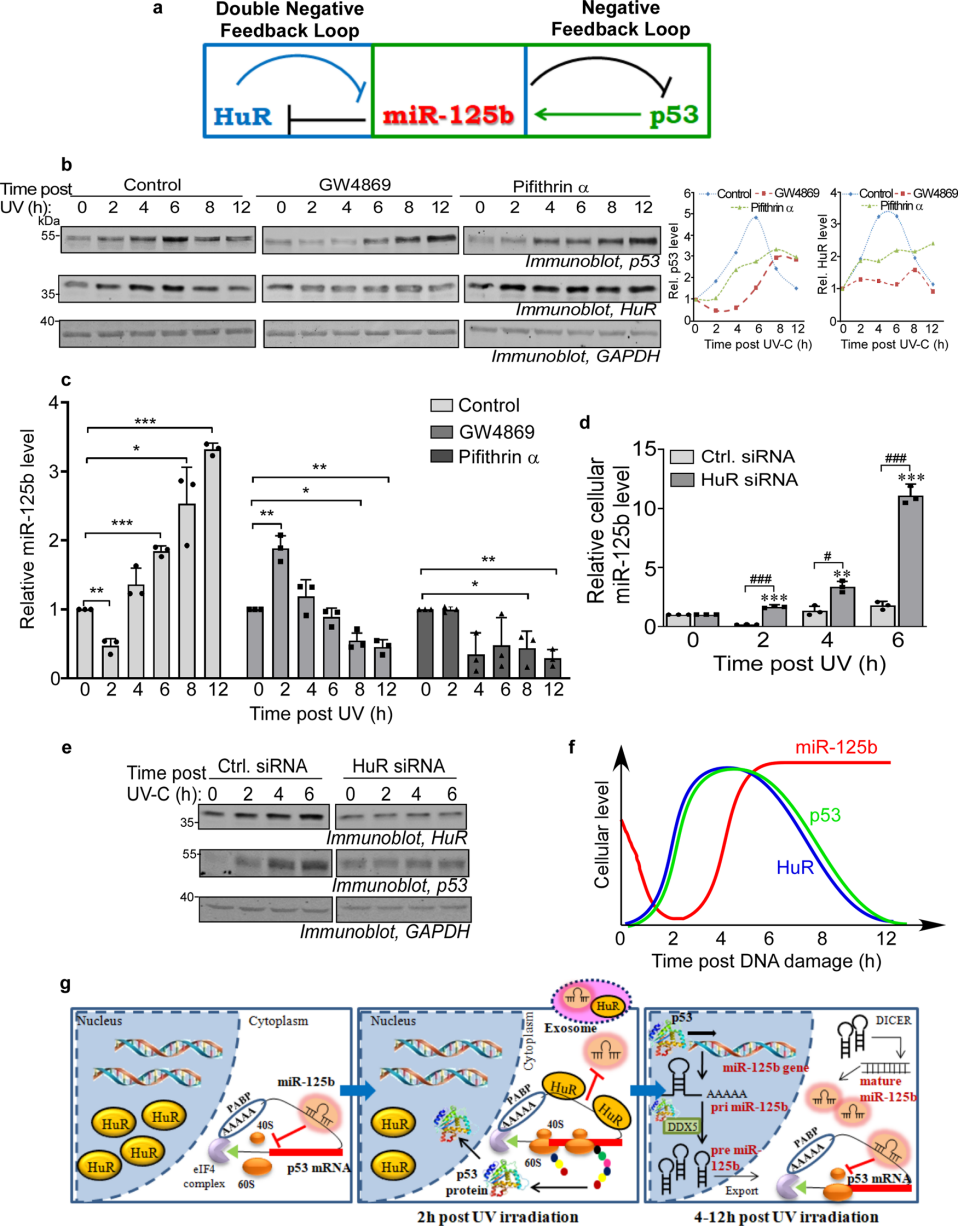
Biphasic dynamics of miR-125b regulates pulsatile expression of p53 and HuR post-UVC irradiation. **a** Overlapping double negative and negative feedback loops between HuR, miR-125b, and p53 mediated by exosomal export, transcriptional upregulation, miRNA processing and translation repression. **b** Representative immunoblots of cytoplasmic lysates from MCF7 cells exposed to UVC irradiation, and either untreated or treated with 20 µM GW4869 or 20 µM pifithrin α and collected at different time points post-UVC exposure, with p53, HuR, and GAPDH antibodies (left). Graphical representation of the change of p53 and HuR protein levels, normalized to GAPDH protein levels, over time post-UVC irradiation in the absence or presence of GW4869 or pifithrin α treatment (right). **c** qRT-PCR of total RNA isolated from UVC-irradiated MCF7 cells, either untreated or treated with 20 µM GW4869 or 20 µM pifithrin α, collected at indicated time points post-UVC exposure using miR-125b-specific primers. miR-125b RNA levels were normalized to U6B snRNA levels. Data represent fold change of normalized miR-125b RNA level in UVC-treated cells taking 0 h miR-125b RNA level for each treatment as 1. Mean ± s.d. from three independent experiments are represented. * signifies *P* value ≤ 0.05, ** signifies *P* value ≤ 0.01, and *** signifies *P* value ≤ 0.005 (paired two-tailed *t*-test) from respective 0 h controls. **d** Estimation of miR-125b from UVC-irradiated MCF7 cells, transfected with control siRNA or HuR siRNA, collected at indicated time points till 6 h post-UVC irradiation. miR-125b RNA levels were normalized to U6B snRNA levels. Mean ± s.d. from three independent experiments are represented. # Signifies *P* value ≤ 0.05 and  ### signifies *P* value ≤ 0.005 (paired two-tailed *t*-test) from Ctrl. siRNA transfected cells and ** signifies *P* value ≤ 0.01 and *** signifies *P* value ≤ 0.005 from 0 h control. **e** Immunoblots of lysates from UVC-irradiated MCF7 cells, transfected with control siRNA or HuR siRNA, collected at indicated time points till 6 h post-UVC irradiation with p53, HuR, and GAPDH antibodies. **f** Schematic representation of the pulses of p53 and HuR and the biphasic expression of miR-125b in cells in response to DNA damaging UVC radiation. **g** Molecular mechanisms consisting of exosomal export, transcriptional upregulation, and miRNA processing temporally regulating the biphasic expression dynamics of miR-125b in the 2–12 h post-exposure to UVC irradiation which contributes to the feedback loops generating the pulses of p53 and HuR.

Our observations suggest that miR-125b links together a double negative feedback loop with HuR and a negative feedback loop with p53, whereby the initial HuR-mediated export and delayed p53-mediated transcriptional upregulation and processing of miR-125b in turn regulates the pulsatile expression dynamics of p53 and HuR in response to UV irradiation (Fig. 7a). Therefore, we investigated the expression dynamics of p53 and HuR in UV-irradiated cells treated with GW4869 and pifithrin α, which inhibits the export and transcription of miR-125b, respectively. The pulsatile change of p53 and HuR levels is observed in control cells but GW4869 and pifithrin α treatment completely abrogate the pulsatile dynamics of p53 and HuR (Fig. 7b). Upon GW4869 treatment, the p53 level shows a delayed increase and finally attains a plateau. This is consistent with the retention of miR-125b in cells due to inhibition of exosomal export and resultant silencing of p53 expression, as supported by the increased miR-125b level in cells at 2 h post UV exposure (Fig. 7c). Pifithrin α treatment causes an earlier increase in p53 level which increases continuously and attains a plateau (Fig. 7b). This is also consistent with upregulation of p53 mRNA translation, as a result of reduced cellular level of miR-125b upon pifithrin α treatment (Fig. 7c). HuR, which is also subject to translation repression by miR-125b, shows a similar abrogation of its pulsatile dynamics upon treatment with GW4869 and pifithrin α (Fig. 7b). We have also investigated the effect of knockdown of HuR on the cellular p53 and miR-125b levels during the 6 h period post-UV-irradiation when HuR facilitates the export of miR-125b. The depletion of HuR results in the continuous increase of cellular miR-125b level, consistent with the inhibition of exosomal export (Fig. 7d). This is accompanied by the inhibition of the UV-induced increase of p53, reflecting the enhanced translation repression by the cellular miR-125b and loss of translation upregulation by HuR (Fig. 7e).

We have also investigated these phenomena in MCF10A cells, a non-tumorigenic but immortalized breast epithelial cell line that contains wild-type p53^27^. p53 and HuR levels show pulsatile changes in MCF10A cells upon UV-C irradiation, which is abrogated on treatment with GW4869 and pifithrin α (Supplementary Fig. 12a), similar to that observed in MCF7 cells. Also, miR-125b shows a similar biphasic pattern of expression upon UV irradiation of MCF10A cells, but not in absence of UV irradiation (Supplementary Fig. 12b). This biphasic expression pattern of miR-125b is not observed in the case of miR-21 (Supplementary Fig. 12c) and is also abrogated upon treatment with GW4869 and pifithrin α (Supplementary Fig. 12b), consistent with the observations in MCF7 cells. Together, these observations demonstrate that the UV-induced biphasic dynamics of miR-125b in tumorigenic and normal breast epithelial cells contribute to the pulsatile dynamics of both p53 and HuR by multiple intersecting feedback loops.

## Discussion

The spatio-temporal dynamics of miRNAs have been found to be an important determinant of gene expression in a variety of stress responses^15^. Systematic deletion of individual miRNAs has shown that many miRNAs are dispensable for normal development or viability under standard conditions but not when the organisms are subjected to stress^28–30^. Besides physiological and environmental changes, most pathological conditions, ranging from cancer to infectious diseases, causes alterations in miRNA levels, giving rise to specific “miRNA signatures” with important diagnostic and prognostic values^31,32^. Although the dynamic changes in miRNA expression in response to various physiological and pathological stress conditions is well known, the molecular mechanisms determining such changes have remained relatively unexplored. Here we have shown that regulatory mechanisms, comprising of exosomal export, transcriptional activation and enhanced processing, determine the cellular dynamics of miR-125b in response to genotoxic stress. Remarkably, these regulatory mechanisms are mediated by an RBP, HuR, and a transcription factor, p53, both of which are targets of miR-125b, giving rise to a complex regulatory network that contributes to pulsatile expressions patterns of p53, HuR, and miR-125b in response to DNA damage.

We have previously shown that a combination of miR-125b-mediated repression of p53 and HuR expression, TRIM-21-mediated degradation of HuR, and HuR-mediated upregulation of p53 mRNA translation constitutes overlapping feedforward loops that generate pulses of p53 and HuR proteins in response to UV irradiation^21^. In the course of these observations, we found that the miR-125b level itself shows a biphasic, pulse-like change, opposite in phase to HuR and p53. miRNAs have been reported to be strategically located in negative, positive, and double-negative feedback loops to regulate gene expression networks in stress responses^15^. The initial exosomal export and the subsequent transcriptional upregulation and enhanced processing of miR-125b, facilitated by HuR and p53, respectively, are temporally linked to generating feedback loops that determine the biphasic miR-125b dynamics and ultimately regulate the pulsatile expression of p53 and HuR (Fig. 7a and f).

The post-transcriptional regulation of p53 expression in response to UV irradiation involves the nuclear-cytoplasmic translocation of HuR as an immediate response resulting in the interaction of HuR with the 3’UTR of p53 mRNA and preventing the interaction of miR-125b, resulting in the upregulation of p53 mRNA translation^20^. Now we show that HuR simultaneously mediates the exosomal export of miR-125b, thereby reducing cellular miR-125b level, and further reducing the binding of miR-125b to the p53 mRNA (Fig. 7g). This results in the enhanced expression of p53 due to the reduction in miR-125b-mediated translation repression. Extracellular export of miRNAs is well documented and RBPs such as HuR and FMR1 have been shown to be involved in the exosomal export of miRNAs in stress responses^22,33^. HuR was shown to replace miR-122 from the target mRNAs and itself bind with the miRNA to facilitate its unloading from the RISC complex and subsequent export^22^. However, we did not find direct binding of HuR to miR-125b, although HuR has been shown earlier to displace miR-125b from its interaction with p53 mRNA^20^. Here we show that HuR is required for miR-125b export, suggesting multiple mechanisms by which HuR can influence the export of miRNAs, including both by displacing miRNAs from target transcripts and by interacting with mediators of exosomal trafficking. The regulation of the cellular level of miR-125b in response to genotoxic stress appears to be independent of Ago2, as the knockdown of Ago2 did not affect the export of miR-125b, nor did it affect the endogenous level of miR-125b. This conforms to earlier studies which have shown that depletion or knockout of Ago2 in cells or animals did not affect the cellular levels of most miRNAs^34–36^. It is thought that most mature extracellular miRNAs are associated with Ago2, which protects miRNAs from degradation, although Ago2-associated miRNAs have also been found in extracellular vesicles and therefore the processes of Ago2-associated and exosome-mediated export of miRNAs may not be completely independent^37,38^. Our study reinforces the idea that extracellular export of miRNAs is not only a means of cellular communication by delivery of the miRNA to recipient cells but is also a mechanism to regulate miRNA levels in the parent cell and thereby control the expression of the miRNA target genes.

The depletion of the cellular miR-125b level and the increase in cytoplasmic HuR level, combined with the stabilization of p53 protein, results in the increase in p53 level allowing it to reach a peak around 4–6 h post UV irradiation. We have now shown that p53 also induces the transcription of miR-125b, thereby enhancing cellular miR-125b level, which would then contribute to the repression of p53 mRNA translation and reduce de novo p53 protein synthesis (Fig. 7g). This, combined with the degradation of already existing p53 protein by Mdm2-mediated degradation, brings down p53 to the basal level and generates the pulse of p53. p53 regulates the transcription of several miRNAs such as the miR-34 family, miR-145, miR-192, miR-215, and miR-107^17^. We show miR-125b as a UV-inducible transcriptional target of p53, thereby constituting a negative feedback loop that regulates miR-125b expression. The downstream targets of the miRNAs transcribed by p53 include genes involved in cell cycle regulation, apoptosis, and tumorigenesis, thereby constituting a regulon of genes indirectly regulated by p53. Interestingly, miR-125b has been found to regulate multiple genes in the p53 transcriptional regulatory network thereby forming a co-regulon that buffers and fine-tunes p53 transcriptional network activity^39^.

Together with transcriptional upregulation, p53 was also found to enhance the processing of miR-125b. p53 enhances the post-transcriptional processing of several miRNAs with growth-suppressive function, such as miR-16-1, miR-143, and miR-145, in response to DNA damage by interacting with the Drosha processing complex through its association with DEAD-box RNA helicase p68 (DDX5)^18^. We also observe that the interaction between p53 and DDX5 is enhanced in UV-irradiated cells indicating its association with the Drosha complex. Therefore, a combination of p53-mediated transcriptional upregulation and miRNA processing enhances miR-125b level as a delayed response to UV irradiation, thereby contributing to the repression of p53 and HuR synthesis and reduction of p53 to basal levels. Together, these intricate feedback loops, involving HuR, miR-125b, and p53 itself, contribute to the fine-tuned temporal regulation of p53 expression in response to genotoxic stress.

## Methods

### Plasmid constructs

The 525 nucleotides putative miR-125b promoter region was amplified from genomic DNA isolated from MCF7 human breast carcinoma cells using specific primers (5′AAGTGGTACCGTTCAGAACGCTATTCGTCTTTACA3′ and 5′AGGTCTCGAGACAAT GTTTTCTTTCTTGAGACAAAGCA3′) and cloned upstream of firefly luciferase gene between Kpn I and Xho I restriction sites in a promoterless vector pGL3basic (gift from D. Biswas, CSIR-IICB Kolkata, India). The predicted p53-binding sites (nt. 100-114, 118-127, and 219-228) were mutated using megaprimer-based site-directed mutagenesis and cloned into the same vector to generate mut1, mut2, and mut3 respectively. pCDNA3-p53 construct containing p53 ORF sequence was a gift from S. Das, IISc Bangalore, India. HuR cDNA cloned with a myc-his tag cloned in pCDNA3.1 vector between Nhe I and Eco RI restriction sites was used for mammalian expression of HuR^40^. miR-125b shRNA cloned under H1 promoter in pSuper vector, together with EGFP gene under a CMV promoter to generate pSuper-EGFP-miR-125b plasmid. Primary miR-125b (88 nucleotides) was cloned into pCDNA3.1 vector between Hind III and Eco RI sites by PCR amplification from a pri-miR-125b oligo using forward and reverse primers to generate pCDNA3.1-miR-125b.

### Cell culture and treatment

MCF7 human breast carcinoma cell line (HTB-22™, ATCC, USA) was maintained in Dulbecco’s Modified Eagle’s Medium (Thermo Fisher Scientific), supplemented with 10% Fetal Bovine Serum (Thermo Fisher) and 1% Pen-strep solution (Thermo Fisher). MDA-MB-231 human breast carcinoma cell line (CRM-HTB-26™, ATCC) was maintained in Dulbecco’s modified Eagle’s medium (Thermo Fisher Scientific), supplemented with 10% fetal bovine serum (Thermo Fisher) and 1% Pen-strep solution (Thermo Fisher). MCF10A immortalized human breast epithelial cell line (CRL-10317™, ATCC) was maintained in DMEM/F12 medium (Thermo Fisher) supplemented with 5% horse serum, 20 ng/ml EGF, 0.5 mg/ml hydrocortisone, 100 ng/ml cholera toxin, and 10 μg/ml insulin. Cells were exposed to a 10 J/m^2^ pulse of UV-C radiation in a UV crosslinker (CL-1000, UVP). Cells were transfected with plasmids using polyethylenimine (PEI) transfection reagent (BioBharati, India). DNA amount for transfection was equalized by pGEM T plasmid. Cells were transfected with siRNAs for p53 (siGENOME SMART pool, M003329, GE Dharmacon), HuR (siGENOME SMART pool, M003773, GE Dharmacon) Ago2 (On-Target plus siRNA EIF2C2, J-004639-05-0002, Horizon Discovery Dharmacon) and non-targeting Control siRNA (siGENOME SMART pool, GE Dharmacon) or (SIC001, Sigma) using either Lipofectamine 2000 (Thermo Fischer Scientific) or Turbofect (Thermo Fischer Scientific) according to manufacturer’s protocol. 20 µM exosomal inhibitor GW4869 (Sigma Aldrich), 1 µg/ml of eukaryotic transcription blocker Actinomycin D (Sigma Aldrich), and 20 µM Pifithrin α (Sigma Aldrich) were added to cells as described.

### Immunoblotting

Cell lysates, prepared either in RIPA buffer to prepare whole cell lysate or S10 lysis buffer to prepare cytoplasmic lysate were resolved by SDS–PAGE. Proteins were immunoblotted using HuR (3A2, sc-5261, Santa Cruz Biotechnology), p53 (DO-1, sc-126 Santa Cruz Biotechnology), Flotillin-1 (D2V7J, Cell Signaling Technologies), GM-130 (D6B1, Cell Signaling), Rab7A (NBP2-24591SS, NovusBio), DDX5 (MAB6370, R & D Systems), Ago2 (C34C6, Cell Signaling), GAPDH (FL-335, Santa Cruz Biotechnology or A00192, GenScript), His (BB-AB0010, BioBharati Life Science), and β-actin (A00730, GenScript) primary antibodies. HRP- conjugated anti-rabbit (7074S, Cell Signaling) and anti-mouse (7076S, Cell Signaling) antibodies were used as secondary antibodies. A chemiluminescent signal was detected using a Femtolucent ECL plus-HRP kit (G Biosciences).

### Reporter assay

Cells were transfected with wild-type and mutated constructs of putative miR-125b promoter sequence along with pGL3 basic vector containing firefly luciferase gene, p53 or control siRNA, and a Renilla luciferase construct for transfection normalization. Cells were lysed with passive lysis buffer post 48 h of transfection. Luciferase assay was performed using the Dual-Glo Luciferase Assay system (Promega) following the manufacturer’s protocol. Luminescence was measured in a Plate Chameleon (Hidex) multimode microplate reader as per the manufacturer’s protocol.

### Quantitative PCR and miRNA estimation

Total cellular or extracellular RNA was extracted using RNAiso plus (Takara) and polyadenylated using Poly-A polymerase (NEB). cDNA was synthesized from polyadenylated RNA using Oligo(dT)-adapter primer (5’CTGTATCATCTACTGACTATCATTTTTTTTTTT TTTTTTT3’). An adapter-specific universal primer (5’CTGTATCATCTACTGACTATCA3’) and mature miR-125b specific primer (miScript primer assay kit, MS00006629, Qiagen or 5’TCCCTGAGACCCTAACTTGTGA3’) or mature miR-21 specific primer (5’TAGCTTAT CAGACTGATGTTGA3’) was used for miRNA estimation with PowerUp SYBR green (Applied Biosystems) or TB green Premix TaqII (Takara) in StepOnePlus or Applied Biosystems 7500 Real-time PCR systems (Thermo Fisher). Custom-designed forward and reverse primers against the miR-125b gene (5’ATCAAGCTTTGCGCTCCTCTCAGTCCCT3’ & 5’AAGG AATTCAGCACGACTCGCAGCT3’) and forward primer against precursor miR-125b (5’TGCGCTCCTCTCTCAGTCCCT3’) were used to detect primary and precursor miR-125b level. U6B snRNA primer (miScript primer assay kit, MS00033740, Qiagen or 5’CGCAAGGATGACACGCAAATTC3’) was used for the normalization of microRNA concentrations. For absolute quantification of miR-125b concentration, a custom-made miR-125b RNA-oligo (5’rUrCrCrCrUrGrArGrArCrCrCrUrArArCrUrUrGrUrGrA3’, Integrated DNA Technologies) was used as the template and dissolved in nuclease-free water to get a concentration of 50 µM. The RNA oligo was poly-adenylated using poly(A) polymerase and cDNA was synthesized using the oligo(dT)-adapter primer with a final RNA concentration of 10 nM. Serial dilutions of the cDNA were prepared to correspond to RNA concentrations from 10^−11^ to 10^−18^ M and used for quantitative PCR to obtain *C*_T_ values corresponding to each concentration. Average *C*_T_ values were plotted against miR-125b concentrations to generate a standard curve and the curve equation. *C*_T_ values obtained from qPCR assays of cellular and exosomal miR-125b were interpolated into the standard curve to obtain absolute miR-125b concentrations.

### Exosome isolation

For isolation of exosomes, cells were maintained in DMEM supplemented with 10% FBS pre-cleared for exosomes, and the cell supernatant was centrifuged at 1500×*g* for 10 min to remove cells and large particles before concentrating using freeze dryer to 500 µl volume. Samples were recentrifuged at 10,000×*g* for 15 min prior to passing through qEV1/35 nm column (Izon Science) and exosomal fractions were collected as per the manufacturer’s protocol.

### Nanoparticle tracking assay

Cell supernatant from MCF7 cells in 90 mm dishes was used to isolate exosome pellet using exosome isolation reagent (Invitrogen Total Exosome Isolation Reagent, Thermo Fisher) following the manufacturer’s protocol and diluted up to 1 ml with exosome-free 1X PBS. Using a syringe pump, 1 ml of each sample was injected into the field under UV in the nanoparticle tracking system (Nanosight, NS300, Malvern). 5 videos at 1 min each per sample were acquired with camera level 16, detection threshold 5 or 6 at temperature 25 °C and maximum jump length, blur, minimum track length set to auto.

### Immunofluorescence

Cells were fixed with 4% PFA at 2 h post UV exposure. After blocking, cells were incubated in mouse anti-HuR antibody and rabbit anti-Rab7A antibody at 1:500 dilution followed by 1:500 diluted FITC anti-mouse (AS001, ABclonal), Alexa Fluor 568-conjugated anti-rabbit (A11011, Life Technologies) secondary antibody, and DAPI at 0.5 ng/ml (Thermo Fisher, D1306) for nuclear staining. Images were taken in a laser scanning confocal microscope (Zeiss LSM 710) at ×60 magnification.

### RNA-immunoprecipitation (RIP)

50% slurry of protein G sepharose beads (786-284, G Biosciences) was incubated with p53 (DO-1, Santa Cruz Biotechnology) and HuR antibody (3A2, Santa Cruz Biotechnology) and control IgG (BB-AB001, BioBharati Life Science) at 4 °C overnight. Cells were lysed with NT2 buffer and 400 μg of lysate was added to the antibody-coated beads and incubated at 4 °C overnight. Beads were washed with NT2 buffer (with 0.05% NP40). 25% of the immunoprecipitated complex was used for immunoblotting and RNA was isolated from rest by TRIzol reagent followed by qPCR with both primary and mature miR-125b-specific primers along with U6B primers as control.

### Chromatin immunoprecipitation (ChIP)

Chemical crosslinking of DNA–protein complexes was done by adding 27 µL of 37% formaldehyde per ml of cell culture media for 10 min at room temperature. The cross-linking was quenched by adding 100 µL 1.375 M glycine per ml of media. After twice washing with ice-cold PBS, cells were resuspended in lysis buffer and incubated on ice for 10 min. Pelleted nuclei were suspended in nuclei lysis buffer and incubated on ice for 10 min. The lysate was sonicated avoiding foaming and centrifuged at 13,000 rpm for 15 min at 4 °C. DNA concentration was measured from the supernatant and 100 µg chromatin was added to 2 µg antibodies in 300 µL dilution buffer and rotated overnight at 4 °C. 50 µL of protein G-agarose beads were added to the chromatin sample and rotated for 2 h at 4 °C. Beads were washed four times with high salt wash buffer followed by two washes with TE buffer. The beads were resuspended in an elution buffer and incubated at 55 °C for 2 h followed by another overnight incubation at 65 °C to reverse cross-links. DNA was extracted by phenol: chloroform method and precipitated using glycogen and used for PCR.

### Statistics and reproducibility

All graphical data represent the mean ± standard deviation of at least three biological replicates, each with two technical replicates. *, *, $, # signifies a *P* value ≤ 0.05, **, **, $$, ## signifies a *P* value ≤ 0 .01 and ***, ***, $$$, ### signifies a *P* value ≤ 0.005 (Paired two-tailed Student’s *t*-test) between controls and samples indicated in the figures.

### Reporting summary

Further information on research design is available in the Nature Portfolio Reporting Summary linked to this article.

## Supplementary information

Supplementary Information

Description of Additional Supplementary Files

Supplementary Data 1

Supplementary Data 2

Reporting Summary

## Data Availability

All data supporting the findings of this study are available within the article and its supplementary information files. Source data underlying graphs are available in a single Excel file as ‘Supplementary Data 2’. Uncropped and unedited blot, gel, and confocal microscopy images are available as Supplementary Figs. 13–33 in “Supplementary Information”.
