# Supplementary material for: p53 and HuR combinatorially control the biphasic dynamics of microRNA-125b in response to genotoxic stress
Source: Commun Biol. 2023 Jan 27;6:110. doi: 10.1038/s42003-023-04507-9 (PMC9883498; doi:10.1038/s42003-023-04507-9)
Supplement: Supplementary file 1 — Supplementary Information [file 42003_2023_4507_MOESM1_ESM.pdf]

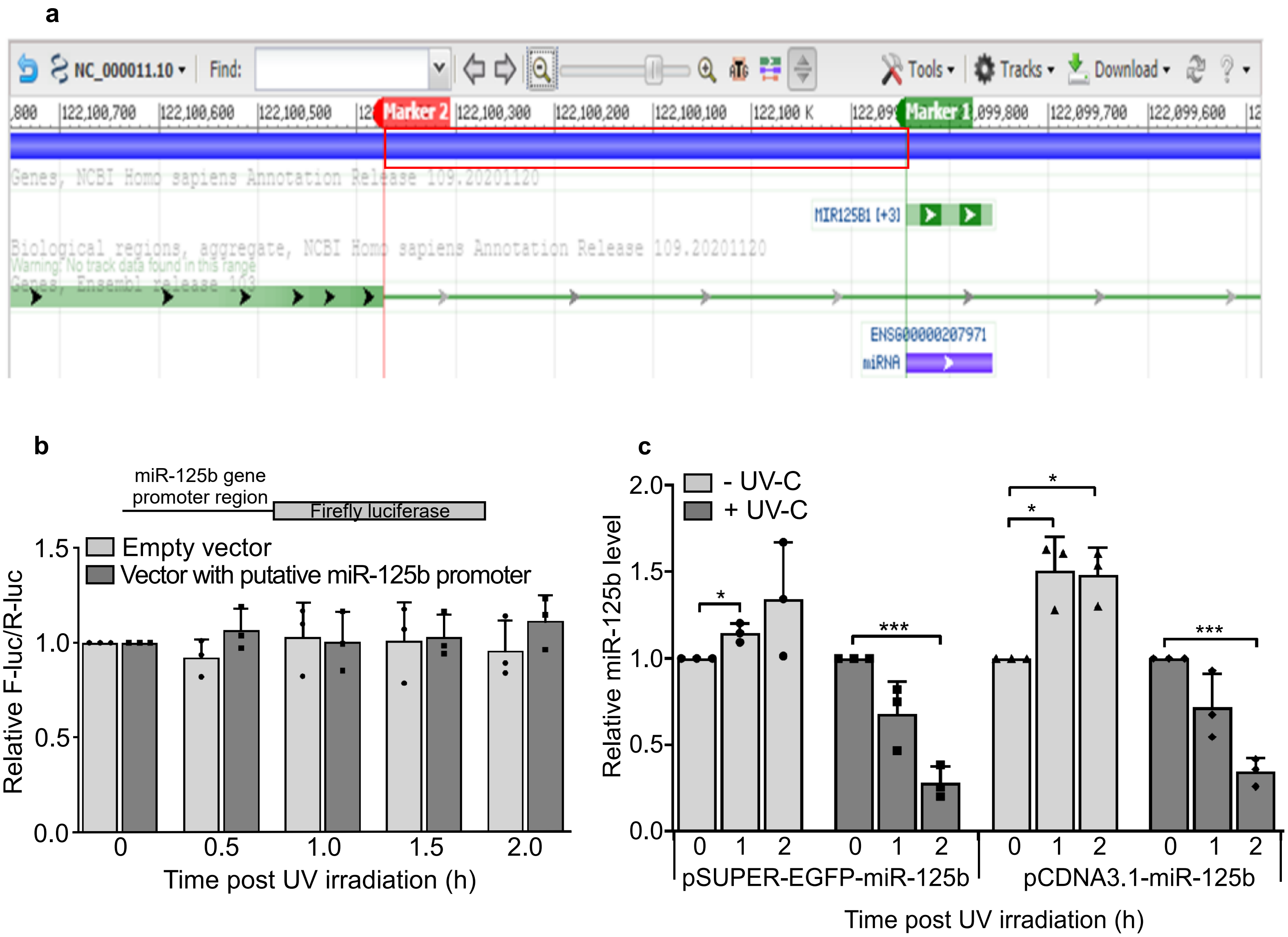

Supplementary Fig 1: (a) Schematic representation of the 525 bp upstream region of the miR-125b gene located in 11q24.1, inserted into the promoterless vector pGL3. (b) Luciferase assay of cells transfected with the firefly luciferase reporter construct containing the putative miR-125b promoter sequence or promoterless (empty) vector consequent to irradiation with UV-C. (c) Estimation of miR-125b level in cells transfected with either a construct expressing miR-125b under a pol III promoter (pSUPER-EGFP-miR-125b) or a pol II promoter (pCDNA3.1-miR-125b) exposed to UV-C radiation. Data represents Mean ± s.d. from three independent experiments. * signifies p value < 0.05, ** signifies p value < 0.01, *** signifies p value < 0.005 (paired two-tailed t-test).

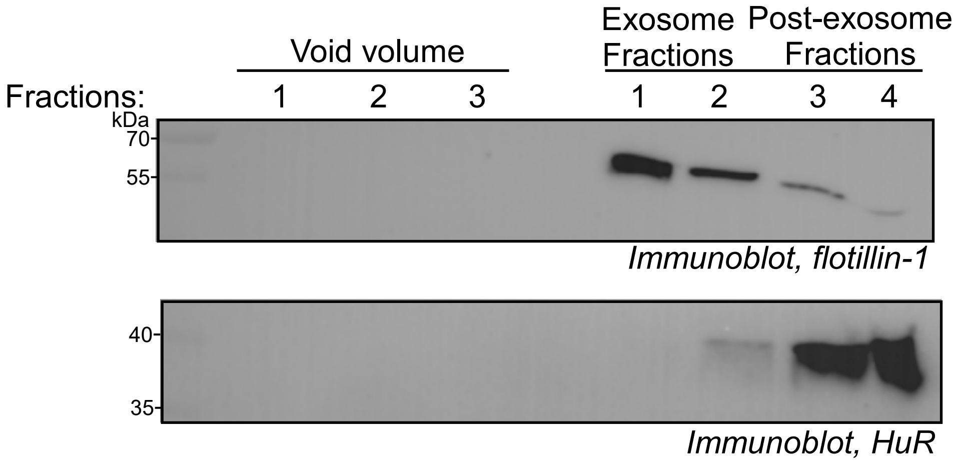

Supplementary Fig 2: Immunoblots of exosomal fractions collected from conditioned media of MCF7 cells not exposed to UVC and probed with flotillin-1 and HuR antibodies.

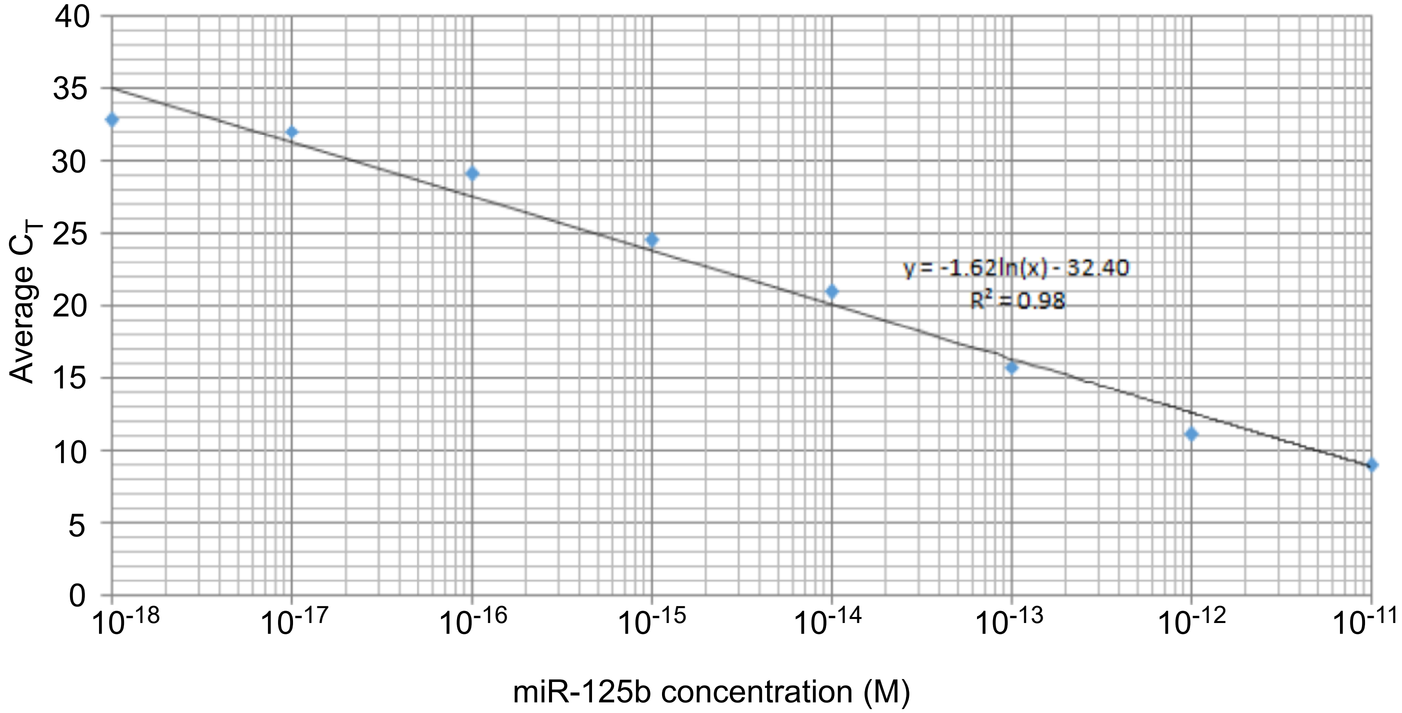

Supplementary Fig 3: Standard curve of miR-125b concentration generated by plotting the average C_T values from 3 independent sets against 8 increasing concentrations of an oligonucleotide corresponding to miR-125b determined by quantitative RT-PCR. Line of best fit corresponding to the plotted values was generated and the curve equation and R^2 values determined.

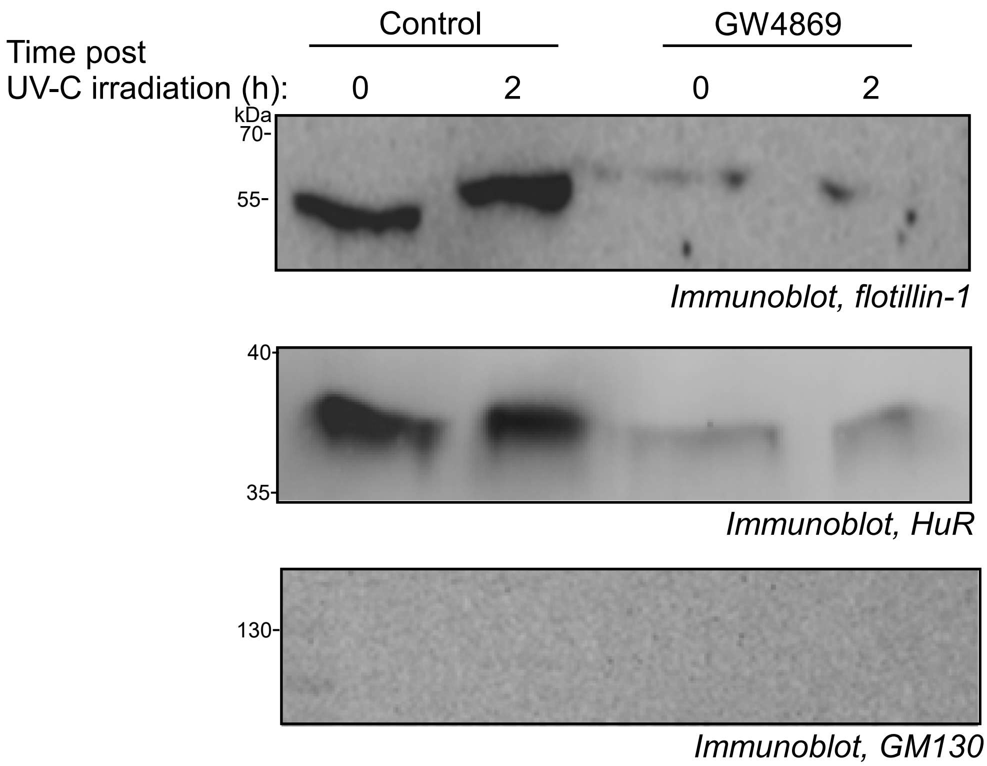

Supplementary Fig 4: Immunoblots of exosomal fractions collected from conditioned media of MCF7 cells, either untreated or treated with the exosome inhibitor GW4869 prior to exposure to UV-C radiation, and probed with flotillin-1, HuR and GM130 antibodies.

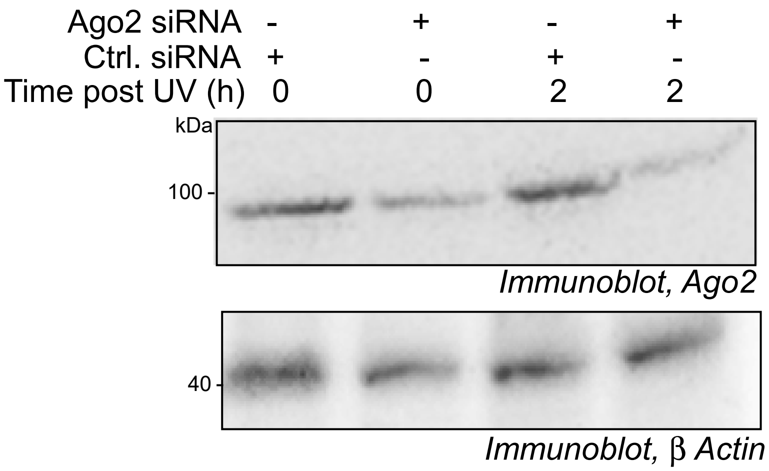

Supplementary Fig 5: Immunoblots of lysates of MCF7 cells transfected with 100 nM control siRNA or Ago2 siRNA for 72 hours and exposed or unexposed to UVC irradiation and collected 2 hours post-UVC irradiation. Lysates were immunoblotted with Ago2 and β Actin antibodies.

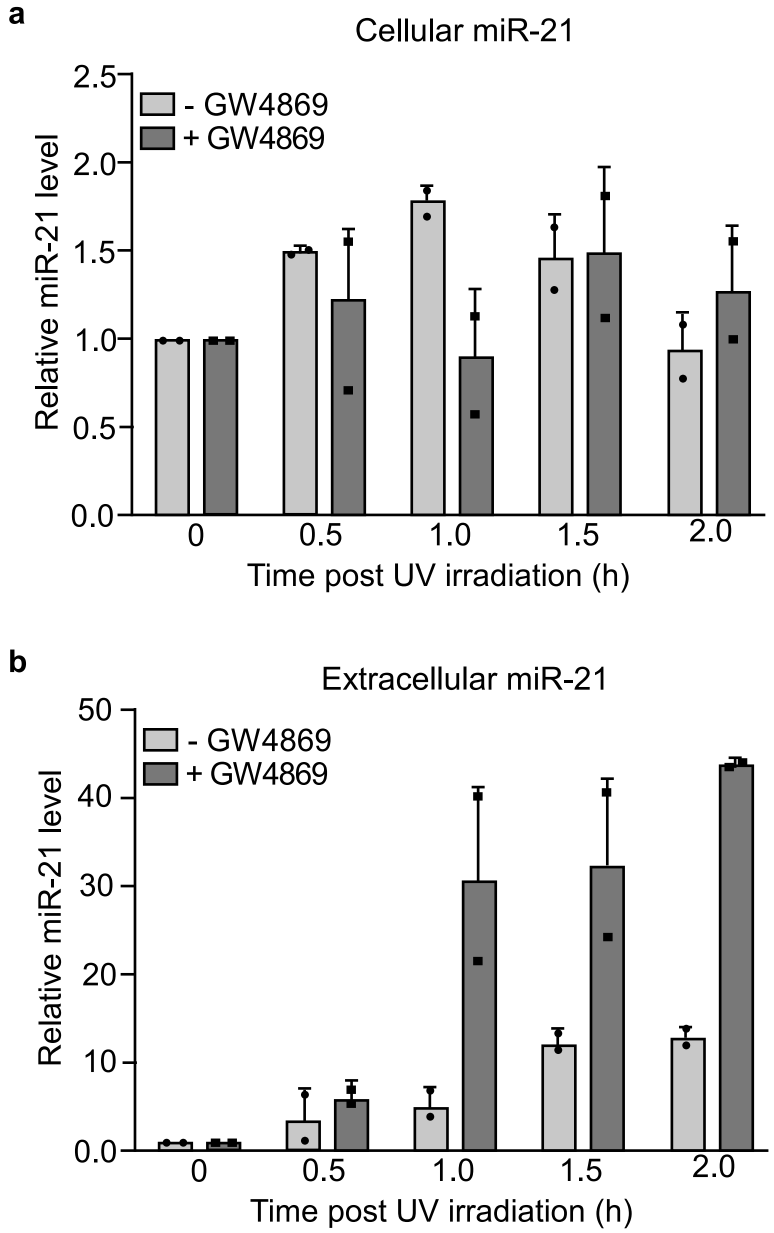

Supplementary Fig 6: Estimation of (a) cellular and (b) extracellular miR-21 from MCF7 cells untreated or treated with 20 μM GW4869 and exposed to UVC irradiation and collected at indicated time points post irradiation, by qRT-PCR. Cellular miR-21 levels were normalized to U6B RNA levels and represented as fold change from respective controls at 0 h time point. Mean ± s.d. from two independent experiments are represented.

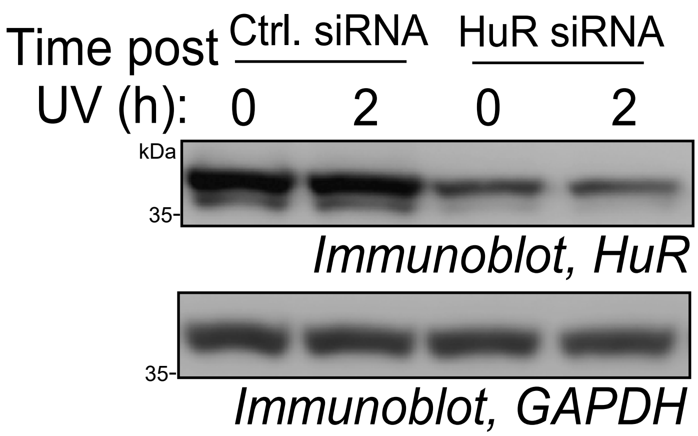

Supplementary Fig 7: Immunoblots of lysates of MCF7 cells transfected with 50 nM control siRNA or HuR siRNA for 48 hours and exposed or unexposed to UVC irradiation and collected 2 hours post-UVC irradiation. Lysates were immunoblotted with HuR and GAPDH antibodies.

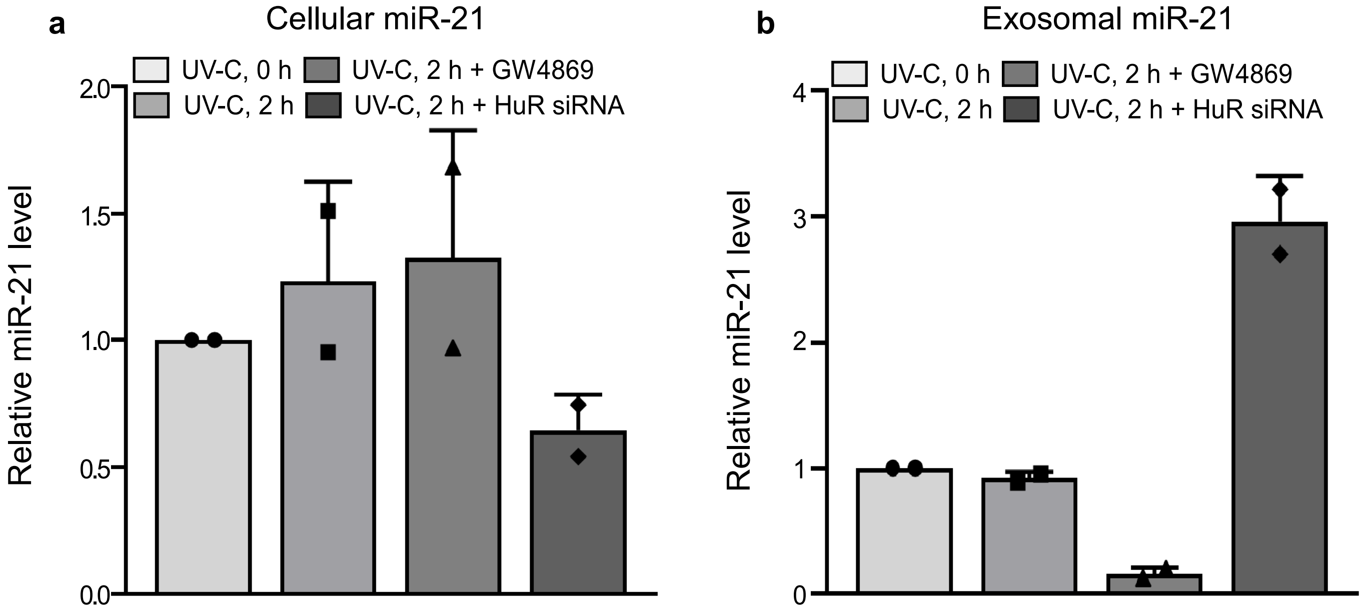

Supplementary Fig 8: Estimation of (a) cellular and (b) exosomal miR-21 from MCF7 cells untreated or treated with GW4869 or transfected with HuR siRNA, and exposed to UVC irradiation and collected at indicated time points post irradiation, by qRT-PCR. Cellular miR-21 levels were normalized to U6B RNA levels and represented as fold change from untreated and untransfected control at 0 h time point. Mean ± s.d. from two independent experiments are represented.

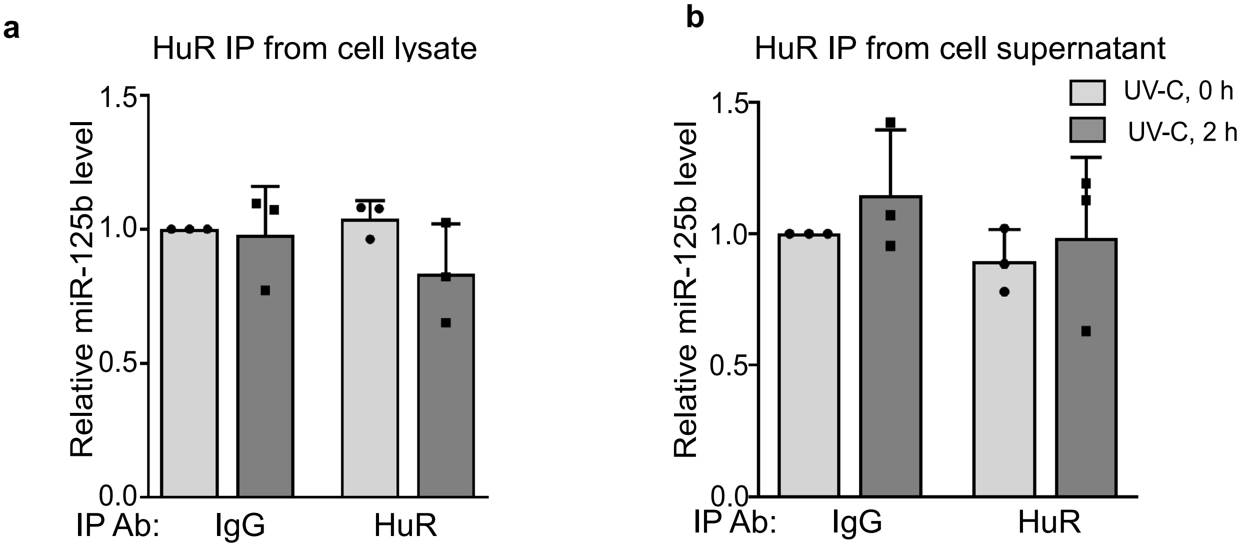

Supplementary Fig 9: MCF7 cells were UV-C irradiated and immunoprecipitation was done with (a) cell lysate and (b) supernatant collected at 0 h and 2 post UVC exposure using control IgG and HuR antibodies. RNA associated with the immunoprecipitates were isolated and miR-125b level was estimated. miR-125b levels are normalised to U6B RNA levels and represented as fold change from control IgG immunoprecipitate for 0 h. Mean ± s.d. from three independent experiments are represented.

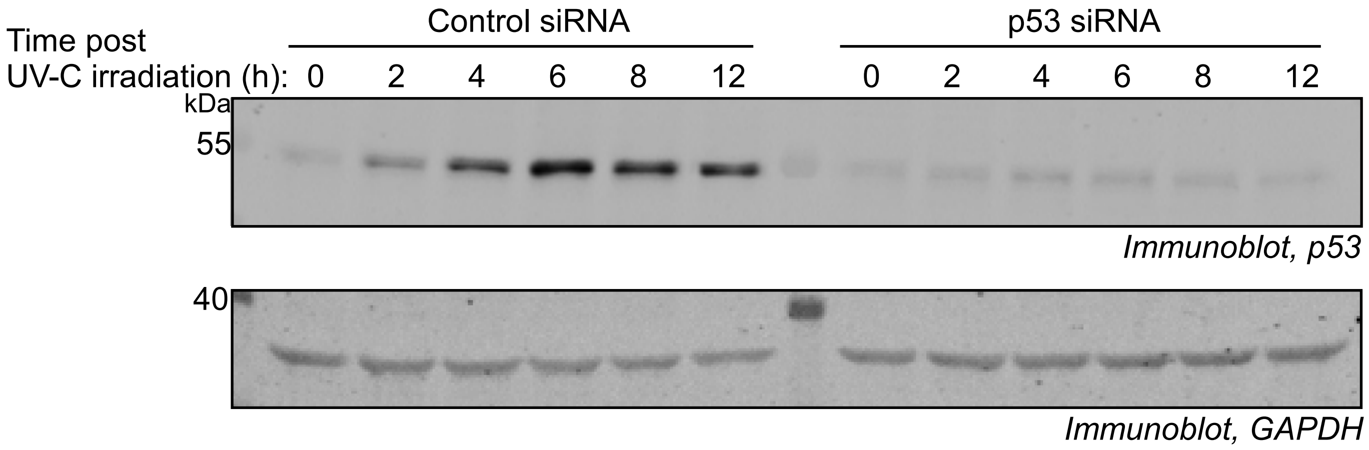

Supplementary Fig 10: Immunoblots of whole cell lysates of MCF7 cells transfected with 50 nM control and p53 siRNA 24 h prior to UV-C irradiation and collected at various time points post UV exposure and probed with p53 and GAPDH antibodies.

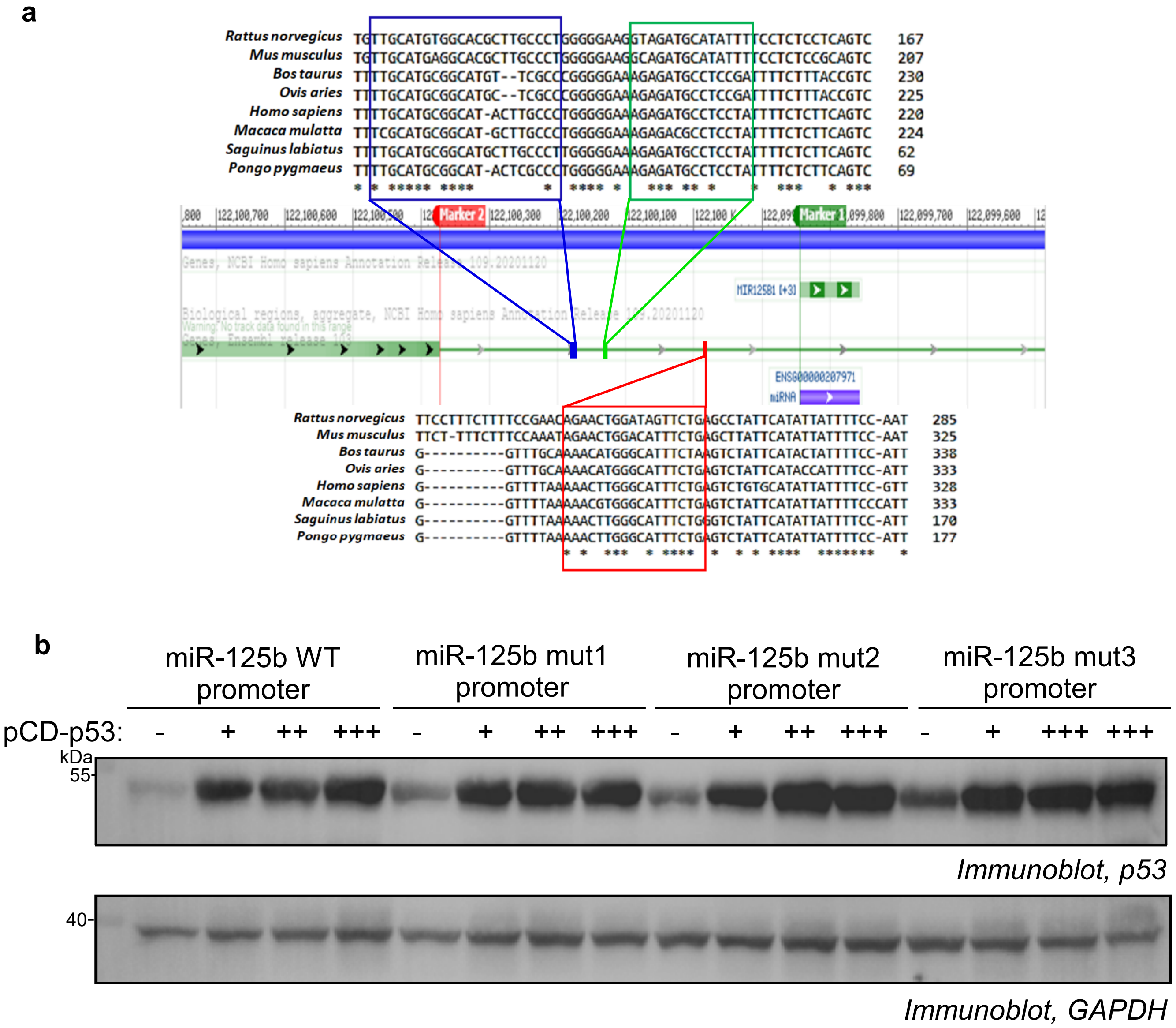

Supplementary Fig 11: (a) Schematic showing the 525 bp region upstream of the miR-125b sequence within the MIR100HG gene with the three predicted p53 binding sites marked in blue, green and red. Evolutionary conservation of the p53 binding sites in mammalian species is indicated by multiple sequence alignment. (b) Immunoblots of lysates of cells transfected with wild type pGL3-miR-125b promoter and three different constructs with predicted p53-binding sites mutated, co-transfected with three increasing concentrations of p53 overexpression construct, probed with p53 and GAPDH antibodies

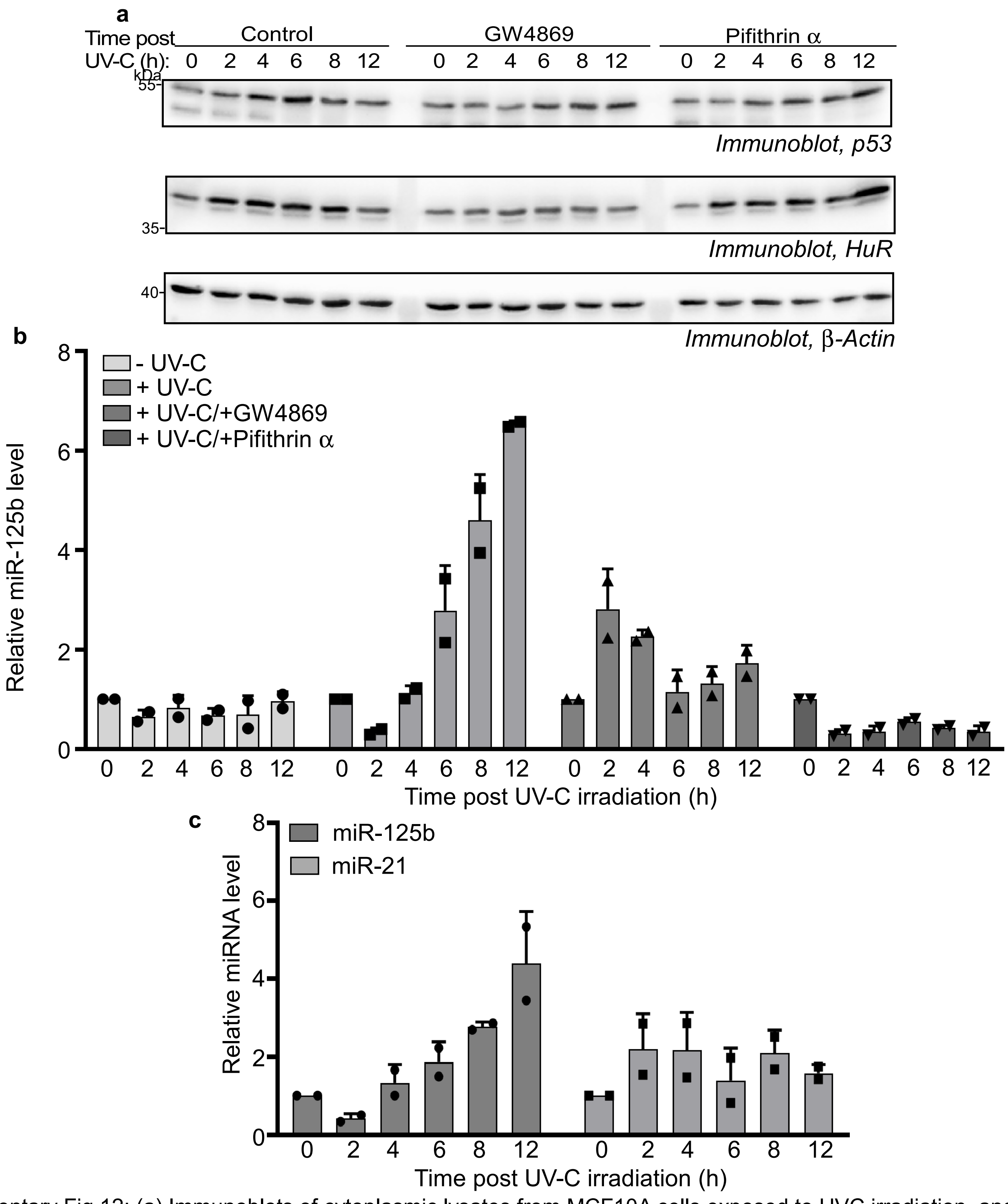

Supplementary Fig 12: (a) Immunoblots of cytoplasmic lysates from MCF10A cells exposed to UVC irradiation, and either untreated or treated with 20 μM GW4869 or 20 μM pifithrin α and collected at indicated time points post-UVC exposure, with p53, HuR and β-Actin antibodies. (b) qRT-PCR of total RNA isolated from UVC-irradiated or non-irradiated MCF10A cells, either untreated or treated with GW4869 or pifithrin α, collected at indicated time points post-UVC exposure using miR-125b specific primers. miR-125b RNA levels were normalized to U6B snRNA levels. Data represents fold change of normalized miR-125b RNA levels at different time points taking 0 hour miR-125b RNA level for each treatment as 1. Mean ± s.d. from two independent experiments are represented. (c) Estimation of miR-125b and miR-21 levels in UVC-irradiated MCF10A cells, collected at indicated time points post UVC exposure. Data represents fold change of normalized miRNA levels at different time points taking 0 hour miRNA RNA levels as 1. Mean ± s.d. from two independent experiments are represented.

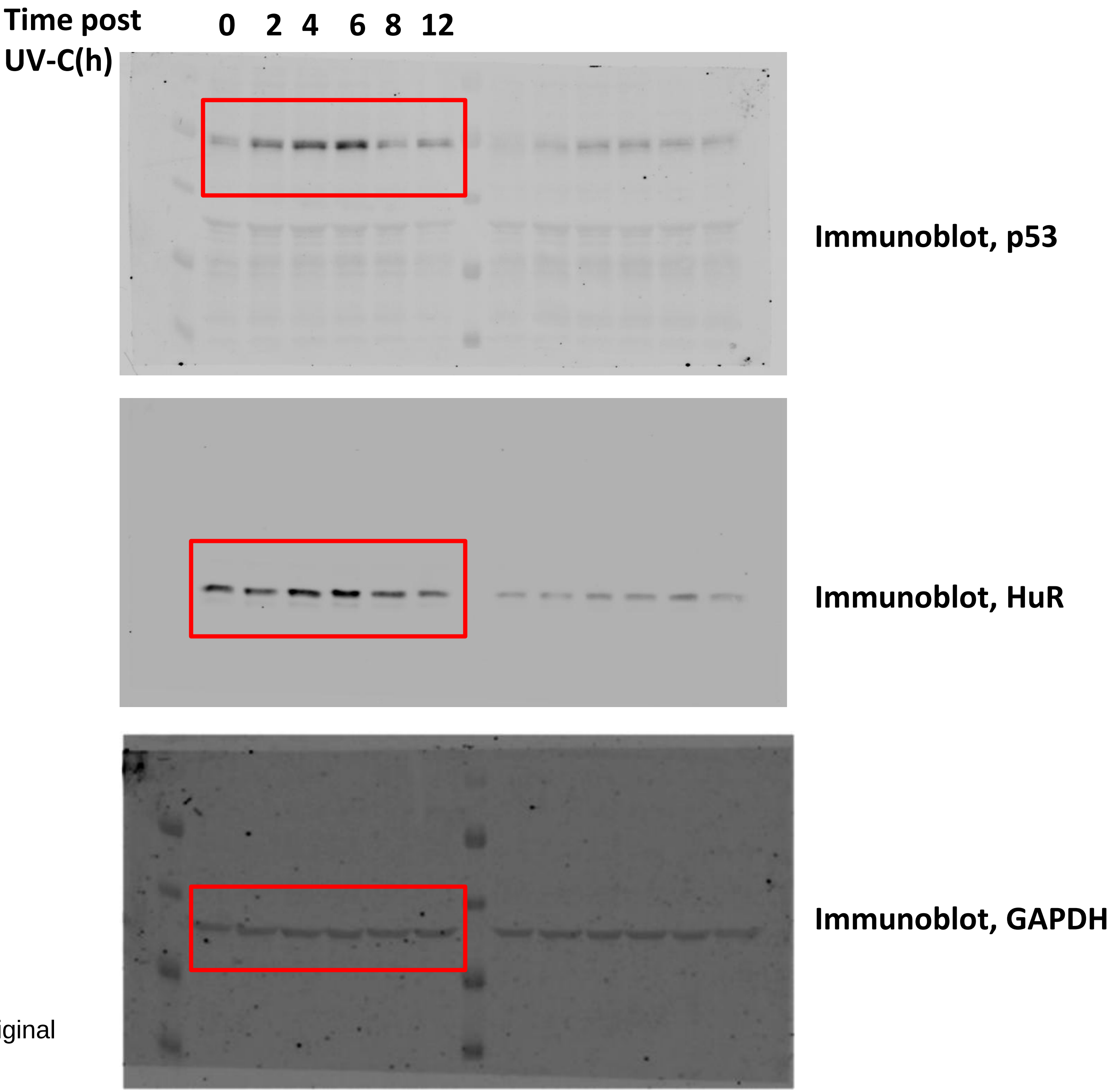

Supplementary Figure 13: Original blots for Fig. 1A

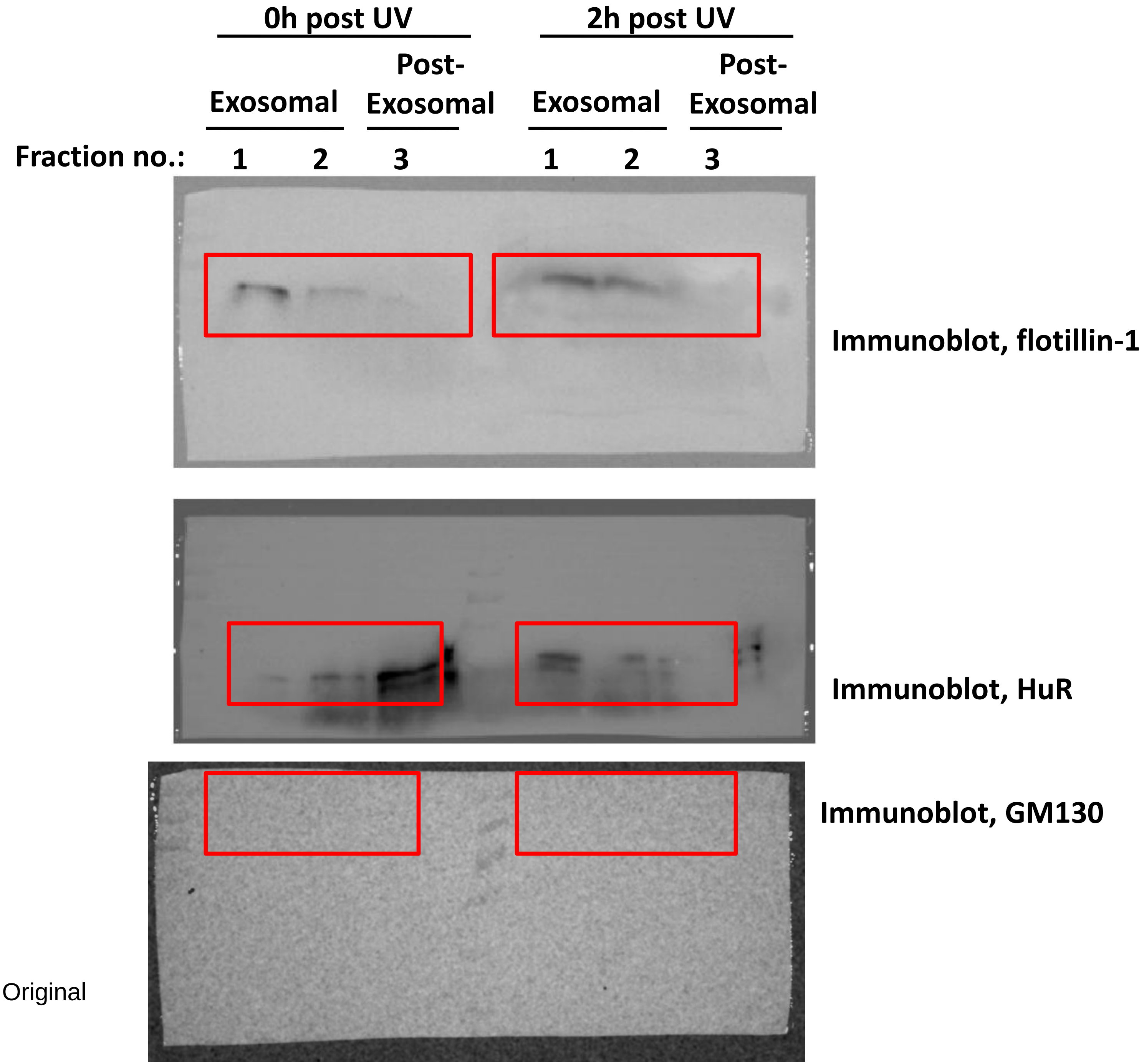

Supplementary Figure 14: Original blots for Fig. 2D

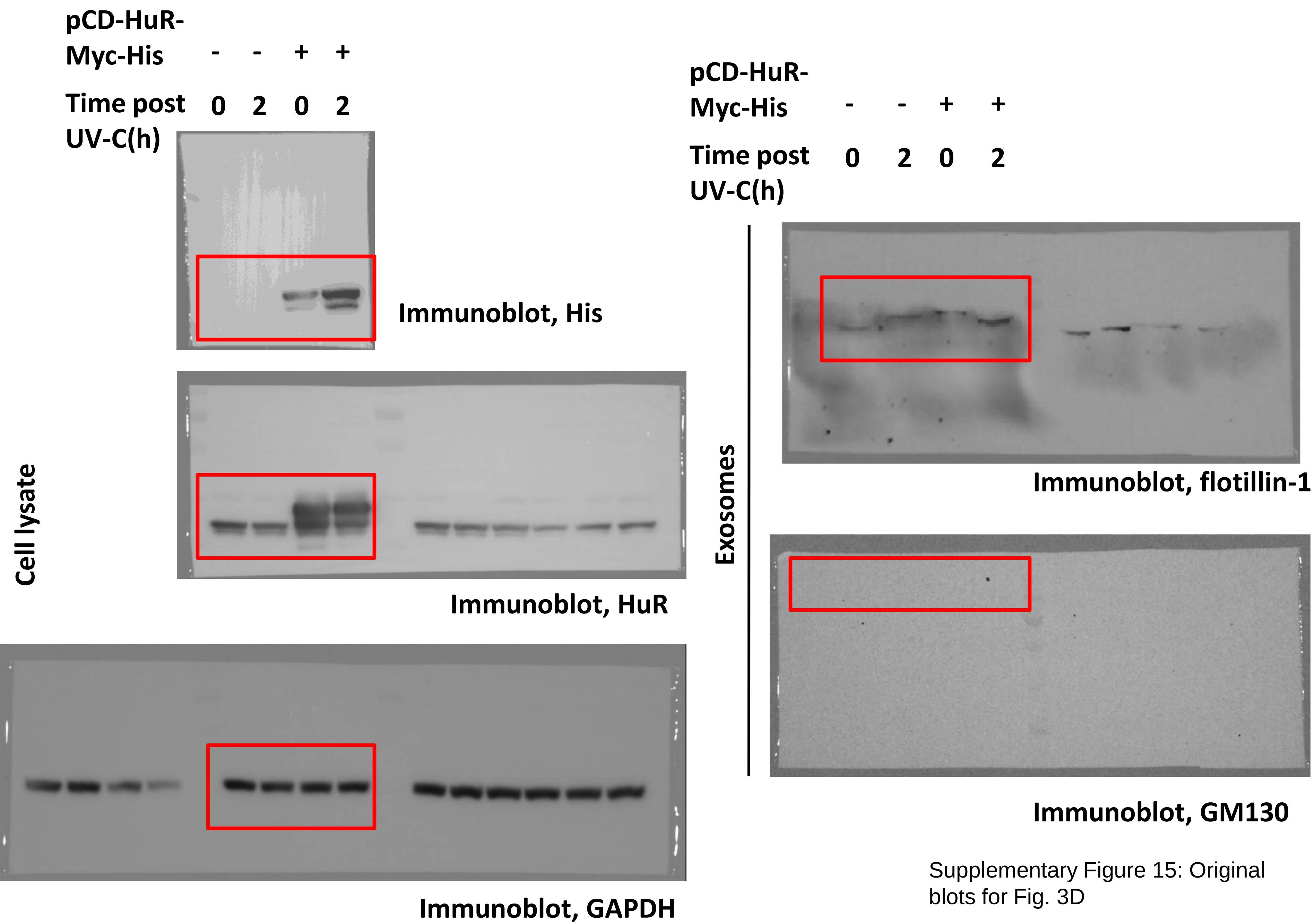

Supplementary Figure 15: Original blots for Fig. 3D

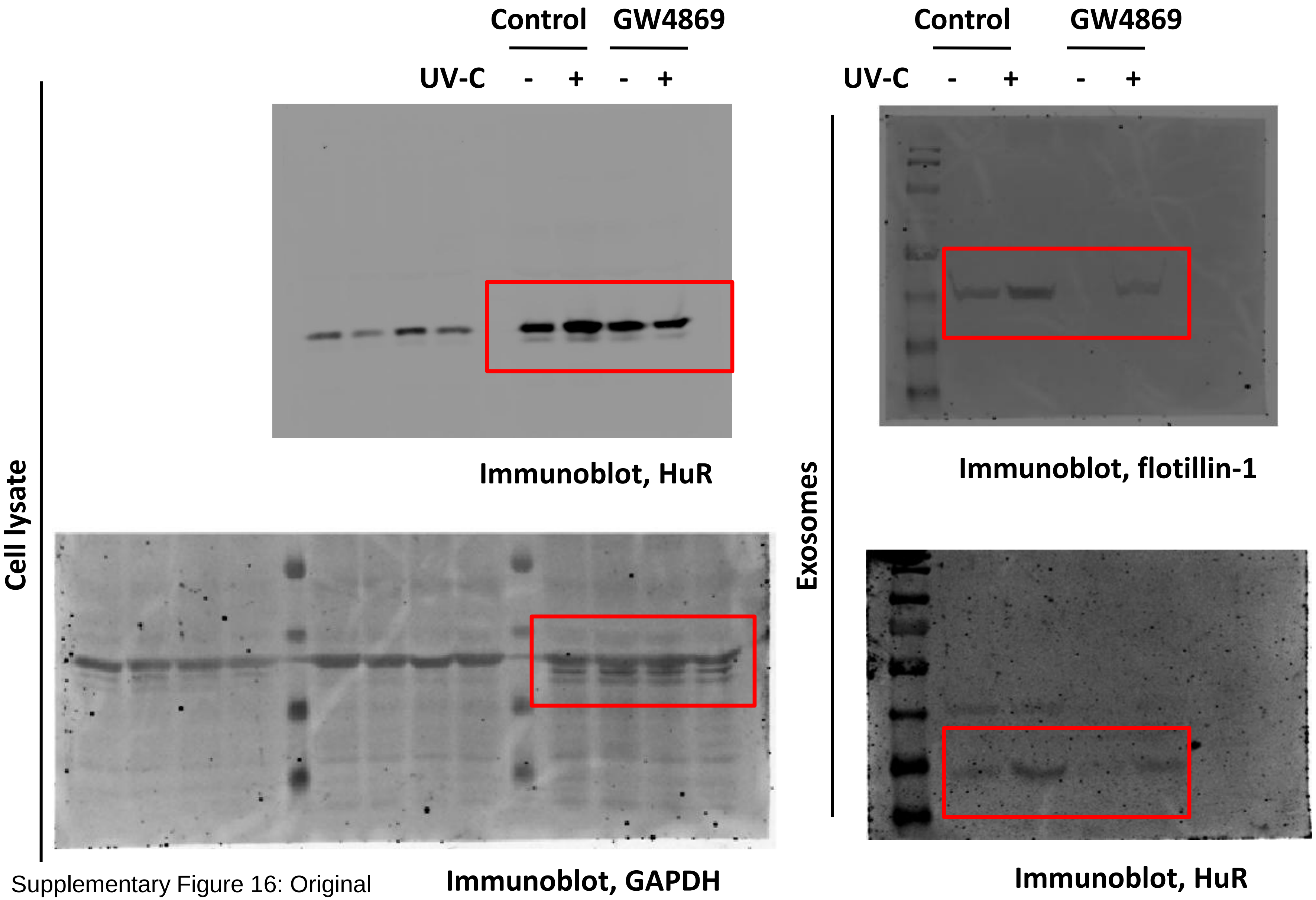

Supplementary Figure 16: Original blots for Fig. 3E

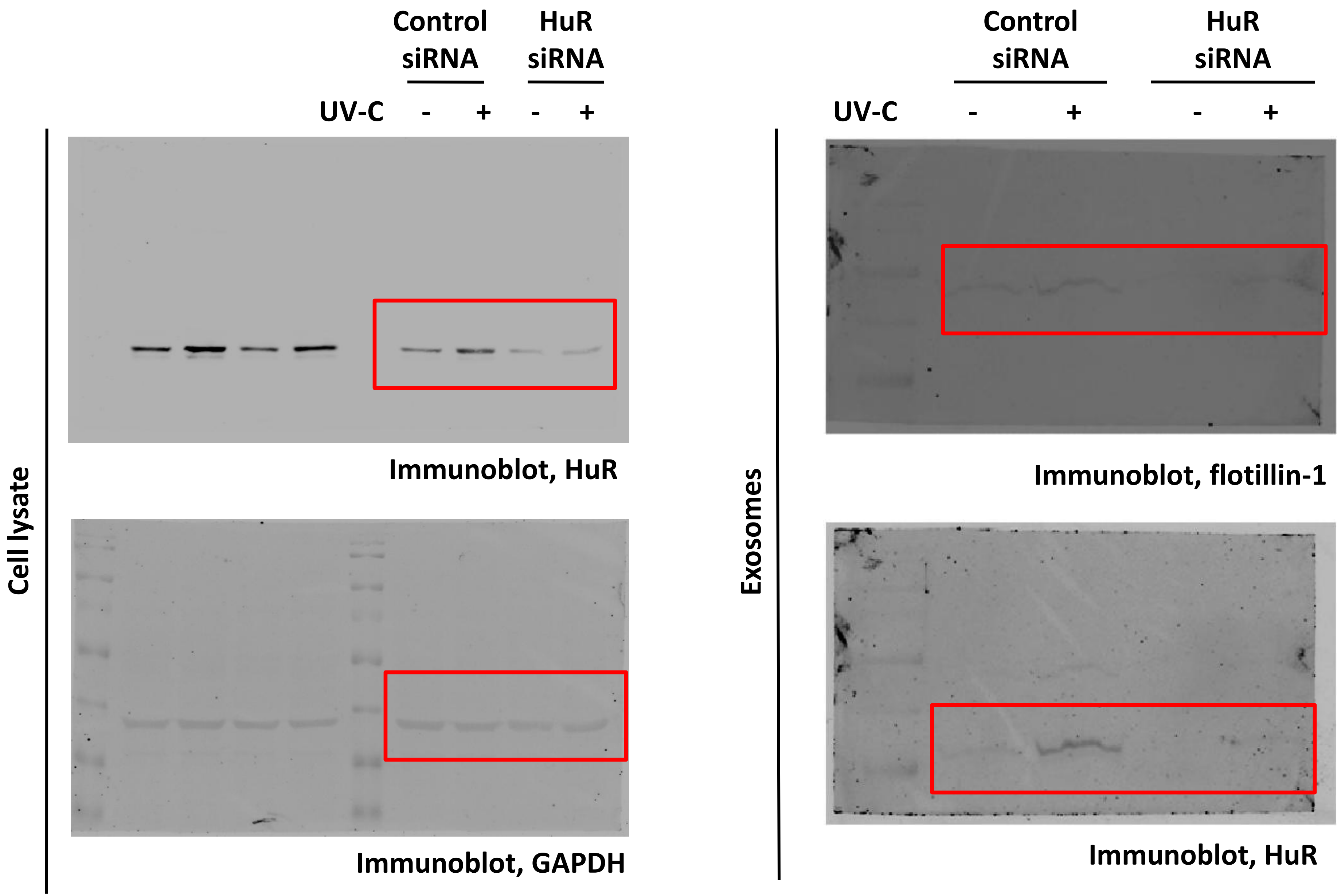

Supplementary Figure 17: Original blots for Fig. 3F

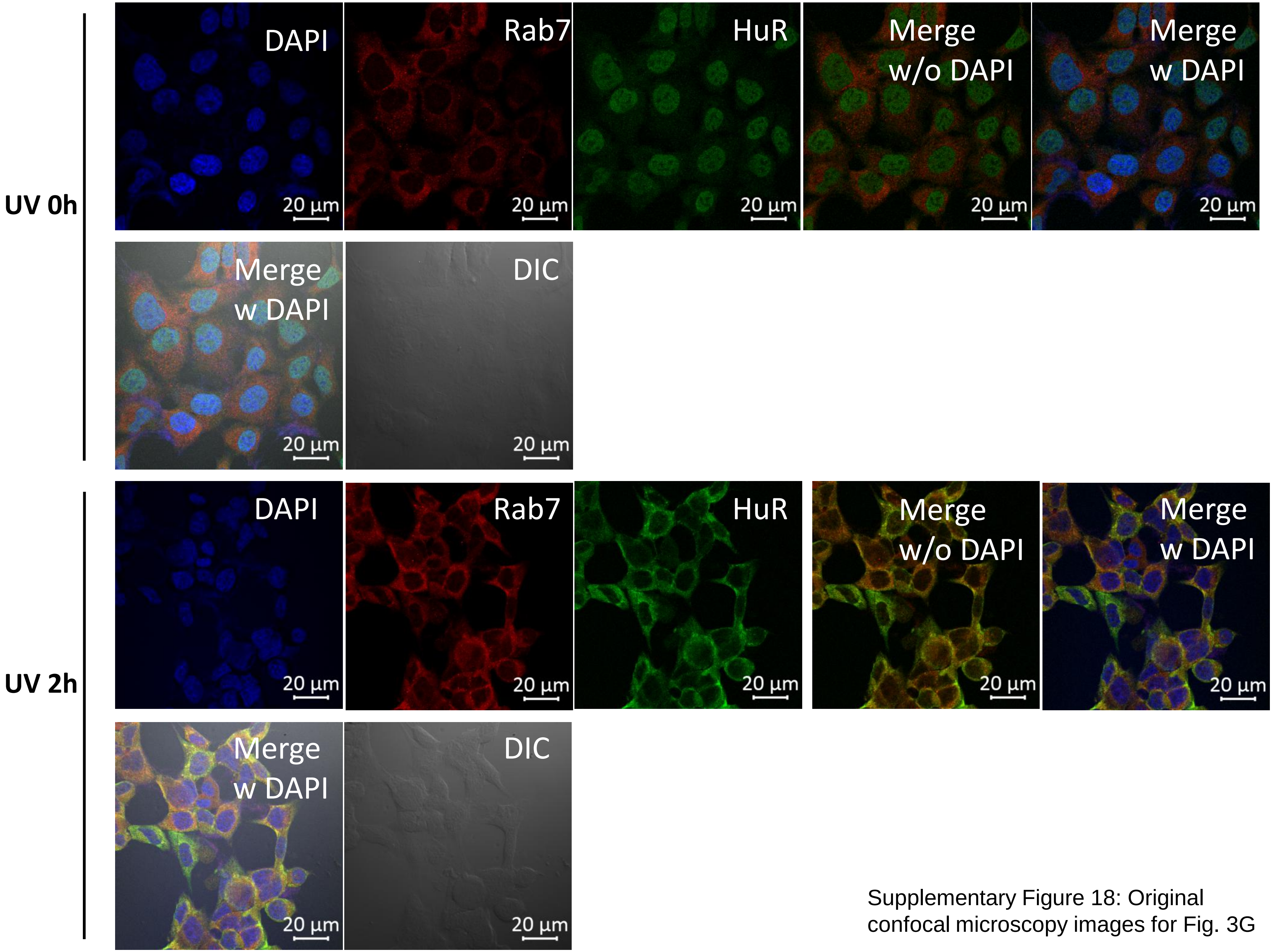

Supplementary Figure 18: Original confocal microscopy images for Fig. 3G

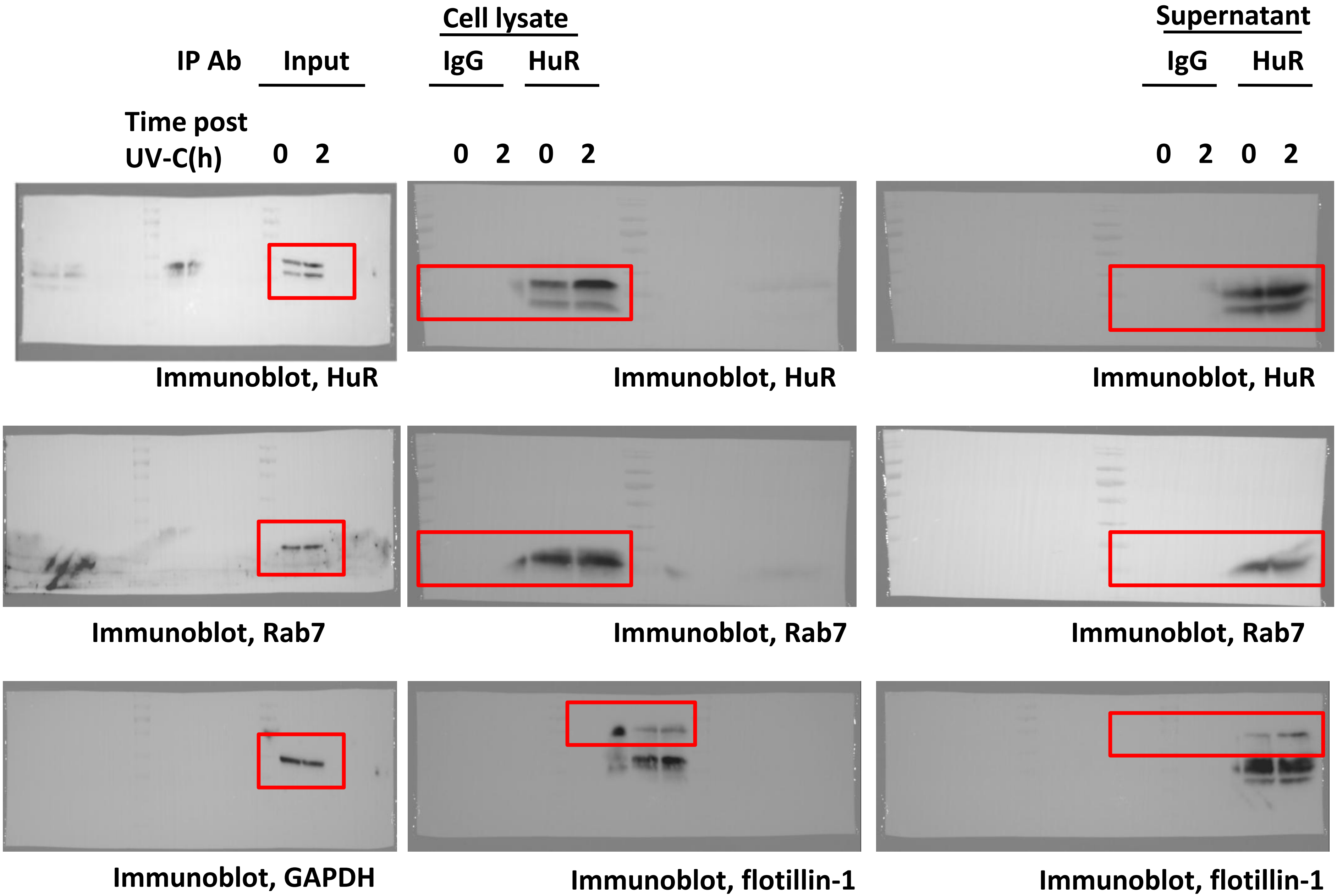

Supplementary Figure 19: Original blots for Fig. 3H

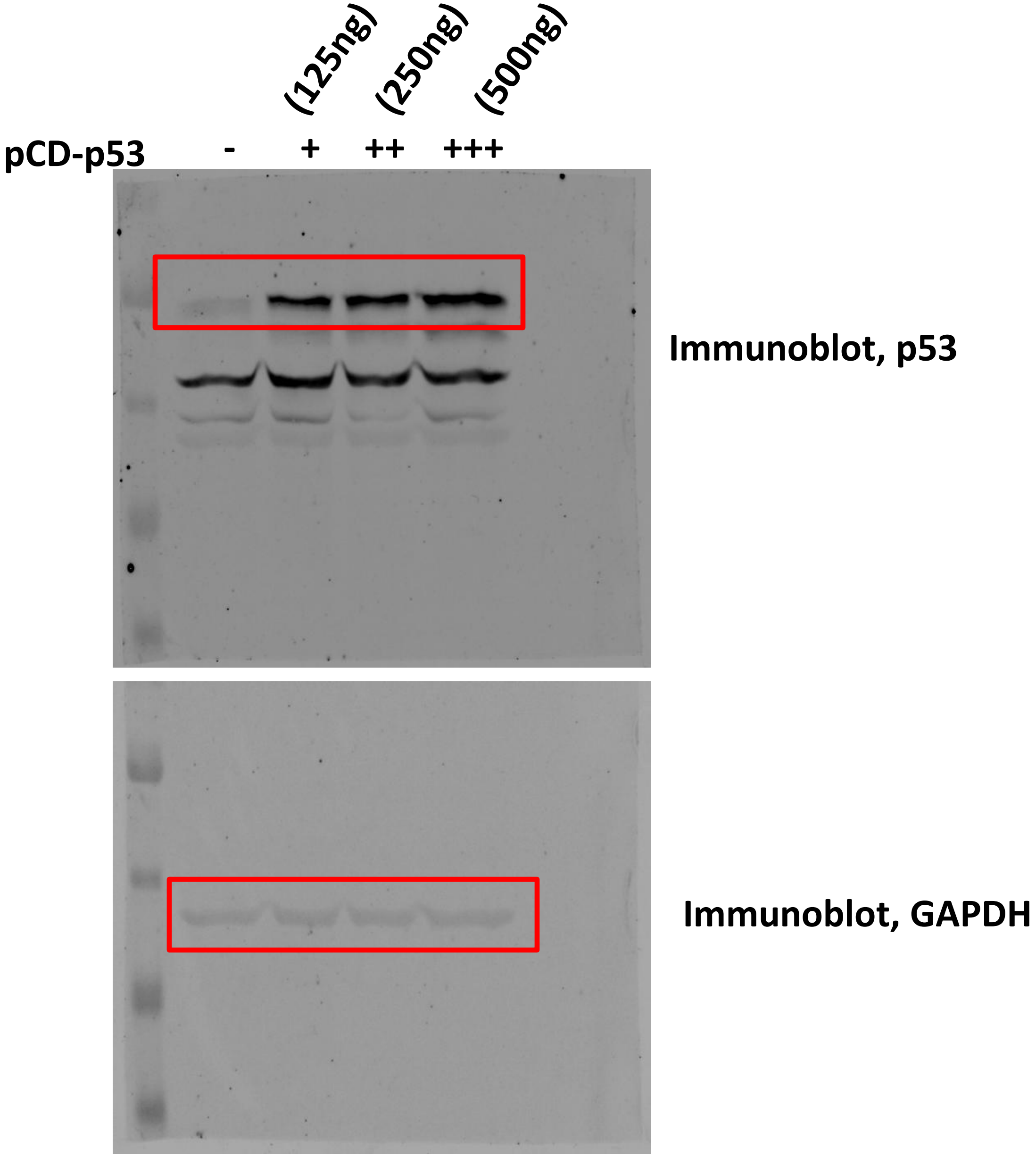

Supplementary Figure 20: Original blots for Fig. 4E

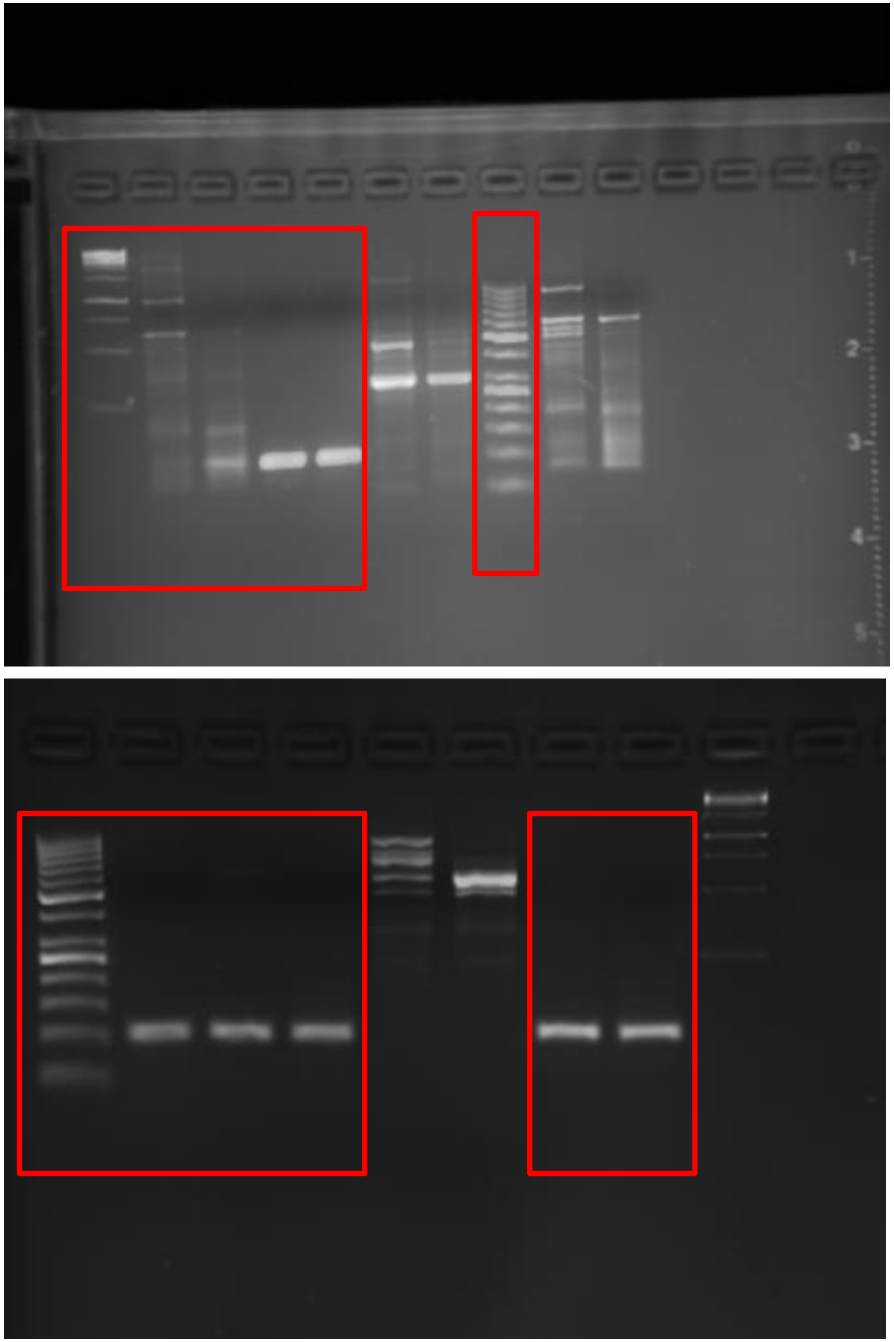

Supplementary Figure 21: Original gel images for Fig. 5B

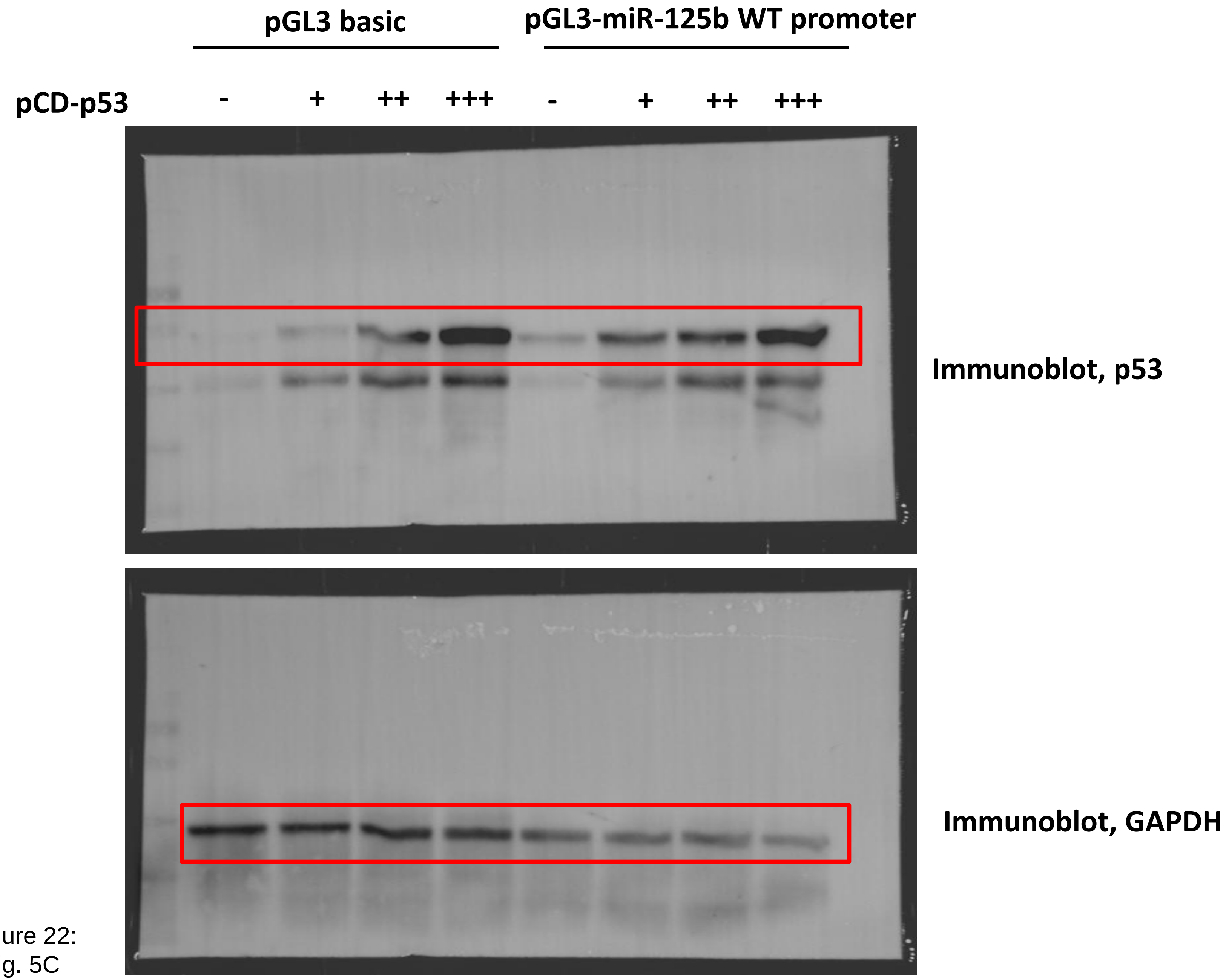

Supplementary Figure 22:
Original blots for Fig. 5C

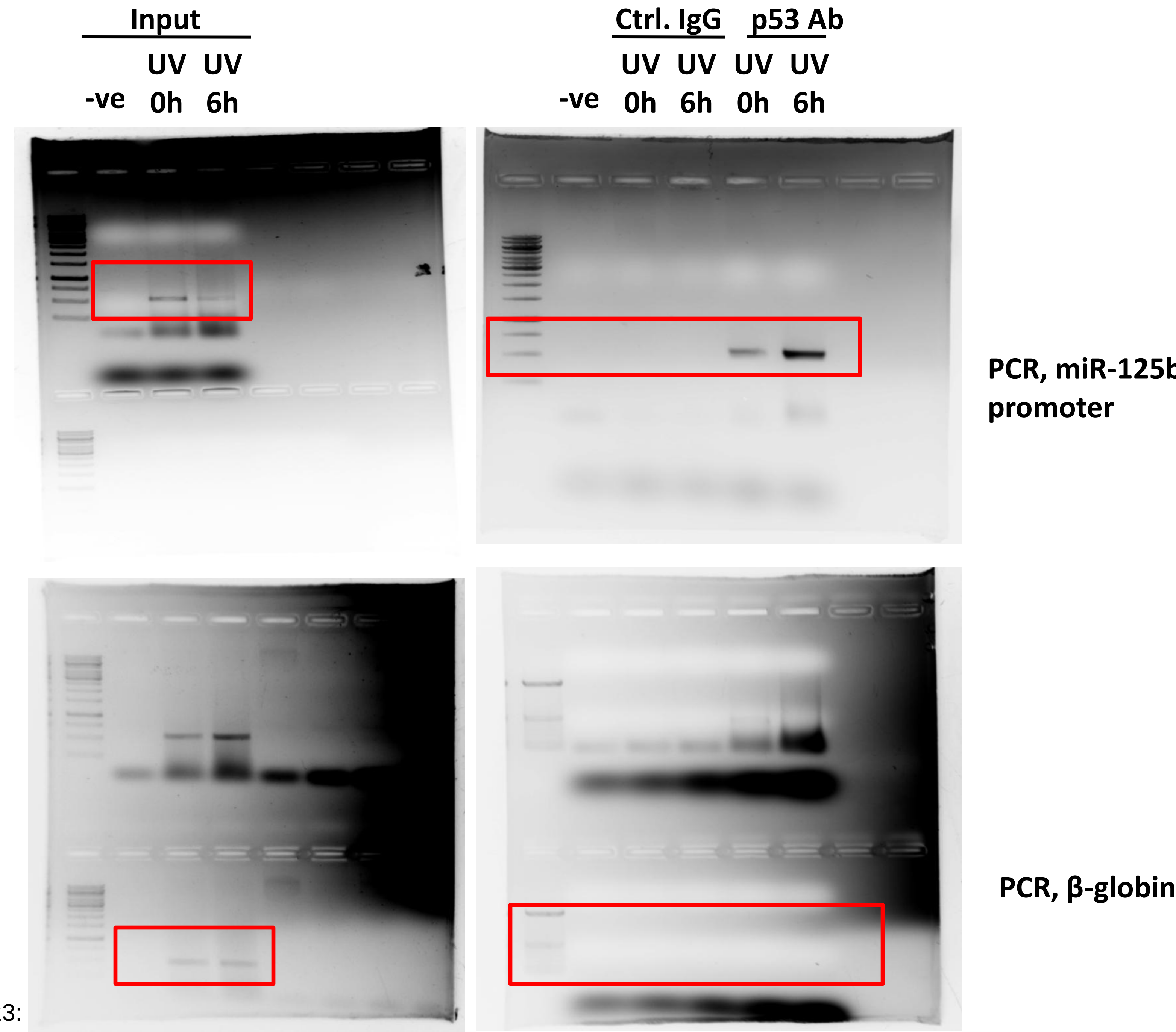

Supplementary Figure 23:
Original blots for Fig. 5G

Figure 6G

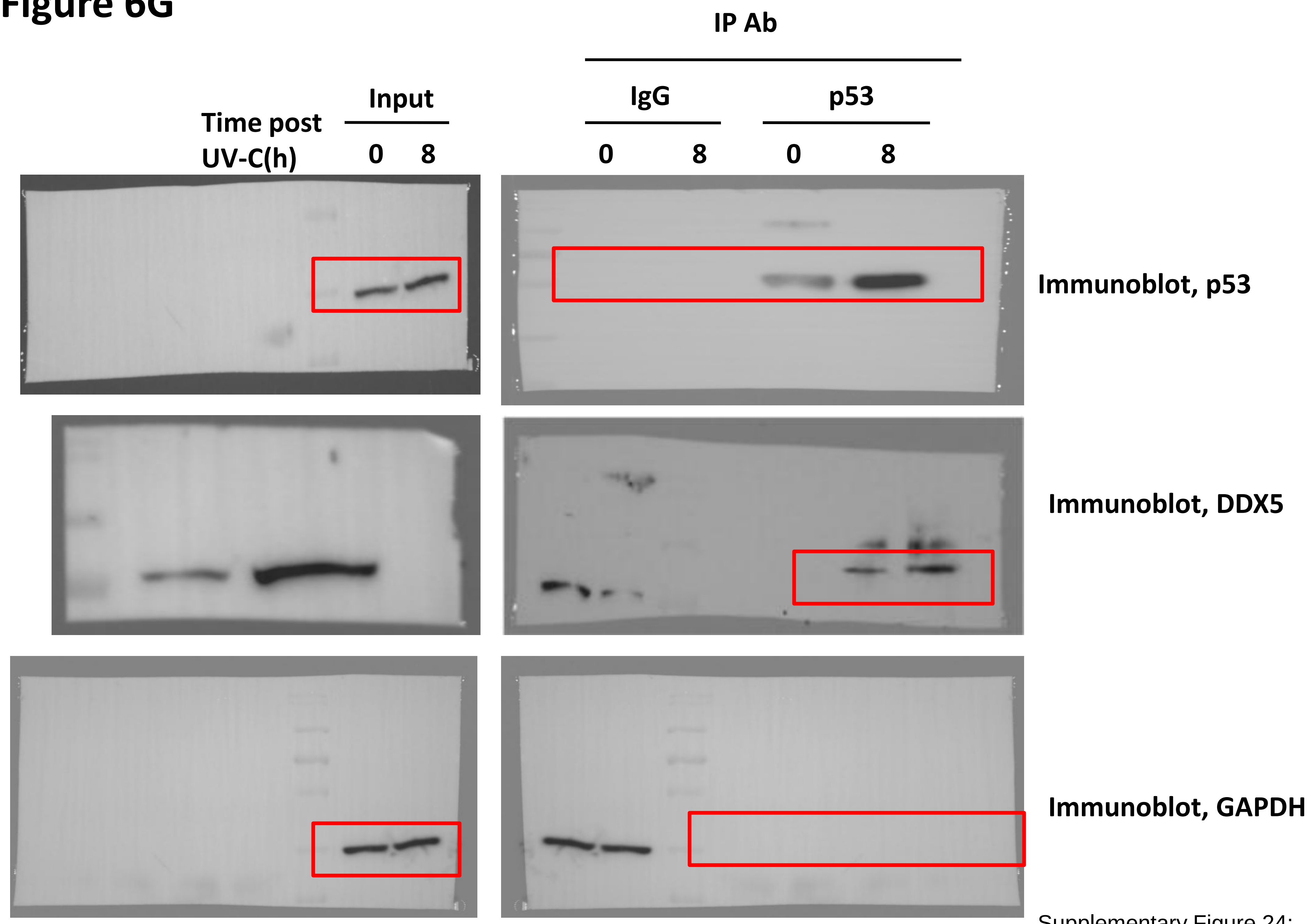

Supplementary Figure 24: Original blots for Fig. 6G

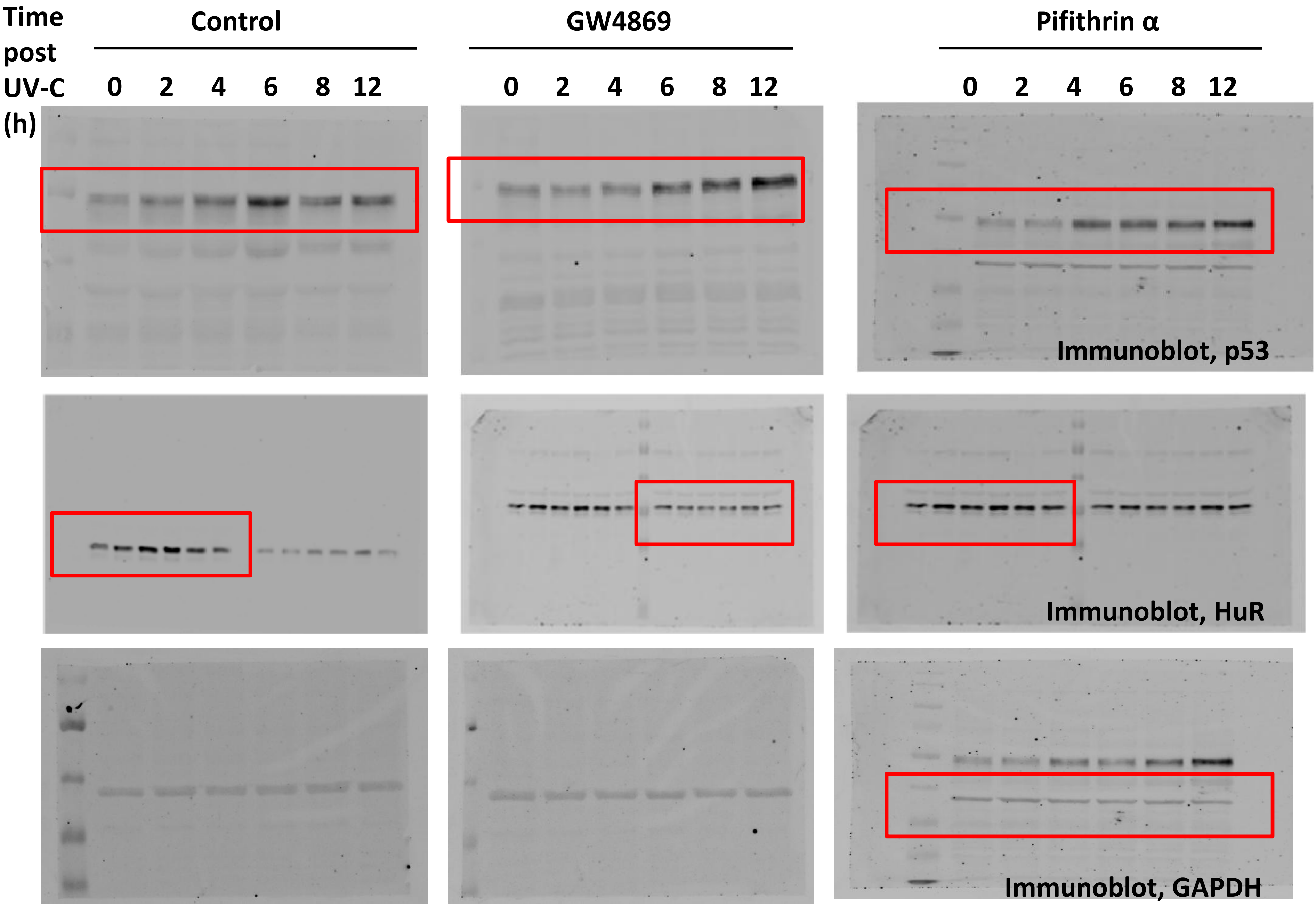

Supplementary Figure 25:
Original blots for Fig. 7B

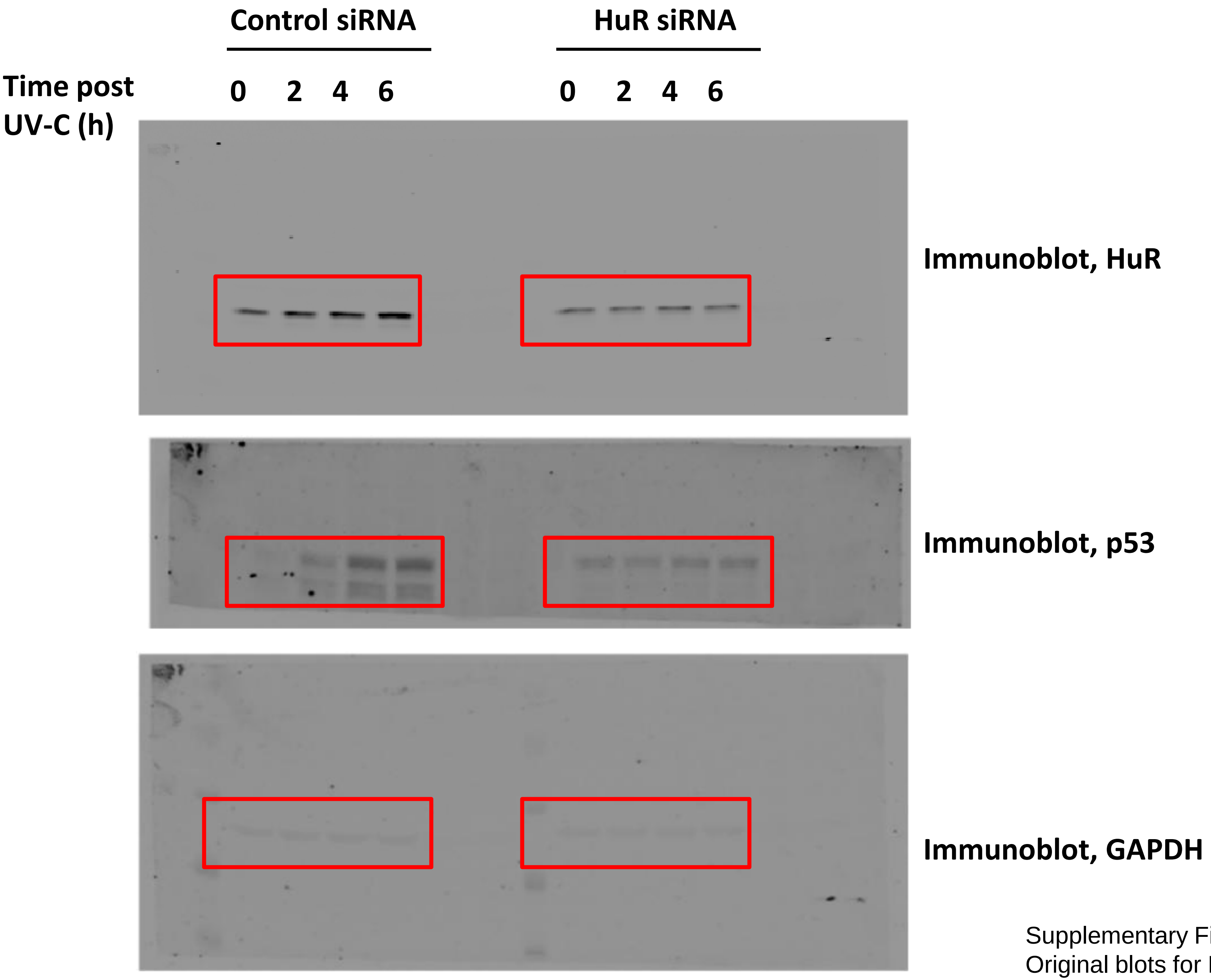

Supplementary Figure 26: Original blots for Fig. 7E

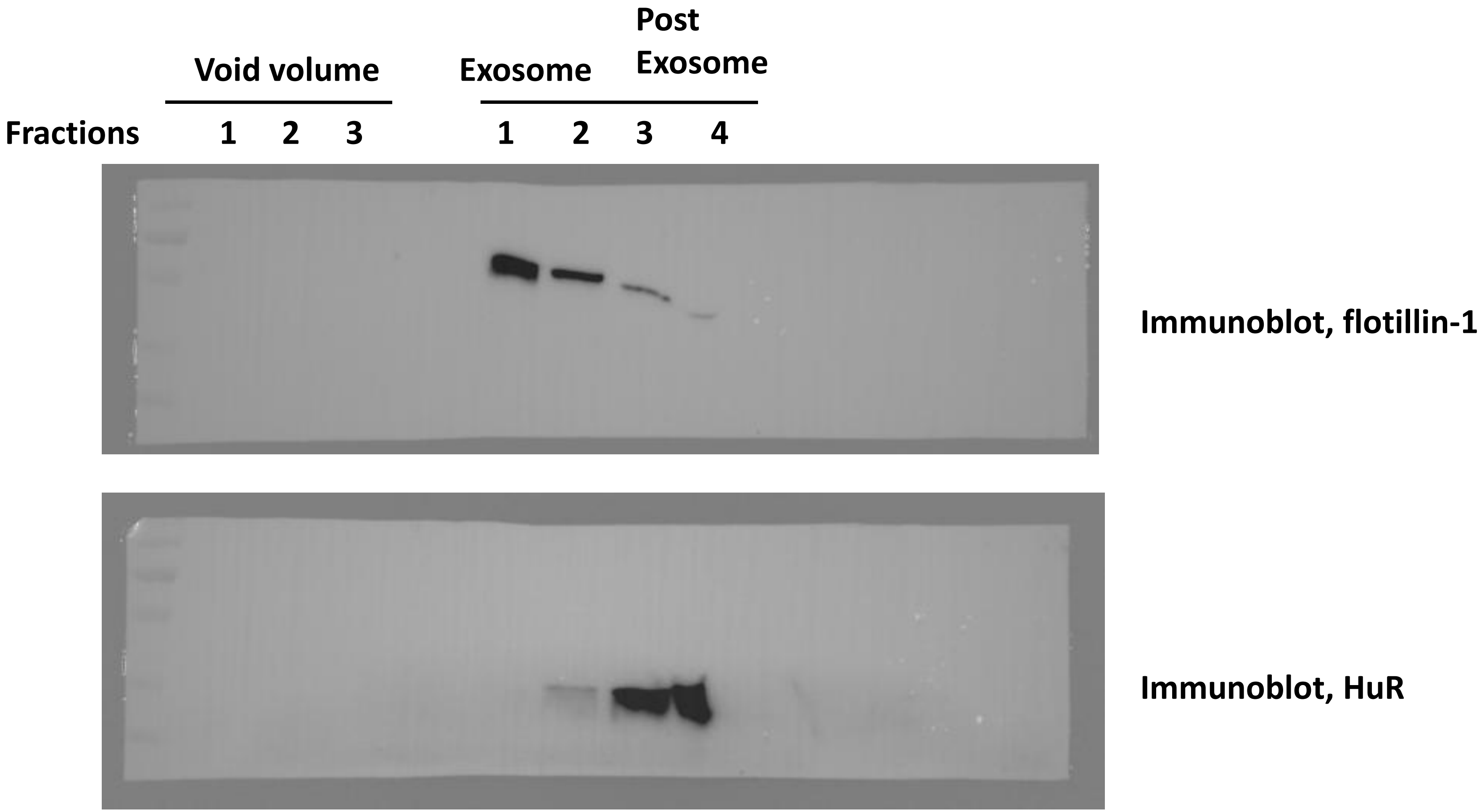

Supplementary Figure 27:
Original blots for Supplementary Figure 2

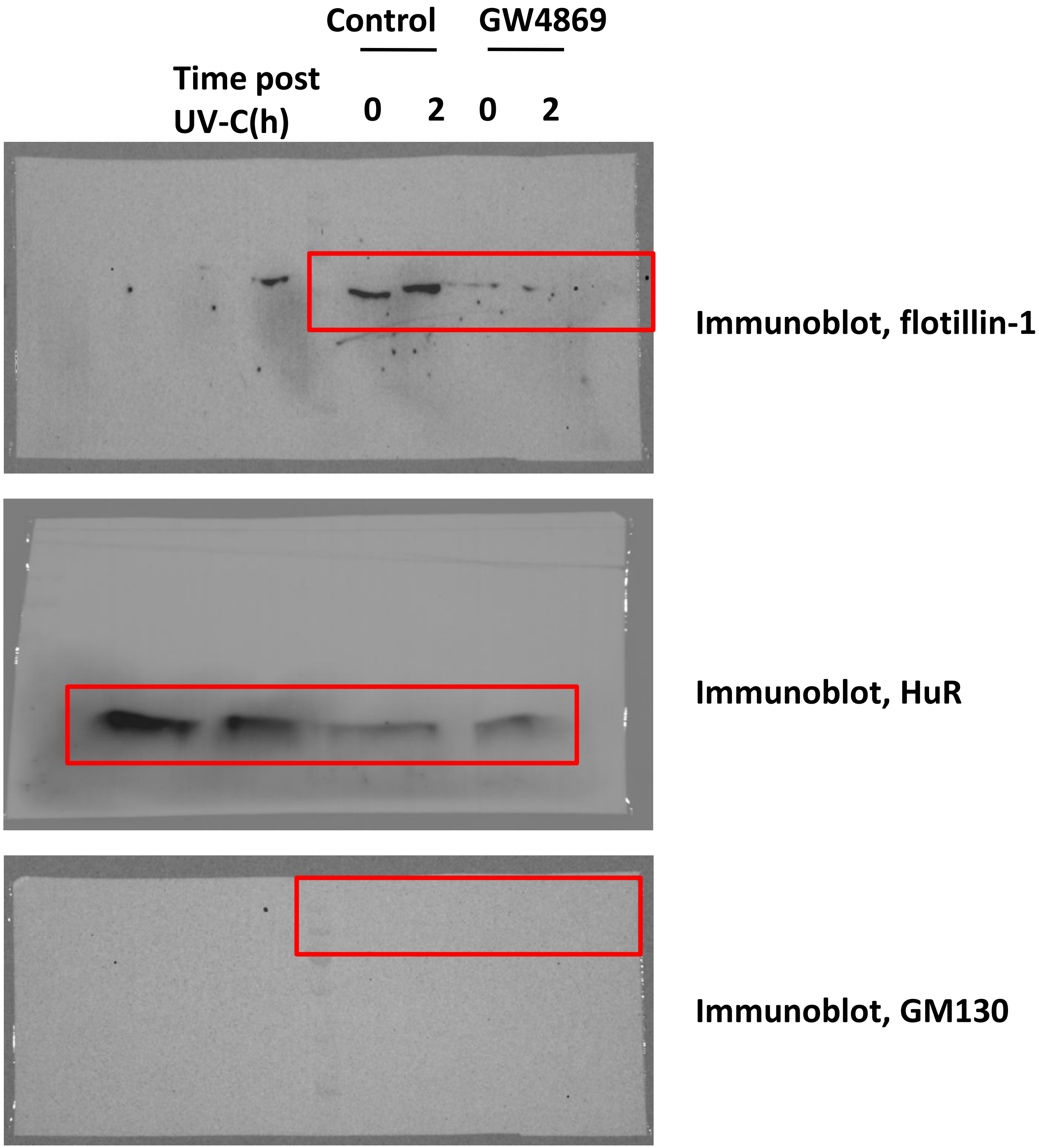

Supplementary Figure 28:
Original blots for Supplementary Figure 4

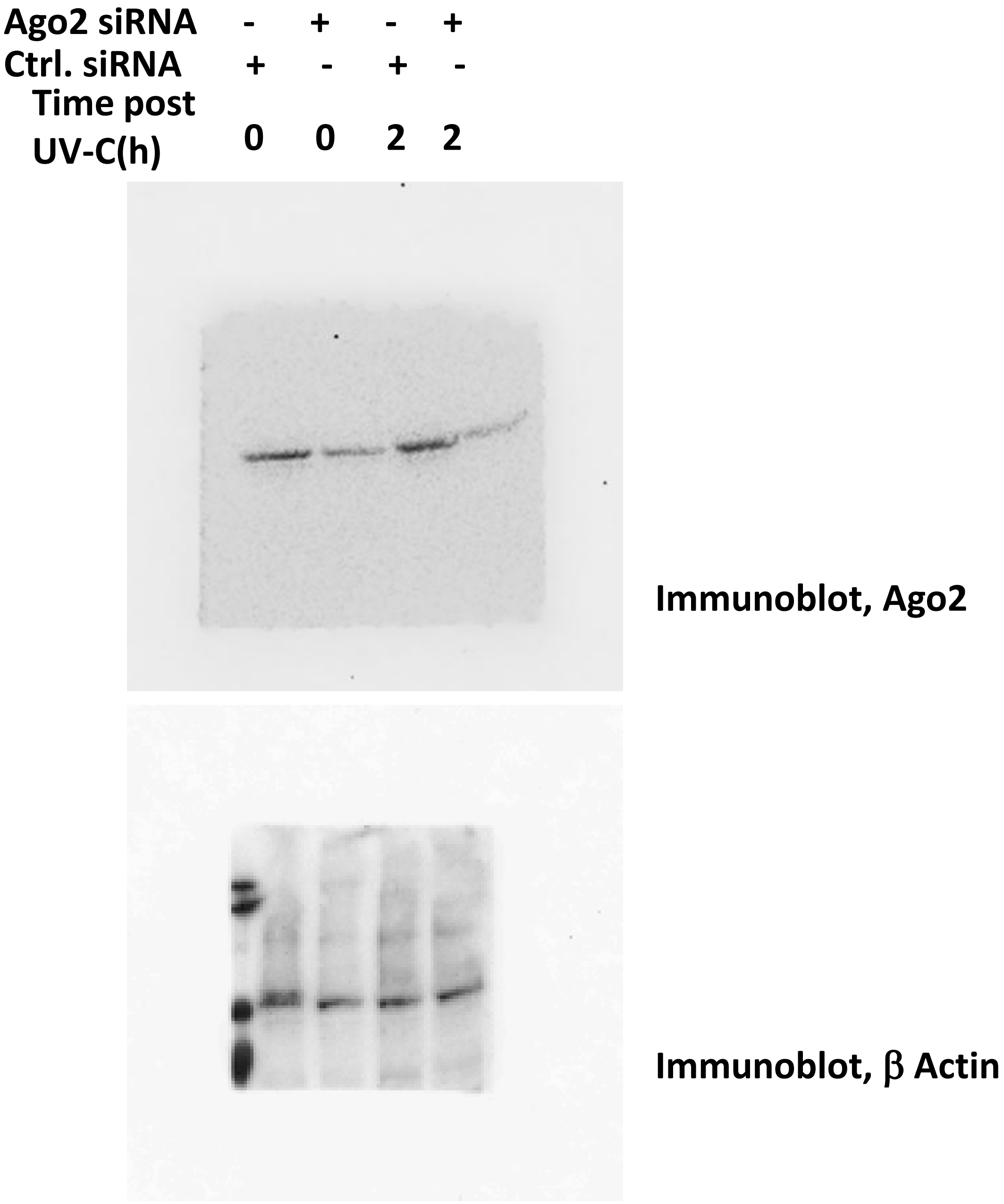

Supplementary Figure 29:
Original blots for Supplementary Figure 5

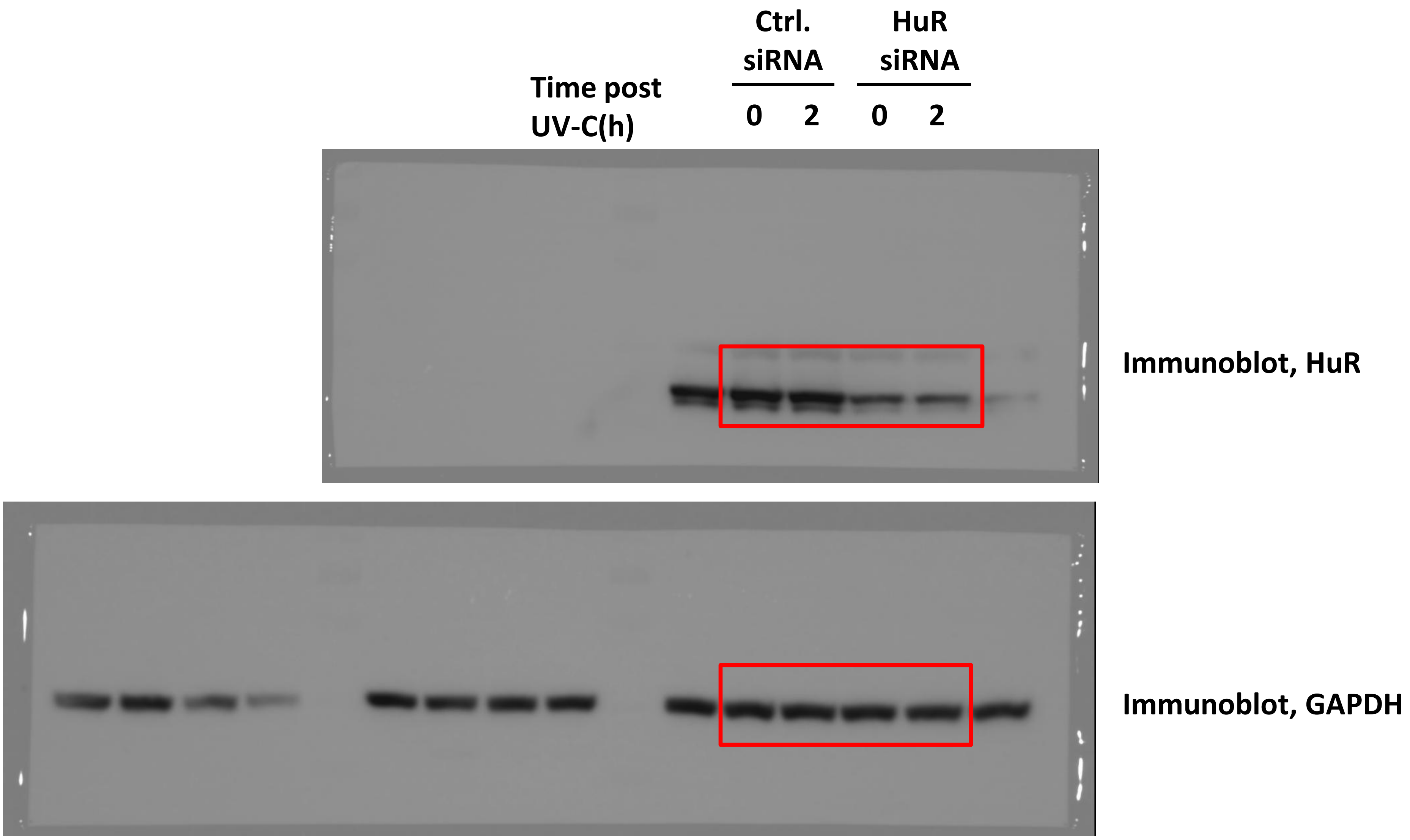

Supplementary Figure 30:
Original blots for Supplementary Figure 7

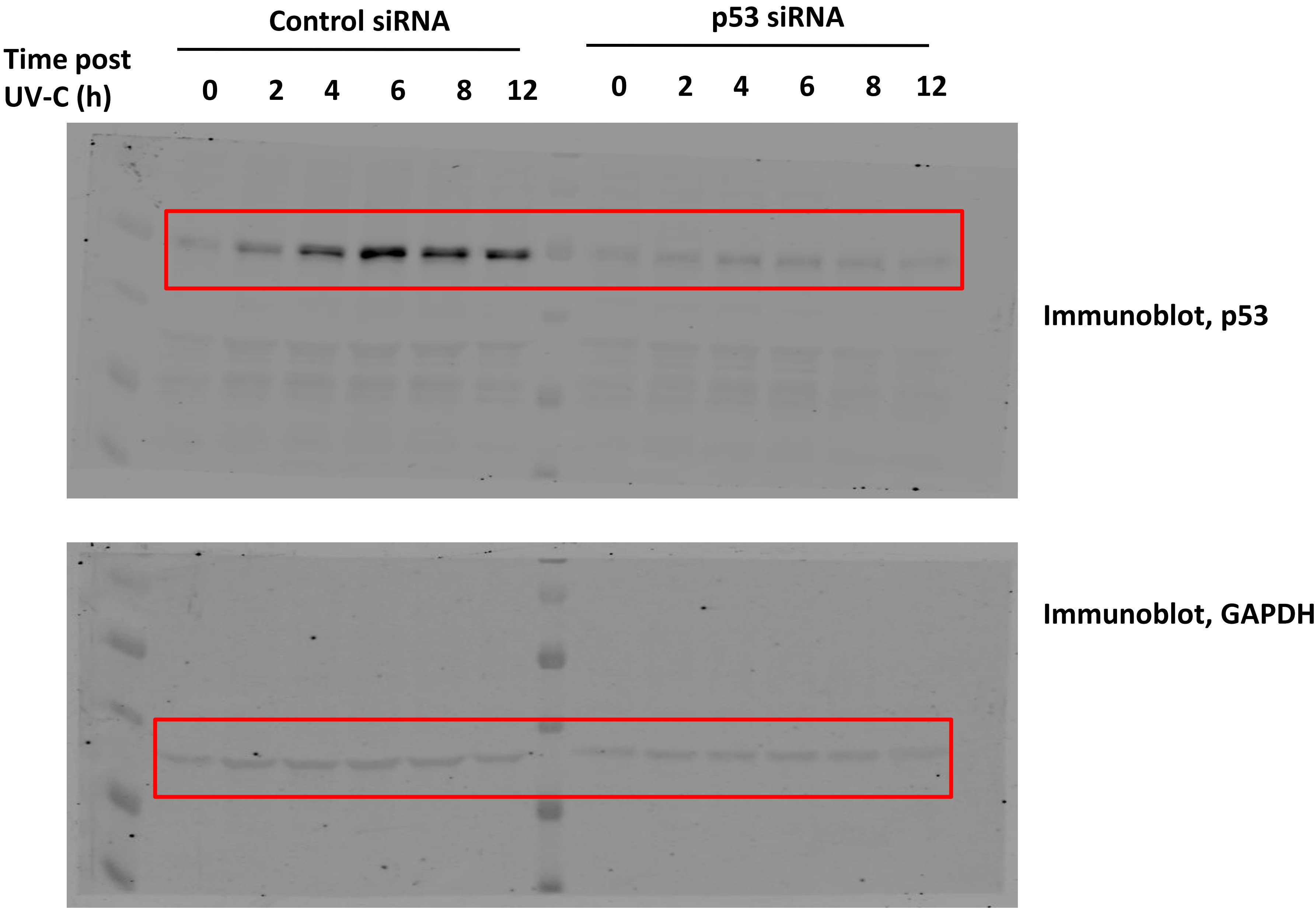

Supplementary Figure 31:
Original blots for Supplementary Figure 10

Supplementary Figure 11

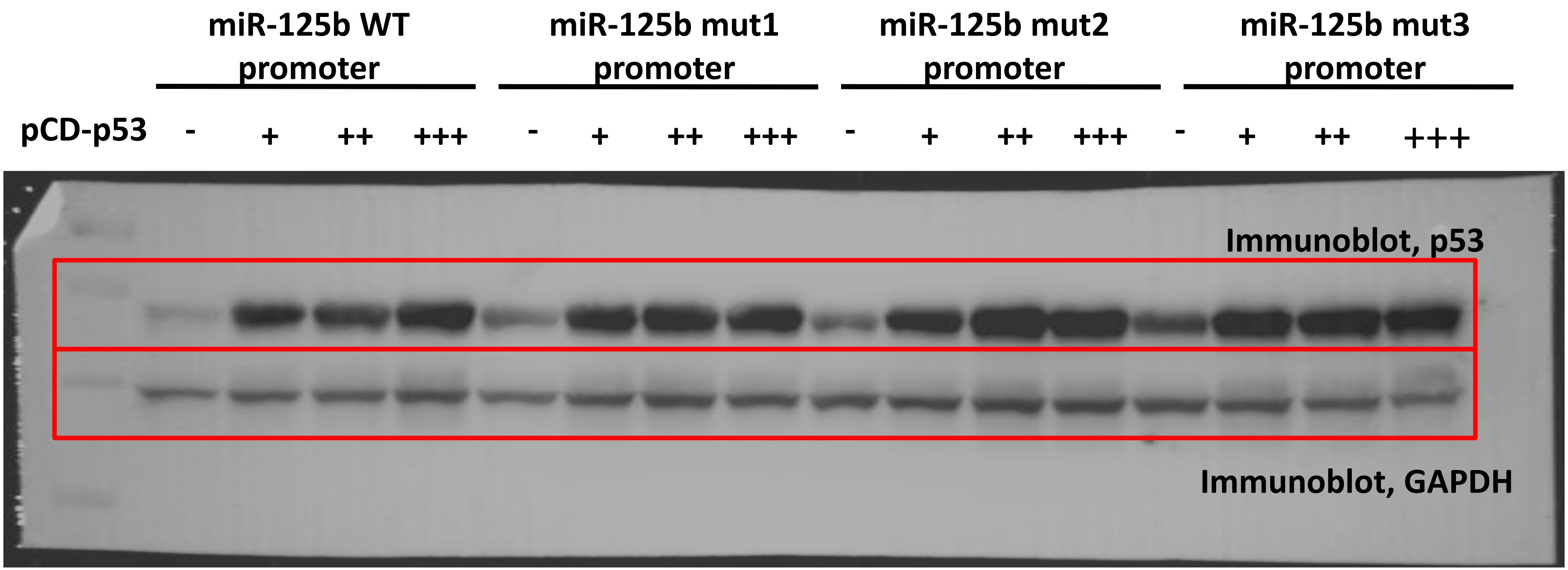

Supplementary Figure 32:
Original blots for Supplementary Figure 11

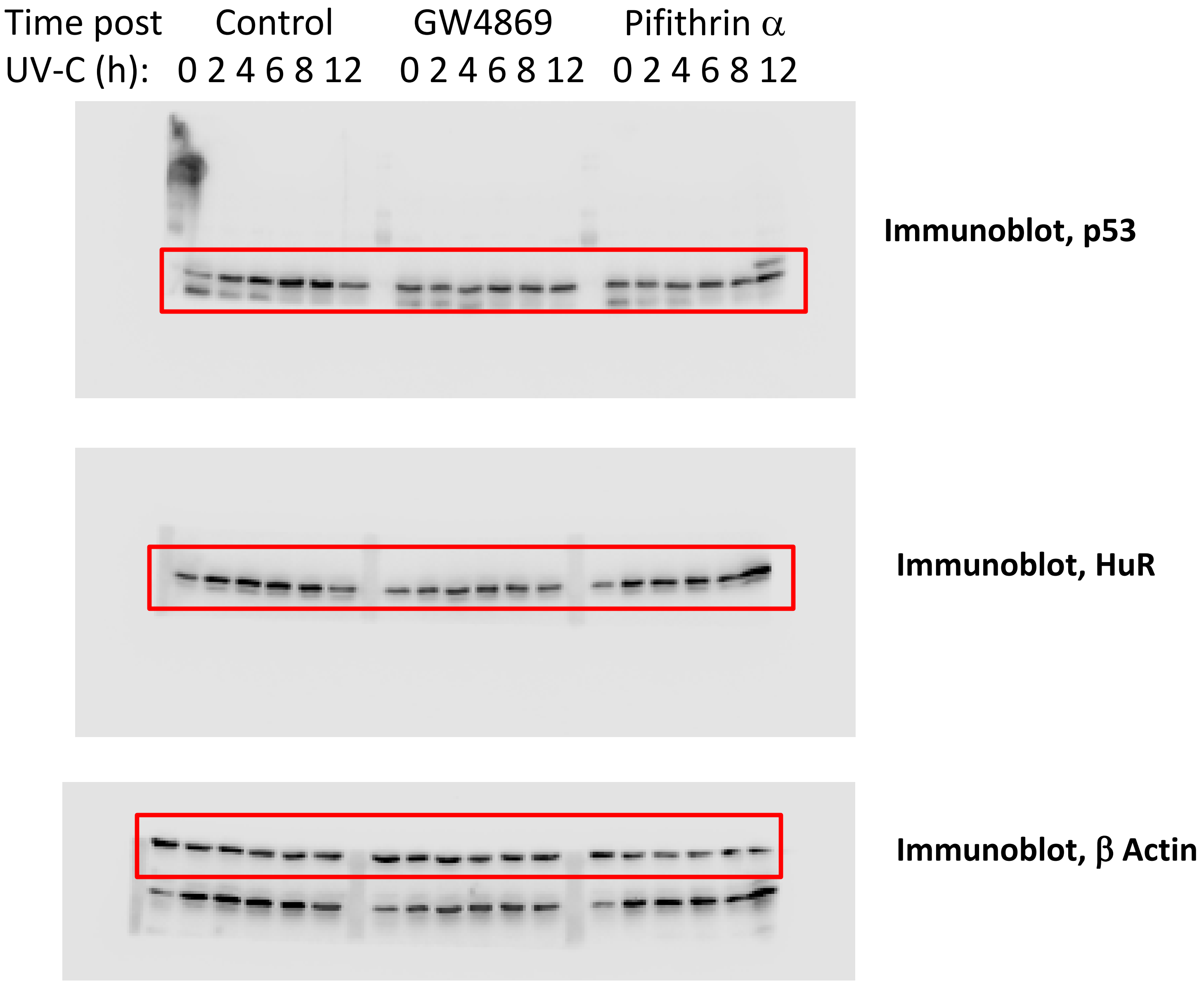

Supplementary Figure 33:
Original blots for Supplementary Figure 12
