# Supplementary material for: p53 and HuR combinatorially control the biphasic dynamics of microRNA-125b in response to genotoxic stress
Source: Commun Biol. 2023 Jan 27;6:110. doi: 10.1038/s42003-023-04507-9 (PMC9883498; doi:10.1038/s42003-023-04507-9)
Supplement: Supplementary file 2 — Description of Additional Supplementary Files [file 42003_2023_4507_MOESM2_ESM.pdf]

Description of Additional Supplementary Files

File name: Supplementary Data 1

Description: Table containing output of TFbind analysis.

File name: Supplementary Data 2

Description: The source data behind the graphs in the paper.
