# Supplementary material for: p53 and HuR combinatorially control the biphasic dynamics of microRNA-125b in response to genotoxic stress
Source: Commun Biol. 2023 Jan 27;6:110. doi: 10.1038/s42003-023-04507-9 (PMC9883498; doi:10.1038/s42003-023-04507-9)
Supplement: Supplementary file 5 — Reporting Summary [file 42003_2023_4507_MOESM5_ESM.pdf]

nature portfolio

Corresponding author(s): Partho Sarothi Ray

Last updated by author(s): Jan 10, 2023

Reporting Summary

Nature Portfolio wishes to improve the reproducibility of the work that we publish. This form provides structure for consistency and transparency in reporting. For further information on Nature Portfolio policies, see our Editorial Policies and the Editorial Policy Checklist.

Statistics

For all statistical analyses, confirm that the following items are present in the figure legend, table legend, main text, or Methods section.

n/a	Confirmed	
☐	☒	The exact sample size (*n*) for each experimental group/condition, given as a discrete number and unit of measurement
☐	☒	A statement on whether measurements were taken from distinct samples or whether the same sample was measured repeatedly
☐	☒	The statistical test(s) used AND whether they are one- or two-sided<br>*Only common tests should be described solely by name; describe more complex techniques in the Methods section.*
☐	☒	A description of all covariates tested
☐	☒	A description of any assumptions or corrections, such as tests of normality and adjustment for multiple comparisons
☐	☒	A full description of the statistical parameters including central tendency (e.g. means) or other basic estimates (e.g. regression coefficient) AND variation (e.g. standard deviation) or associated estimates of uncertainty (e.g. confidence intervals)
☐	☒	For null hypothesis testing, the test statistic (e.g. F, t, r) with confidence intervals, effect sizes, degrees of freedom and P value noted<br>*Give P values as exact values whenever suitable.*
☒	☐	For Bayesian analysis, information on the choice of priors and Markov chain Monte Carlo settings
☒	☐	For hierarchical and complex designs, identification of the appropriate level for tests and full reporting of outcomes
☒	☐	Estimates of effect sizes (e.g. Cohen's d, Pearson's r), indicating how they were calculated

Our web collection on statistics for biologists contains articles on many of the points above.

Software and code

Policy information about availability of computer code

Data collection	No software used
Data analysis	MS Excel (2007), GraphPad Prism 5, TFbind

For manuscripts utilizing custom algorithms or software that are central to the research but not yet described in published literature, software must be made available to editors and reviewers. We strongly encourage code deposition in a community repository (e.g. GitHub). See the Nature Portfolio guidelines for submitting code & software for further information.

Data

Policy information about availability of data

All manuscripts must include a data availability statement. This statement should provide the following information, where applicable:

- Accession codes, unique identifiers, or web links for publicly available datasets
- A description of any restrictions on data availability
- For clinical datasets or third party data, please ensure that the statement adheres to our policy

All data generated or analysed during this study are included in this published article (and its supplementary information files).

Human research participants

Policy information about studies involving human research participants and Sex and Gender in Research.

Reporting on sex and gender	*Use the terms sex (biological attribute) and gender (shaped by social and cultural circumstances) carefully in order to avoid confusing both terms. Indicate if findings apply to only one sex or gender; describe whether sex and gender were considered in study design whether sex and/or gender was determined based on self-reporting or assigned and methods used. Provide in the source data disaggregated sex and gender data where this information has been collected, and consent has been obtained for sharing of individual-level data; provide overall numbers in this Reporting Summary. Please state if this information has not been collected. Report sex- and gender-based analyses where performed, justify reasons for lack of sex- and gender-based analysis.*
Population characteristics	*Describe the covariate-relevant population characteristics of the human research participants (e.g. age, genotypic information, past and current diagnosis and treatment categories). If you filled out the behavioural & social sciences study design questions and have nothing to add here, write "See above."*
Recruitment	*Describe how participants were recruited. Outline any potential self-selection bias or other biases that may be present and how these are likely to impact results.*
Ethics oversight	*Identify the organization(s) that approved the study protocol.*

Note that full information on the approval of the study protocol must also be provided in the manuscript.

Field-specific reporting

Please select the one below that is the best fit for your research. If you are not sure, read the appropriate sections before making your selection.

☒ Life sciences ☐ Behavioural & social sciences ☐ Ecological, evolutionary & environmental sciences

For a reference copy of the document with all sections, see nature.com/documents/nr-reporting-summary-flat.pdf

Life sciences study design

All studies must disclose on these points even when the disclosure is negative.

Sample size	Sample size predetermination was not done. Data from a minimum of three independent replicates of each experiment has been included as per common convention in Life sciences.
Data exclusions	Each experiment has been repeated 7-8 times. Data from 3-5 experiments showing the most consistent results have been included for each experiment. Outlying data, which could be attributed to differences in experimental conditions, have been excluded.
Replication	Independent biological replicates have been done for each experiment for 7-8 times. All replicates have shown similar qualitative pattern, with quantitative variations within expected range. Data from 3-5 independent replicates have been included for all experiments.
Randomization	Cells seeded in wells were randomly allocated to experimental groups.
Blinding	Investigator blinding was not done as it is a mechanistic study.

Reporting for specific materials, systems and methods

We require information from authors about some types of materials, experimental systems and methods used in many studies. Here, indicate whether each material, system or method listed is relevant to your study. If you are not sure if a list item applies to your research, read the appropriate section before selecting a response.

Materials & experimental systems

n/a	Involved in the study	
☐	☒	Antibodies
☐	☒	Eukaryotic cell lines
☒	☐	Palaeontology and archaeology
☒	☐	Animals and other organisms
☒	☐	Clinical data
☒	☐	Dual use research of concern

Methods

n/a	Involved in the study	
☒	☐	ChIP-seq
☒	☐	Flow cytometry
☒	☐	MRI-based neuroimaging

Antibodies

Antibodies used	HuR (3A2, sc-5261, Santa Cruz Biotechnology), p53 (DO-1, sc-126 Santa Cruz Biotechnology), Flotillin-1 (D2V7J, Cell Signaling Technologies), GM-130 (D6B1, Cell Signaling), Rab7A (NBP2-24591SS, NovusBio), DDX5 (MAB6370, R & D Systems), Ago2 (C34C6, Cell Signaling) GAPDH (FL-335, Santa Cruz Biotechnology or A00192, GenScript), His (BB-AB0010, BioBharati Life Science) and β-actin (A00730, GenScript) primary antibodies. HRP- conjugated anti-rabbit (7074S, Cell Signaling) and anti-mouse (7076S, Cell Signaling), FITC-conjugated anti-mouse (AS001, ABclonal), Alexa Fluor 568- conjugated anti-rabbit (A11011, Life Technologies) secondary antibodies.
Validation	All primary antibodies used are commercially available and validated by the manufacturers as per datasheet provided by manufacturers.

Eukaryotic cell lines

Policy information about cell lines and Sex and Gender in Research

Cell line source(s)	MCF7 breast carcinoma cells from ATCC (HTB-22™), MCF 10A mammary epithelial cells from ATCC (CRL-10317™), MDA-MB-231 breast carcinoma cells from ATCC (HTB-26).
Authentication	Cell line was not authenticated.
Mycoplasma contamination	Cell line tested negative for mycoplasma contamination (by MycoAlert® Mycoplasma Detection Kit, Lonza BioSciences)
Commonly misidentified lines (See ICLAC register)	Not used
